# Effects of Exercise Alone or Combined With Cognitive Training and Vitamin D Supplementation to Improve Cognition in Adults With Mild Cognitive Impairment: A Randomized Clinical Trial

**DOI:** 10.1001/jamanetworkopen.2023.24465

**Published:** 2023-07-20

**Authors:** Manuel Montero-Odasso, Guangyong Zou, Mark Speechley, Quincy J. Almeida, Teresa Liu-Ambrose, Laura E. Middleton, Richard Camicioli, Nick W. Bray, Karen Z. H. Li, Sarah Fraser, Frederico Pieruccini-Faria, Nicolas Berryman, Maxime Lussier, J. Kevin Shoemaker, Surim Son, Louis Bherer

**Affiliations:** 1 Gait and Brain Lab, Parkwood Institute, Lawson Health Research Institute, London, Ontario, Canada; 2 Department of Medicine, Division of Geriatric, Schulich School of Medicine and Dentistry, University of Western Ontario, London, Ontario, Canada; 3 Department of Epidemiology and Biostatistics, Schulich School of Medicine & Dentistry, University of Western Ontario, London, Ontario, Canada; 4 Robarts Research Institute, University of Western Ontario, London, Ontario, Canada; 5 Carespace Health & Wellness, Waterloo, Ontario, Canada; 6 Movement Disorders Research & Rehabilitation Centre, Department of Kinesiology and Physical Education, Wilfrid Laurier University, Waterloo, Ontario, Canada; 7 Department of Physical Therapy, University of British Columbia, Vancouver, British Columbia, Canada; 8 Department of Kinesiology and Health Sciences, University of Waterloo, Waterloo, Ontario, Canada; 9 Department of Medicine, Division of Neurology, University of Alberta, Edmonton, Alberta, Canada; 10 School of Kinesiology, Faculty of Health Sciences, University of Western Ontario, London, Ontario, Canada; 11 PERFORM Centre and Department of Psychology, Concordia University, Montréal, Quebec, Canada; 12 Faculty of Health Sciences, Interdisciplinary School of Health Sciences, University of Ottawa, Ontario, Canada; 13 Département des sciences de l’activité physique Université du Québec à Montréal, Montréal, Quebec, Canada; 14 Research Centre, Institut Universitaire de Gériatrie de Montréal, Montréal, Quebec, Canada; 15 Integrated Health and Social Services University Network for South-Central Montreal, Montreal, Quebec, Canada; 16 Research Centre, Montreal Heart Institute, and Department of Medicine, University of Montréal, Montréal, Quebec, Canada

## Abstract

**Importance:**

Exercise, cognitive training, and vitamin D may enhance cognition in older adults with mild cognitive impairment (MCI).

**Objective:**

To determine whether aerobic-resistance exercises would improve cognition relative to an active control and if a multidomain intervention including exercises, computerized cognitive training, and vitamin D supplementation would show greater improvements than exercise alone.

**Design, Setting, and Participants:**

This randomized clinical trial (the SYNERGIC Study) was a multisite, double-masked, fractional factorial trial that evaluated the effects of aerobic-resistance exercise, computerized cognitive training, and vitamin D on cognition. Eligible participants were between ages 65 and 84 years with MCI enrolled from September 19, 2016, to April 7, 2020. Data were analyzed from February 2021 to December 2022.

**Interventions:**

Participants were randomized to 5 study arms and treated for 20 weeks: arm 1 (multidomain intervention with exercise, cognitive training, and vitamin D), arm 2 (exercise, cognitive training, and placebo vitamin D), arm 3 (exercise, sham cognitive training, and vitamin D), arm 4 (exercise, sham cognitive training, and placebo vitamin D), and arm 5 (control group with balance-toning exercise, sham cognitive training, and placebo vitamin D). The vitamin D regimen was a 10 000 IU dose 3 times weekly.

**Main Outcomes and Measures:**

Primary outcomes were changes in ADAS-Cog-13 and Plus variant at 6 months.

**Results:**

Among 175 randomized participants (mean [SD] age, 73.1 [6.6] years; 86 [49.1%] women), 144 (82%) completed the intervention and 133 (76%) completed the follow-up (month 12). At 6 months, all active arms (ie, arms 1 through 4) with aerobic-resistance exercise regardless of the addition of cognitive training or vitamin D, improved ADAS-Cog-13 when compared with control (mean difference, −1.79 points; 95% CI, −3.27 to −0.31 points; *P* = .02; *d* = 0.64). Compared with exercise alone (arms 3 and 4), exercise and cognitive training (arms 1 and 2) improved the ADAS-Cog-13 (mean difference, −1.45 points; 95% CI, −2.70 to −0.21 points; *P* = .02; *d* = 0.39). No significant improvement was found with vitamin D. Finally, the multidomain intervention (arm 1) improved the ADAS-Cog-13 score significantly compared with control (mean difference, −2.64 points; 95% CI, −4.42 to −0.80 points; *P* = .005; *d* = 0.71). Changes in ADAS-Cog-Plus were not significant.

**Conclusions and Relevance:**

In this clinical trial, older adults with MCI receiving aerobic-resistance exercises with sequential computerized cognitive training significantly improved cognition, although some results were inconsistent. Vitamin D supplementation had no effect. Our findings suggest that this multidomain intervention may improve cognition and potentially delay dementia onset in MCI.

**Trial Registration:**

ClinicalTrials.gov Identifier: NCT02808676

## Key Points

**Question:**

Does a multidomain intervention of aerobic-resistance exercises with cognitive training and vitamin D improve cognition in older adults with mild cognitive impairment?

**Findings:**

In this randomized clinical trial including 175 Canadian adults aged 65 to 84 years, a 20-week multidomain intervention of aerobic-resistance exercises with computerized cognitive training had a larger effect in improving cognition than exercise interventions alone, and these improvements were maintained at 12-month follow-up. Vitamin D addition did not enhance the effect.

**Meaning:**

These findings suggest that pairing aerobic and resistance exercises with sequential computerized cognitive training may improve cognition in older adults with mild cognitive impairment.

## Teaser

This randomized clinical trial examines the effectiveness of aerobic-resistance exercise with and without computerized cognitive training and vitamin D supplements to improve cognition among older adults with mild cognitive impairment (MCI).

## Introduction

Over 50 million worldwide lived with dementia in 2021, with associated costs exceeding $800 billion US.^1^ There is no cure for dementia, but there has been a fundamental shift to target those at risk using nonpharmacological and lifestyle interventions to improve cognition and potentially delay dementia onset.^2,3^ Mild cognitive impairment (MCI) is an intermediate state between normal cognitive aging and early dementia, the optimal period to intervene with preventive strategies and early treatments.^4^

Both aerobic exercise and resistance training have been demonstrated to improve cognition in older adults, although the benefits of combining these 2 modalities are unclear.^5^ Computer-based cognitive training also improves cognition in older adults through the repeated engagement of cognitive processes using challenging and preferably adaptive tasks.^6^ Furthermore, vitamin D in addition to exercise and cognitive training has been argued to enhance cognition due to its neuroprotective attributes.^7^ Thus, providing these interventions together, as a multidomain treatment, has the potential to delay progression from MCI to dementia.^8,9^

Previous multidomain intervention trials in healthy older adults have demonstrated primary efficacy in improving cognition, as in the FINGER (Finnish Geriatric Intervention Study to Prevent Cognitive Impairment and Disability) trial,^10^ and post hoc analyses of specific higher-risk subgroups in the MAPT (Multidomain Alzheimer Preventive Trial)^11^ and PREDIVA (Prevention of Dementia by Intensive Vascular Care) trials.^12^ However, the effect of multidomain interventions in cognitively impaired populations remains elusive. The recent MEDEX (Mindfulness-Based Stress Reduction, Health Education and Exercise) factorial trial failed to show an effect of combining mindfulness training and exercise to improve cognition in older adults with subjective cognitive concerns.^13^ In MCI populations, multidomain trials coupling exercise with other interventions have shown mixed results.^14^ In particular, 2 previous randomized factorial trials demonstrated that combining exercise with cognitive training was less effective than exercises alone in improving cognition.^15,16^ Similarly, the sustainability of any cognitive improvements following interventions have not yet been established. The SYNERGIC Trial^17^ (Synchronizing Exercises, Remedies in Gait and Cognition) was conducted to evaluate the cognitive benefits of an aerobic-resistance exercise regime, alone or in addition to computer-based cognitive training, and vitamin D supplementation in older adults with MCI.

## Methods

### Study Design

The SYNERGIC Trial was a double-masked, randomized trial with a fractional factorial design to evaluate the effect of 20-week multidomain interventions on cognition in older adults with MCI (protocol available in Supplement 1). Assessments occurred at baseline, postintervention (month 6), and follow-up (month 12). The trial was conducted at 5 Canadian academic institutions (Western University [sponsor site], University of Waterloo, Wilfrid Laurier University, University of Montreal, and University of British Columbia). Ethics approval was provided by each institution’s review boards. All participants provided written informed consent. SYNERGIC adhered to the Consolidated Standards of Reporting Trials Extension (CONSORT Extension) reporting guidelines, as extended to nonpharmacologic interventions.^18^

### Participants

Participants were aged 60 to 85 years, recruited from the community, who fulfilled MCI criteria^19^: (1) subjective cognitive concerns; (2) objective cognitive impairment in memory, executive function, attention, and/or language; (3) preserved activities of daily living; and (4) absence of dementia (eMethods in Supplement 2). Exclusion included major depression, schizophrenia, substance abuse, parkinsonism, conditions affecting gait (eg, severe osteoarthritis, previous stroke), exercise program participation, and taking vitamin D doses greater than 1000 IU per day, cognitive enhancers, or anticholinergics. Ethnicity of participants was assessed using the CCNA ethnicity questionnaire, to characterize minority representation in our study. Full eligibility criteria are detailed in our protocol^17^ (Supplement 1).

### Randomization and Masking

Participants were randomly assigned into arms in a 1:1:1:1:1 ratio using a central computer-generated random number sequence in blocks of 5: arm 1 (aerobic-resistance exercise, cognitive training, and vitamin D); arm 2 (exercise, cognitive training, and placebo vitamin D); arm 3 (exercise, sham cognitive training, and vitamin D); arm 4 (exercise, sham cognitive training, and placebo vitamin D); and arm 5 (balance and toning exercise, sham cognitive training, and placebo vitamin D). A research pharmacist assigned vitamin D or placebo capsules as kits in compliance with the randomization list(s).

Arm allocation was not disclosed to participants, who were asked not to discuss the intervention during training sessions. Outcome assessors were masked to allocation and not involved in the interventions.

### Procedures

Participants in all 5 study arms completed group-training sessions 3 times per week for 20 weeks. Each session lasted 90 minutes and included 30 minutes of cognitive training (active or sham), followed by 60 minutes of aerobic-resistance or the control exercise (balance and toning). All participants received a capsule of vitamin D (a 10 000 IU dose) or matching placebo 3 times per week for 20 weeks.

Participants performed cognitive training (Neuropeak; detailed description in eMethods in Supplement 2) or sham cognitive training on a computerized tablet (Apple). Neuropeak delivered 2 visuomotor tasks targeting working memory and attention separately and concurrently. Level of difficulty increased over time, and participants received individually tailored continuous feedback on performance.^20^ Sham cognitive training included alternating between 2 tasks (touristic search and video watching) with the same time exposure as the intervention training.

The supervised progressive exercise program combined aerobic and resistance training based on exercise prescription for older adults (eMethods in Supplement 2).^21^ Exercise progress and intensity were monitored using the Borg Rating of Perceived Exertion.^22^ Control exercises included stretching, balance, and toning exercises that did not progress in volume or intensity. Exercise groups had approximately a trainer-to-participants ratio of 1:4. To maximize intervention fidelity, all trainers followed the same manual of procedures and met biweekly to review progress. Participants attended at least 85% of sessions and were followed up by telephone if absent.

### Outcomes

Change (baseline to postintervention) in cognitive function was assessed using 2 primary outcomes, the Alzheimer Disease Assessment Scale Cognitive 13 (ADAS-Cog-13) and the Plus variant.^23^ The ADAS-Cog-13 consists of 13 cognitive tests assessing various cognitive domains, while the Plus variant is considered more sensitive to executive function by incorporating 5 additional tests (eMethods in Supplement 2). Scores range from 0 to 85, with higher scores indicating worse cognition. ADAS-Cog-13 is recommended as a robust primary outcome in MCI trials for its responsiveness.^23^ However, the rationale for including the Plus variant relates to previous studies demonstrating that exercise may exert greater effects on executive function than other cognitive domains.^24^ Significant improvements in either ADAS-Cog-13 or the Plus variant at month 6 was considered proof of efficacy.^25,26^ Individual ADAS-Cog-13 and Plus items were analyzed as secondary cognitive outcomes (statistical analysis plan in Supplement 1).

### Sample Size Calculation

Assuming the primary outcome data follow approximately a normal distribution, a trial with 1:4 ratio (control:exercise arms) would require 170 participants to detect an effect size of 0.54 with 80% power at a 2-sided, 5% significance level. This sample size was inflated to 200 (40 per arm) to account for potential attrition of 15%.

### Statistical Analysis

**Figure 1.**
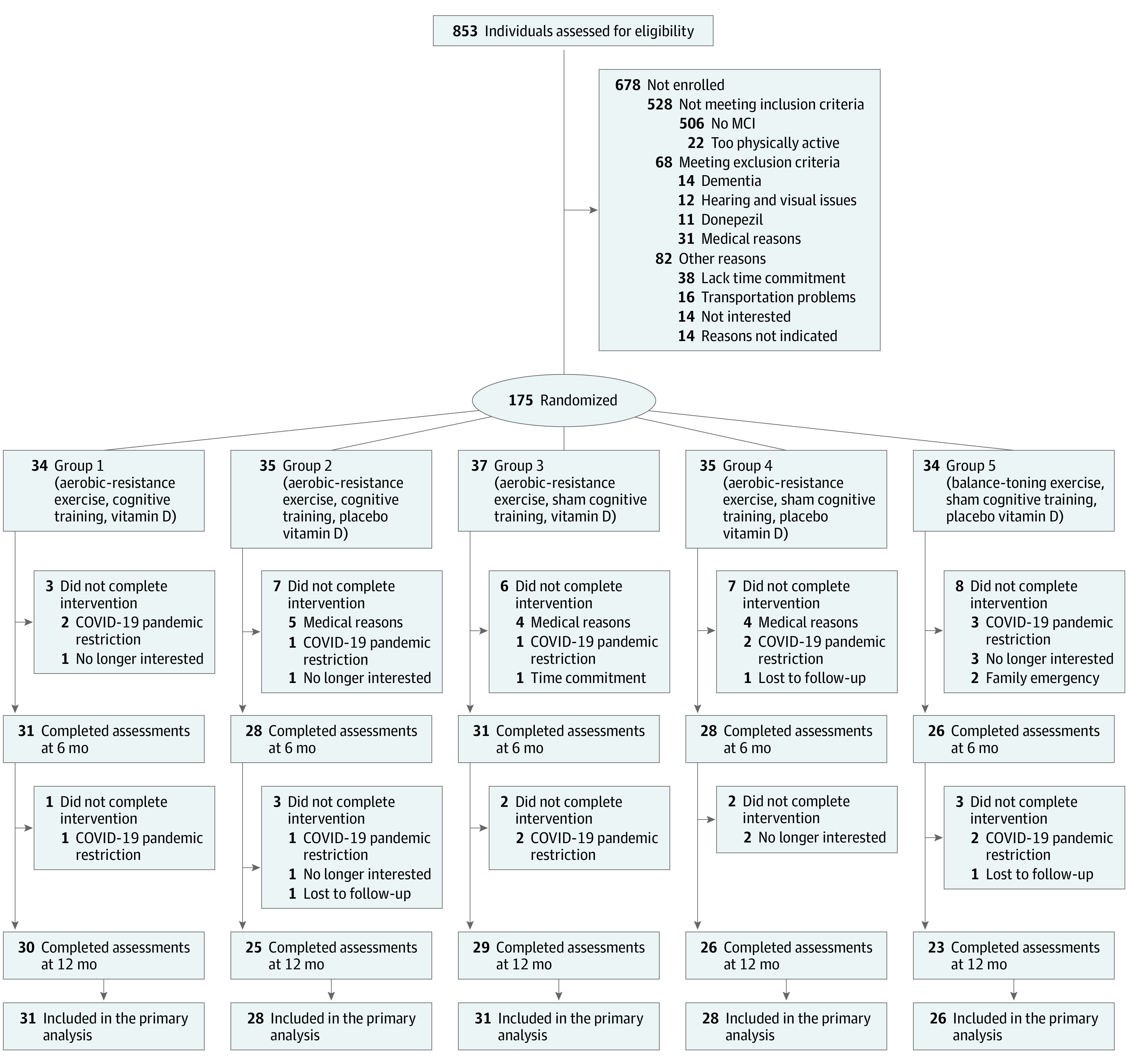
SYNERGIC Trial Consortium Flowchart MCI indicates mild cognitive impairment.

Following the intention-to-treat principle, all randomized individuals were included in the primary analysis (Figure 1; statistical analysis plan available in Supplement 1). Outcomes were analyzed using a linear mixed model approach with repeated measures.^27^ Models were fitted with participant-specific random intercept and fixed effects of time, intervention arm, and time-by-intervention-arm interaction, and adjusted for age, sex, education, and comorbidities.

Between-arms comparisons followed our protocol and statistical analysis plan, which aligns with the recommended analysis and reporting of factorial trials.^28,29^ For comparisons, what have been described as “inside the table” analyses were employed to compare each intervention arm with the control arm (arm 5), while “at the margin” analyses were conducted by pooling arms in prespecified combinations to examine potential synergism. Participants that received both exercise and cognitive intervention (arms 1 and 2) and participants that received exercise but sham cognitive intervention (arms 3 and 4) were compared with the control (arm 5). To facilitate the interpretation of treatment effects on primary and secondary outcomes, we calculated effect sizes, defined as differences divided by standard deviation,^30^ and the proportion of participants with a clinically significant improvement in primary outcomes.

The durability of the intervention effect from baseline and postintervention to follow-up (month 12) was assessed for primary and secondary outcomes using linear mixed models with all 3 time points.

Adjustments for multi-arm comparisons were not made as the trial is intended to compare the intervention effect with the control rather than pairwise, and no adjustments for having 2 primary outcomes were executed.^31^ Interpretation of statistical tests were based on a 2-sided 5% significance level. All analyses were conducted in SPSS version 23.0 (SPSS Inc) and R version 3.5.1 (R Project for Statistical Computing).

## Results

Of 853 individuals screened for eligibility, 175 (20%) were randomized (mean [SD] age, 73.1 [6.6] years; 86 [49.1%] female; 79 [83.2%] White). Cognitive assessment revealed a mean (SD) Montreal Cognitive Assessment (MoCA) score of 22.6 (3.2), ADAS-Cog-13 of 15.2 (6.8), and global Clinical Dementia Rating (CDR) of 0.4 (0.2) (Table 1). Baseline characteristics were similar across the intervention arms, except that arm 5 (control) participants had 1.8 more years of education than the full sample mean (17.2 [4.9] years vs 15.4 [3.7] years).

**Table 1.** Baseline Characteristics of Participants

Characteristics	Total sample (N = 175)	Arm 1 (exercise, cognitive training, and vitamin D) (n = 34)	Arm 2 (exercise, cognitive training, and placebo vitamin D) (n = 35)	Arm 3 (exercise, sham cognitive training, vitamin D) (n = 37)	Arm 4 (exercise, sham cognitive training, placebo vitamin D) (n = 35)	Arm 5 (control) (n = 34)^a^
Demographic characteristics						
Age, mean (SD), y	73.1 (6.6)	73.1 (6.1)	72.4 (7.3)	73.1 (7.6)	73.1 (5.9)	73.8 (6.2)
Sex, No. (%)						
Men	89 (50.9)	13 (38.2)	19 (54.3)	17 (45.9)	23 (65.7)	17 (50.0)
Women	86 (49.1)	21 (61.8)	16 (45.7)	20 (54.1)	12 (34.3)	17 (50.0)
Education, mean (SD), y	15.4 (3.7)	14.7 (2.6)	15.1 (3.3)	14.7 (2.6)	15.3 (4.4)	17.2 (4.9)
Postsecondary education or more, No. (%)	133 (76.0)	25 (73.5)	27 (77.1)	26 (70.3)	25 (71.4)	30 (88.2)
Clinical characteristics						
Total comorbidities, mean (SD)	4.5 (2.4)	4.7 (2.7)	4.5 (2.0)	4.6 (2.9)	4.2 (2.4)	4.5 (2.1)
Total medications, mean (SD)	5.9 (3.8)	5.8 (4.1)	6.0 (3.8)	6.6 (3.8)	5.5 (3.6)	5.4 (3.6)
Body mass index, mean (SD)	28.0 (7.9)	26.6 (4.4)	27.6 (6.0)	27.3 (5.4)	29.2 (10.7)	29.6 (11.4)
Systolic blood pressure, mean (SD), mm Hg	136.0 (18.9)	138.5 (19.8)	140.1 (21.0)	131.2 (19.5)	131.8 (15.2)	138.2 (17.6)
Diastolic blood pressure, mean (SD), mm Hg	79.1 (11.0)	81.3 (9.8)	82.4 (10.6)	77.0 (9.5)	74.6 (13.5)	80.2 (10.2)
Serum vitamin D levels, mean (SD), nmol/L	77.5 (26.5)	81.0 (24.7)	81.1 (19.2)	77.8 (28.5)	74.3 (33.6)	72.8 (26.1)
*APOE4* carriers, No. (%)	24 (29.6)^b^	7 (41.2)	4 (21.1)	3 (20.0)	6 (35.3)	4 (30.8)
Amnestic MCI, No. (%)	18 (10.3)	2 (5.9)	3 (8.6)	7 (18.9)	5 (14.3)	1 (2.9)
Amnestic multidomain MCI, No. (%)	74 (42.3)	12 (35.3)	16 (45.7)	15 (40.5)	15 (42.9)	16 (47.1)
Nonamnestic MCI, No. (%)	79 (45.1)	20 (58.8)	16 (45.7)	14 (37.8)	13 (37.1)	16 (47.1)
Global cognition						
MMSE score, mean (SD)^c^	27.0 (2.2)	27.4 (2.1)	27.2 (2.2)	26.3 (2.3)	26.9 (2.4)	27.0 (2.2)
MoCA score, mean (SD)^d^	22.6 (3.2)	23.3 (3.5)	22.4 (3.7)	22.4 (3.0)	22.3 (3.1)	22.3 (3.2)
ADAS-Cog-13 score, mean (SD)^e^	15.2 (6.8)	15.3 (8.0)	14.5 (6.1)	16.6 (7.6)	15.6 (6.6)	13.7 (5.3)
CDR global score, mean (SD)^f^	0.4 (0.2)	0.4 (0.2)	0.4 (0.2)	0.4 (0.2)	0.5 (0.2)	0.4 (0.2)
CDR sum of the boxes score, mean (SD)^g^	1.3 (0.9)	1.1 (0.7)	1.3 (1.0)	1.6 (1.1)	1.6 (0.8)	1.1 (0.8)
GDS-30 score, mean (SD)^h^	6.6 (5.2)	6.6 (5.0)	7.1 (4.9)	4.4 (3.2)	7.4 (5.6)	7.6 (6.7)
Cognitive subdomains^i^						
Memory						
Word recognition score, mean (SD)^j^	2.1 (2.0)	2.2 (2.6)	1.8 (1.7)	2.5 (1.9)	2.2 (2.3)	1.7 (1.3)
Orientation score, mean (SD)^k^	0.6 (0.7)	0.7 (1.2)	0.7 (0.7)	0.6 (0.9)	0.5 (0.8)	0.4 (0.6)
Immediate word recall score, mean (SD)^l^	4.1 (1.4)	4.0 (1.5)	3.8 (1.6)	4.4 (1.5)	4.2 (1.1)	4.0 (1.3)
Delayed word recall score, mean (SD)^m^	4.9 (2.4)	4.4 (2.5)	5.1 (2.4)	5.5 (2.5)	4.9 (2.2)	4.3 (2.0)
Processing Speed						
Trail making A, mean (SD), s^n^	41.3 (15.0)	40.8 (16.7)	38.8 (11.3)	43.3 (16.4)	42.0 (17.0)	41.5 (13.2)
Executive function						
Trail making B, mean (SD), s^o^	116.7 (61.8)	103.7 (54.7)	103.3 (58.7)	121.8 (66.3)	130.8 (75.4)	124.5 (49.7)
Attention						
Digit span forward score, mean (SD)^p^	10.4 (3.7)	10.7 (3.8)	10.5 (3.6)	10.7 (4.2)	10.2 (3.4)	10.0 (3.4)
Digit span backward score, mean (SD)^q^	6.8 (2.8)	7.2 (2.8)	6.9 (2.5)	6.8 (3.4)	6.5 (2.6)	6.5 (2.6)
Functionality						
Instrumental activities of daily living score, mean (SD)^r^	22.1 (1.9)	22.4 (1.2)	22.2 (1.9)	21.9 (2.1)	21.9 (1.9)	22.0 (2.1)
Physical activity score, mean (SD)^s^	111.6 (58.6)	119.5 (55.0)	114.7 (67.3)	119.6 (61.3)	99.5 (53.1)	103.8 (54.9)
SPPB score, mean (SD)^t^	10.0 (1.65)	10.3 (1.48)	10.3 (1.59)	9.81 (1.64)	9.75 (1.61)	9.87 (1.96)
Gait speed, mean (SD), cm/s^u^	117 (21.6)	118 (23.0)	122 (20.2)	112 (19.2)	117 (24.0)	115 (21.6)

Abbreviations: ADAS-Cog-13, Alzheimer Disease Assessment Cognitive 13-item; CDR, Clinical Dementia Rating scale; GDS-30, Geriatric Depression Scale; MCI, mild cognitive impairment; MoCA, Montreal Cognitive Assessment; MMSE, Mini-Mental State Evaluation; SPPB, Short Physical Performance Battery.

^a^ Control includes balance-toning exercise, sham cognitive training, and placebo vitamin D.

^b^ Data are available for 79 participants.

^c^ MMSE ranges from 0 to 30; score of ≤24 indicate cognitive impairment.

^d^ MoCA ranges from 0 to 30; score of ≤26 indicate cognitive impairment.

^e^ ADAS-Cog-13 ranges from 0 to 85; higher scores indicate severe cognitive impairment.

^f^ CDR global scale ranges from 0 to 3; score of 0 suggest no cognitive impairment, 0.5 suggest mild cognitive impairment, 1 suggest mild dementia, 2 suggest moderate dementia, and 3 suggest severe dementia.

^g^ CDR Sum of box scale ranges from 0 to 18; score of 0 indicate normal cognition, 0.5 to 4 indicate mild cognitive impairment, 4.5 to 9.0 indicate mild dementia, 9.5 to 15.5 indicate moderate dementia, and 16.0 to 18.0 indicate severe dementia.

^h^ GDS-30 ranges from 0 to 30. The scores measure depressive symptom, and higher scores indicate severe depressive symptom.

^i^ Subdomain of ADAS-Cog-13 and Plus.

^j^ Word recognition ranges from 0 to 12, indicating the number of incorrect recognition of words. Higher scores suggest greater cognitive impairment.

^k^ Orientation ranges from 0 to 8. The participants guessed date, place, and time. The scores are the number of incorrect responses, and higher scores suggest greater cognitive impairment.

^l^ Immediate word recall from the CERAD test ranges from 0 to 10. The participants recalled as many words as possible from a list of 10 words. The scores represent the mean number of words not recalled from 3 trials. Higher scores suggest greater cognitive impairment.

^m^ Delayed word recall from the CERAD test ranges from 0 to 10. The participants recalled as many as possible from the list of 10 words used in immediate word recall after 5 minutes. The scores represent the mean number of words not recalled from 3 trials. Higher scores suggest greater cognitive impairment.

^n^ Trail Making Test A ranges from 0 to 180. This measures the response time the participants took to draw the line as quickly as possible to connect consecutive numbers 0 to 25. Higher scores suggest greater cognitive impairment.

^o^ Trail making B ranges from 0 to 300. This measures the response time the participants took to draw the line as quickly as possible to connect numbers 0 to 13 and letters A to L in sequential order alternating between numbers and letters. Higher scores suggest greater cognitive impairment.

^p^ Digit span forward ranges from 0 to 16. The participants repeated the presented numbers in the exact order. The scores represent the number of trials participants correctly repeated the numbers. Higher scores suggest less cognitive impairment.

^q^ Digit span backward ranges from 0 to 14. The participants repeated the numbers presented in the reverse order. The scores represent the number of trials participants correctly repeated the numbers. Higher scores suggest less cognitive impairment.

^r^ Instrumental Activities of Daily Living scores range 0 to 23, higher scores indicating better functionality.

^s^ Physical activity is measured with physical activity scale for elderly. The scores range 0 to 400, higher scores indicating higher physical activity.

^t^ SPPB measures lower extremity functioning and ranges from 0 to 12. Higher score indicates better physical functionality.

^u^ Gait speed is calculated as the total distance divide by time. Higher speed indicates better physical functionality.

Thirty-four participants were randomized to arm 1 (exercise, cognitive training, and vitamin D), 35 to arm 2 (exercise, cognitive training, and placebo vitamin D), 37 to arm 3 (exercise, sham cognitive training, and vitamin D), 35 to arm 4 (exercise, sham cognitive training, and placebo vitamin D), and 34 to arm 5 (balance and toning exercise, sham cognitive training, and placebo vitamin D) (Figure 1). Overall, 31 (18%) participants withdrew during the intervention with medical complications (13 participants) unrelated to the intervention or COVID-19 restrictions (Figure 1; eTable 1 in Supplement 2). Six participants could not complete the 12-month follow-up assessments due to COVID-19 restrictions.

### Primary Outcome

In alignment with our design, a planned marginal analysis to test different orders of interactions was employed. Compared with control (arm 5) and regardless of the addition of cognitive training or vitamin D, ADAS-Cog-13 scores improved significantly in participants that received the exercise regime (arms 1 through 4: mean difference, 1.79 points; 95% CI, −3.27 to −0.31 points; *P* = .02; *d* = 0.64) (Table 2). Compared with the participants that received only the exercise regime (arms 3 and 4), those who received both exercise regime and cognitive training (arms 1 and 2) had significant ADAS-Cog-13 improvements (mean difference, −1.45 points; 95% CI, −2.70 to −0.21 points; *P* = .02; *d* = 0.39), but no significant effect of adding vitamin D to the exercise regime was found (Table 2).

**Table 2.** Effect of Exercise (Aerobic-Resistance Training) Intervention With Addition of Cognitive Training and Vitamin D at 6-Month End Point

Variable	Mean change (SE) within group^a^	Mean difference between groups (95% CI)^b^	*P* value	Effect size, *d*
**ADAS-Cog-13**				
Exercise intervention				
Arm 1 + 4 (exercise)	−1.56 (0.21)	−1.79 (−3.27 to −0.31)	.02	0.64
Arm 5 (control)	0.23 (0.68)	[Reference]		
Adding cognitive intervention				
Arm 1 + 2 (exercise, cognitive training)	−2.29 (0.45)	−1.45 (−2.70 to −0.21)	.02	0.39
Arm 3 + 4 (exercise)	−0.83 (0.45)	[Reference]		
Adding vitamin D intervention				
Arm 1 + 3 (exercise, vitamin D)	−1.40 (0.45)	0.35 (−0.93 to 1.62)	.60	0.09
Arm 2 + 4 (exercise)	−1.74 (0.47)	[Reference]		
Multidomain intervention				
Arm 1 (exercise, cognitive training, vitamin D)	−2.41 (0.62)	−2.64 (−4.42 to −0.87)	.005	0.71
Arm 5 (control)	0.23 (0.67)	[Reference]		
**ADAS-Cog-Plus**				
Exercise intervention				
Arm 1 + 4 (exercise)	−0.06 (0.04)	0.02 (−0.15 to 0.18)	.85	−0.04
Arm 5 (control)	−0.07 (0.08)	[Reference]		
Adding cognitive intervention				
Arm 1 + 2 (exercise, cognitive training)	−0.13 (0.05)	−0.14 (−0.27 to −0.003)	.048	0.34
Arm 3 + 4 (exercise)	0.01 (0.05)	[Reference]		
Adding vitamin D intervention				
Arm 1 + 3 (exercise, vitamin D)	−0.07 (0.05)	−0.03 (−0.17 to 0.11)	.69	0.07
Arm 2 + 4 (exercise)	−0.04 (0.05)	[Reference]		
Multidomain intervention				
Arm 1 (exercise, cognitive training, vitamin D)	−0.16 (0.07)	−0.09 (−0.29 to 0.11)	.39	0.21
Arm 5 (control)	−0.07 (0.08)	[Reference]		

^a^ Marginal means and standard errors obtained from linear mixed models are reported for within-group differences.

^b^ Between-group differences were assessed using the interaction between time × intervention arm. Lower scores indicate cognitive improvement.

**Figure 2.**
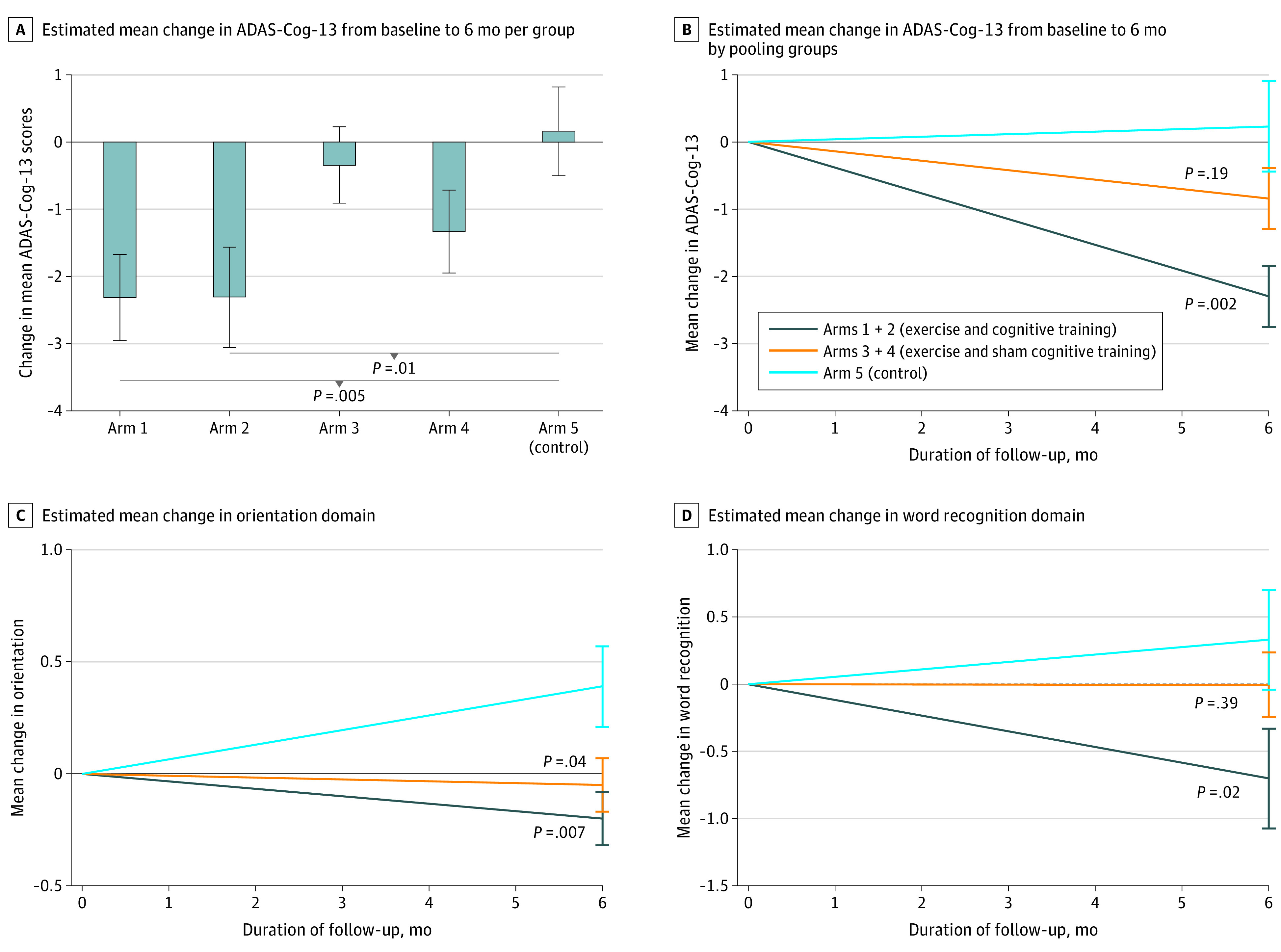
Change in ADAS-Cog-13 Scores During the 6-Month Intervention Scores above zero indicate a decline in cognitive performance; scores below zero indicate improved cognitive performance; error bars, standard errors. *P* values are compared with the control. Arm 1: aerobic-resistance exercise, cognitive training, vitamin D; arm 2: aerobic-resistance exercise, cognitive training, placebo vitamin D; arm 3: aerobic-resistance exercise, sham cognitive training, and vitamin D; arm 4: aerobic-resistance exercise, sham cognitive training, and placebo vitamin D; arm 5: balance and toning exercise, sham cognitive training, and placebo vitamin D.

The exercise regime with cognitive training (arms 1 and 2) significantly improved the ADAS-Cog-13 compared with the control (mean difference, −2.52 points; 95% CI, −4.09 to −0.94 points; *P* = .002; *d* = 0.67) (Figure 2). The difference between the exercise regime (arms 3 and 4) and the control (arm 5) on ADAS-Cog-13 was not statistically significant (Table 3). Results remained unchanged after adjustment for covariates (eTable 2 in Supplement 4).

**Table 3.** Effect of Exercise (Aerobic-Resistance Training) and Cognitive Intervention and Exercise Alone at 6-Month End Point

Variable	Within-group difference, mean (SD)^a^			Between-group difference^b^					
	Exercise and cognitive intervention (arm 1 + 2)	Exercise (arm 3 + 4)	Control (arm 5)	Exercise and cognitive intervention (arm 1 + 2) vs control (arm 5)			Exercise (arm 3 + 4) vs control (arm 5)		
				MD (95% CI)	*P* value	ES	MD (95% CI)	*P* value	ES
Primary outcome									
ADAS-Cog-13	−2.29 (0.45)	−0.84 (0.45)	0.23 (0.67)	−2.52 (−4.09 to −0.94)	.002	0.67	−1.06 (−2.64 to 0.51)	.19	0.28
ADAS-Cog-Plus	−0.13 (0.05)	0.01 (0.05)	−0.07 (0.07)	−0.05 (−0.23 to 0.12)	.56	0.10	0.09 (−0.09 to 0.26)	.34	−0.16
Individual items in ADAS-Cog									
Word Recall	−0.29 (0.14)	−0.38 (0.14)	0.02 (0.21)	−0.31 (−0.81 to 0.20)	.24	0.25	−0.40 (−0.91 to 0.10)	.12	0.34
Delayed recall	−0.46 (0.22)	−0.24 (0.22)	0.27 (0.33)	−0.73 (−1.51 to 0.05)	.07	0.39	−0.51 (−1.29 to 0.27)	.21	0.27
Following commands	0.01 (0.08)	0.10 (0.08)	0.19 (0.12)	−0.18 (−0.47 to 0.11)	.21	0.27	−0.09 (−0.38 to 0.20)	.53	0.13
Constructional praxis	0.08 (0.11)	−0.03 (0.11)	0.27 (0.17)	0.35 (−0.05 to 0.75)	.09	−0.37	0.24 (−0.16 to 0.64)	.24	−0.25
Ideational praxis	0.03 (0.05)	0.02 (0.05)	0.03 (0.08)	−0.007 (−0.19 to 0.17)	.94	0.02	−0.02 (−0.19 to 0.16)	.87	0.04
Naming objects	−0.62 (0.17)	−0.07 (0.17)	−0.10 (0.26)	−0.52 (−1.14 to 0.09)	.10	0.36	0.03 (−0.58 to 0.64)	.92	−0.02
Orientation	−0.20 (0.12)	−0.05 (0.12)	0.39 (0.18)	−0.59 (−1.01 to −0.17)	.007	0.58	−0.44 (−0.86 to −0.02)	.04	0.44
Word recognition	−0.70 (0.25)	−0.05 (0.24)	0.33 (0.37)	−1.03 (−1.90 to −0.16)	.02	0.50	−0.39 (−1.25 to 0.48)	.39	0.19
Remembering instructions	−0.005 (0.05)	−0.05 (0.05)	−0.05 (0.08)	0.04 (−0.14 to 0.22)	.66	−0.10	−0.009 (−0.19 to 0.17)	.92	0.02
Comprehension	−0.01 (0.06)	−0.03 (0.06)	0.03 (0.09)	−0.04 (−0.25 to 0.17)	.69	0.09	−0.06 (−0.27 to 0.15)	.58	0.12
Word finding	0.03 (0.07)	−0.07 (0.07)	−0.09 (0.11)	0.11 (−0.15 to 0.37)	.40	−0.18	0.02 (−0.24 to 0.28)	.90	−0.03
Spoken language	−0.02 (0.06)	−0.13 (0.06)	−0.12 (0.09)	0.10 (−0.10 to 0.30)	.35	−0.20	−0.006 (−0.21 to 0.20)	.96	0.01
Number cancellation	−0.14 (0.10)	0.13 (0.10)	−0.14 (0.16)	−0.003 (−0.38 to 0.37)	.99	0.003	0.27 (−0.11 to 0.65)	.16	0.30
Trail making A	−3.22 (1.93)	−1.21 (1.95)	5.26 (2.99)	−8.48 (−15.45 to −1.52)	.02	0.52	−6.47 (−13.45 to 0.51)	.07	0.39
Trail making B	7.63 (6.8)	−5.92 (6.8)	5.17 (10.6)	2.46 (−22.2 to 27.13)	.85	−0.04	−11.10 (−35.76 to 13.57)	.38	0.19
Digit symbol substitution	1.40 (1.10)	0.41 (1.16)	−1.29 (1.70)	2.69 (−1.29 to 6.66)	.19	0.29	1.70 (−2.33 to 5.74)	.41	0.18
Digit span forward	−1.20 (0.40)	−1.21 (0.40)	−0.83 (0.60)	−0.37 (−01.78 to 1.04)	.61	0.11	−0.38 (−1.79 to 1.03)	.60	0.11
Digit span backward	−1.22 (0.31)	−0.82 (0.31)	−0.67 (0.46)	−0.56 (−1.65 to 0.53)	.32	0.22	−0.16 (−1.25 to 0.93)	.78	0.06
Category fluency animals	0.80 (0.50)	−0.37 (0.50)	1.63 (0.75)	−0.83 (−2.59 to 0.93)	.36	0.20	−2.00 (−3.76 to −0.24)	.03	0.48
Category fluency vegetables	0.08 (0.44)	−0.68 (0.44)	1.11 (0.66)	−1.03 (−2.58 to 0.52)	.20	0.28	−1.79 (−3.34 to −0.25)	.02	0.49

Abbreviations: ES, effect size; MD, mean difference.

^a^ Marginal means and standard errors obtained from linear mixed models are reported for within-group differences.

^b^ Between-group differences were assessed using the interaction between time × intervention arm. Lower scores indicate cognitive improvement.

Inside the table analyses showed a significant ADAS-Cog-13 improvement in arm 1 (exercise, cognitive training, and vitamin D: mean change, −2.64 points; 95% CI, −4.42 to −0.87 points; *P* = .005; *d* = 0.71) and arm 2 (exercise, cognitive training, and placebo vitamin D: mean change, −2.39 points; 95% CI, −4.20 to −0.57 points; *P* = .01; *d* = 0.63) compared with control (arm 5; eTable 3 in Supplement 4). The estimated mean (SE) change from baseline to post-intervention was −2.41 (0.67) in arm 1, −2.16 (0.65) in arm 2, and 0.23 (0.67) in arm 5 (control). There were no significant improvements in ADAS-Cog-13 scores from baseline in arm 3 (exercise, sham cognitive training, and vitamin D) and arm 4 (exercise, sham cognitive training, and placebo vitamin D) compared with control. Adjusting for covariates did not change the results (eTable 4 in Supplement 2).

Clinically significant improvement in ADAS-Cog-13 (3 points or more) was observed for 44% of participants in arm 1, 37% in arm 2, 14% in arm 3, 24% in arm 4, and 15% in arm 5 (eTable 5 in Supplement 2). Per-protocol analysis did not alter our results (eTables 6 through 8 in Supplement 2).

No significant improvement in the ADAS-Cog-Plus scores across the intervention arms compared with the control (arm 5) was identified (Table 2 and 3). Post hoc analyses of the ADAS-Cog-Plus without the verbal fluency test (keeping the 4 executive function tests that create the ADAS-Cog-Plus variant) revealed a significant improvement for arm 1, the multidomain intervention (eTables 9 and 10 in Supplement 2).

### Follow-Up Analyses

ADAS-Cog-13 scores improved from baseline to 12-month follow-up across all intervention arms. The improvements observed at postintervention remained during follow-up for arm 2 (exercise, cognitive training, and placebo vitamin D) and arm 3 (exercise, sham cognitive training, and vitamin D), but were slightly deteriorated in arm 1 (exercise, cognitive training, and vitamin D) and arm 4 (exercise, sham cognitive training, and placebo vitamin D), although none of the follow-up analyses were statistically significant (eTables 11 and 12, eFigures 1 and 2 in Supplement 2). Exercises and physical activity levels were not maintained during the 6-month follow-up (eTable 13 in Supplement 2).

### Secondary Outcomes

The interventions effect on individual ADAS-Cog items are shown in Tables 2 and 3. A significant improvement was observed for delayed recall (mean difference: arm 2 vs 5, −0.99 points; 95% CI, −1.99 to −0.09 points), orientation (arm 1 vs 5, −0.57 points; 95% CI, −1.05 to −0.10 points; arm 2 vs 5, −0.61 points; 95% CI, −1.09 to −0.13 points), word recognition (arm 1 vs 5, −1.33 points; 95% CI, −2.32 to −0.36 points), and TMT-A (arm 1 vs 5, −11.25 points; 95% CI, −19.02 to −3.49 points).

### Adherence and Adverse Events

There were no group differences in the median training duration. Adherence to exercise regimes, cognitive training, and their respective control conditions, were equivalent across the 5 arms (87%). A total of 52 adverse events were reported (2 minor strokes, 1 hip fracture, 24 musculoskeletal pain, 6 falls, 4 numbness, 3 dizziness, and 11 others) but they were not intervention related (eTable 14 in Supplement 2).

## Discussion

The primary objective of the SYNERGIC Trial was to evaluate the effect of a multidomain intervention (combination of progressive exercise with cognitive training and vitamin D supplementation) on cognition compared with both an active control and exercise alone in older adults with MCI. Exercise with cognitive training significantly improved ADAS-Cog-13 scores, driven by improvements in episodic memory, attention, and orientation. Exercise alone did not improve cognition, nor did adding vitamin D. ADAS-Cog-Plus did not improve with any of the interventions. Compared with our multidomain interventions, the control arm revealed a slight decline in cognition, suggesting that balance and toning exercises may not sustain cognitive function in MCI individuals. Cognitive improvements observed in the ADAS-Cog-13 immediately after the intervention were slightly attenuated at 12-month assessment but they did not revert to baseline scores, suggesting a lasting effect even without participants engaging in exercise regimes during the follow-up period. Finally, our multidomain intervention achieved high compliance and adherence, and was safe and feasible to perform in older adults with MCI.

A 2.64-point improvement in the ADAS-Cog-13 for the multidomain intervention is larger than changes seen in previous pharmaceutical trials among individuals with MCI or mild dementia,^32^ and approaches the 3 points considered clinically meaningful.^33^ Together with the moderate-to-large effect size (0.71) found, our results support a beneficial cognitive effect from this multidomain intervention.

The lack of significant effect on the ADAS-Cog-Plus was against our hypotheses. Changes for arms 1 and 2 (exercise plus cognitive training) were in the expected direction, which prompted us to conduct exploratory analyses to evaluate whether adding items to the ADAS-Cog-13 may have reduced its responsiveness to our interventions. These post hoc analyses revealed that the ADAS-Cog-Plus variant without the verbal category fluency item (just adding 4 out of 5 plus items to the ADAS-Cog-13) was sensitive in detecting significant improvements for our multidomain intervention. Adding items to a composite measure would increase its sensitivity only to the extent that it may improve the mean-to-SD ratio of change over the course of the study. In other words, the optimal composite maximizes the signal and minimizes the noise. Adding the category fluency may have reduced the responsiveness of the Plus variant to our intervention because exercise and dual-task cognitive training interventions typically show greatest effects on measures of attention and pure executive function rather than on verbal fluency.^24,35^

Our results align with a 2022 meta-analysis^14^ showing a significant effect of multidomain interventions in MCI for improving global cognition, executive function, and episodic memory. Interestingly, only 7 of the 28 trials included in the meta-analysis combined aerobic-resistance exercise with cognitive training and none of them showed that the multidomain intervention has a larger effect than exercise alone. Our trial is to our knowledge the first to show a greater effect of a multidomain intervention over exercise alone. The lack of cognitive improvements in previous MCI multidomain interventional trials could be related to the substantial heterogeneity among intervention protocols, as well as a lack of meaningful exercise progression.

The lack of improvement with vitamin D may be related to normal-high serum values (greater than 70 nmol/L) in our participants at enrollment, whereas benefits of vitamin D supplementation may only occur in the presence of severe 25-hydroxyvitamin D deficiency (below 30 nmol/L).^35^ We could not perform a subanalysis by deficiency status as only 4 participants were severely deficient, but this should be a target for future research since vitamin D deficiency is associated with impaired executive function and progression to dementia.^34^

Exercise training cessation may induce detraining effects,^36^ but maintenance of cognitive improvements was reported in short follow-ups.^37^ In this trial, ADAS-Cog-13 improvements diminished slightly at 12-month follow-up but did not revert to baseline levels for arms 1, 2, and 3. Such findings suggest potential maintenance of the cognitive improvements up to 6-month postintervention for exercise with cognitive training or vitamin D.

### Strengths and Limitations

Strengths of our trial included the selection of a fractional factorial design to test the sparsity-of-effects principle and, therefore, expose interaction effects over low-order interactions, such as the effect of vitamin D. Additional strengths were the use of a primary outcome sensitive to cognitive changes in MCI,^23^ the systematic progression of exercise and cognitive training, and targeting a population considered to be at the ideal intervention stage; this may explain the large effect size found when comparing our intervention with previous multidomain trials not targeting MCI.^10,12^

Our trial had several limitations. The early termination imposed by COVID-19 pandemic restrictions impeded us from reaching our target sample size and provoked dropouts in those already enrolled, which likely affected our power to find a statically significant effect, for instance, for the exercise intervention alone. Most participants were vitamin D sufficient at baseline, which may have reduced any potential effect of the vitamin D intervention. Additionally, our sample was largely White and with postsecondary education, which reduces the generalizability of our results. Finally, we did not adjust our analyses for having 2 primary end points, which may have increased our type I error.

## Conclusions

The SYNERGIC Trial demonstrated that a multidomain intervention of progressive aerobic-resistance exercises with sequential cognitive training can improve global cognition, including memory, attention, word recognition, and orientation in older adults with MCI, as measured by one of our primary outcomes, the ADAS-Cog-13. The multidomain intervention effect was larger than the improvement from exercise alone. Vitamin D supplementation had no significant benefit. Our findings suggest that this multidomain intervention could induce a clinically meaningful cognitive improvement in individuals with MCI, which may have important implications for their quality of life.

## Notes

Supplement 1.
Trial Protocol, Statistical Analysis Plan, and Trial Protocol Amendments

Supplement 2.
eMethods.
**eTable 1.** Details of the Participants Who Were Withdrawn From the SYNERGIC Trial Due to Medical Reasons **eTable 2.** The Effect of Exercise, With (Arm 1+2) and Without (Arm 3+4) Cognitive Intervention on ADAS-Cog 13 Scores at 6-Month End Point, Compared With Control (Arm 5), and Adjusted for Age, Sex, Education, and Comorbidities **eTable 3.** Within-Group and Between-Group Intervention Effect Across 5 Intervention Arms at 6-Month End Point **eTable 4.** Within-Group and Between-Group Intervention Effect Across 5 Intervention Arms at 6-Month End Point, Adjusted for Age, Sex, Education, and Comorbidities **eTable 5.** Number of Participants With Improvement in ADAS-Cog-13 and Plus Variant at 6 Months by Intervention Arm **eTable 6.** Per-Protocol Analysis of the Effect of Within-Group and Between-Group Intervention Across 5 Intervention Arms at 6-Month End Point **eTable 7.** Per-Protocol Analysis for the Effect of Exercise Intervention With Addition of Cognitive Training and Vitamin D at 6-Month End Point **eTable 8.** Per-Protocol Analysis for the Effect of Exercise and Cognitive Intervention and Exercise Alone at 6-Month End Point **eTable 9.** The Effect of Intervention on ADAS-Cog-Plus Scores at 6-Month End Point Calculated by Cumulatively Adding Individual Plus Items **eTable 10.** The Effect of Exercise, With (Arm 1+2) and Without (Arm 3+4) Cognitive Intervention on ADAS-Cog-Plus Scores at 6-Month End Point Compared With Control (Arm 5), Calculated by Cumulatively Adding Individual Plus Items **eTable 11.** Change in ADAS-Cog-13 and ADAS-Cog-Plus at 12-Month Follow-up **eFigure 1.** Change in ADAS-Cog-13 Scores From Baseline to 12 Months Across 5 Arms **eTable 12.** The Effect of Exercise, With (Arm 1+2) and Without (Arm 3+4) Cognitive Intervention at 12-Month Follow-up, Compared With Control (Arm 5) Using “At the Margin” Analysis **eFigure 2.** Change in ADAS-Cog-13 Scores From Baseline to 12 Months **eTable 13.** Exercise and Physical Activity Level Reported at Month 12 **eTable 14.** Self-Reported Adverse Events and Health Care Follow-up Diagnosis During the Study
eReferences.

Supplement 3.
Nonauthor Collaborators

Supplement 4.
Data Sharing Statement
