# Supplementary material for: Effects of Exercise Alone or Combined With Cognitive Training and Vitamin D Supplementation to Improve Cognition in Adults With Mild Cognitive Impairment: A Randomized Clinical Trial
Source: JAMA Netw Open. 2023 Jul 20;6(7):e2324465. doi: 10.1001/jamanetworkopen.2023.24465 (PMC10359965; doi:10.1001/jamanetworkopen.2023.24465)
Supplement: Supplement 1. — Trial Protocol, Statistical Analysis Plan, and Trial Protocol Amendments [file jamanetwopen-e2324465-s001.pdf]

SYNchronizing, **E**xercises, **R**emedies in **G**a**I**t and **C**ognition (SYNERGIC). A randomized controlled double blind trial.

CCNA-2019
The Canadian Consortium for Neurodegeneration in Aging
15-February-2019

HC6-24-c195918 Protocol #201619
NCT02808676

****Confidentially Statement***
The information contained in this document is privileged and confidential. Any disclosure is prohibited unless such disclosure is required by applicable laws and regulations. Persons to whom the information is disclosed must be informed that the information is privileged and confidential and it may not be further disclosed by them.

SYNERGIC TRIAL (**SYN**chronizing **E**xercises, **R**emedies in **G**a**I**t and **C**ognition)
A Randomized Controlled Double Blind Trial

Table of Contents

1. INTRODUCTION......6
1.1 Background......6
2. STUDY DESIGN and HYPOTHESES......7
2.1. HYPOTHESES......7
3. DESIGN......8
4. STUDY OUTCOMES......9
4.1 Primary Outcome:......9
4.2 Secondary Outcomes......9
5. STUDY POPULATION......10
5.1 Participants, recruitment, stratification, and blinding......10
5.2 Inclusion and Exclusion criteria......10
Inclusion criteria:......10
Exclusion criteria:......11
5.3 Sample size:......12
6. STUDY PROCESS......12
6.1 Interventions......12
6.1.1 Cognitive Training (Multimodal CT-intervention)......13
6.1.2 Exercises (Multimodal Exercise – Intervention)......14
6.1.3 Vitamin D supplementation......15
6.2 Control/Placebos......16
7.0 STUDY PROCEDURES......17
7.1 Screening Session (Visit 1)......18
7.2 Baseline Evaluation (Visit 2)......19
7.2.1 Treatment Allocation......19
7.2.2 Blinding......20
7.3 Visit 3 – 6 month follow-up visit......20
7.4 Telephone Follow-Up (9 month post-baseline testing)......20
7.5 Visit 4 – 12 month follow-up visit......21
7.6 Study Procedure Descriptions......21

7.6.5 Cognitive Outcomes........22
7.6.6 Mobility Outcomes........25
7.6.7 Functional Outcomes and Combined Score of Cognition and Functionality (Pooled Index)........27
7.6.8 Quality Of Life Outcomes........28
7.6.9 Health Resource Utilization........28
8. ANALYSIS........29
Analysis, planned analysis, frequency of analysis, and correction for multiple testing........29
9. ADVERSE EVENT (AE) AND SERIOUS ADVERSE EVENT REPORTING (SAE)........30
9.1 Concomitant Medications........30
9.2 Rescue Medication and Risk Management........31
9.3 Premature Withdrawal/Discontinuation Criteria........31
10. ETHICAL CONSIDERATIONS........31
10.1 Confidentiality of Participant Records........32
11. ADMINISTRATIVE REQUIREMENTS........32
11.1 Protocol Amendments........32
11.2 Completion of Case Report Forms (CRFs)........32
11.3 Monitoring Procedures........32
12. PUBLICATION POLICY........32
12.1 Participating Centers........32
AppendIX I........42

LIST OF ABBREVIATIONS AND DEFINITIONS OF TERMS

Abbreviations	
ADAS-Cog	**Alzheimer's Disease Assessment Scale Cognition Plus**
ADCS	**Alzheimer's Disease Cooperative Study**
ADL	**Activities of Daily Living**
ADNI	**The Alzheimer's Disease Neuroimaging Initiative**
ANOVA	**Analysis of Variance**
BDNF	**Brain-Derived Neurotrophic Factor**
BNT	**Boston Naming Test**
CCNA	**Consortium in Neurodegeneration and Aging**
CDR	**Clinical Dementia Rating Scale**
cE	**Control Tone-Stretching Exercises**
CERAD	**Consortium to Establish a Registry for Alzheimer's Disease**
CT	**Cognitive Training**
cCT	**Control Cognitive Training**
DM	**2 Tasks at Once**
E	**Multi-Modal Exercises**
FLAIR	**Fluid Attenuation Inversion Recovery**
fMRI	**Functional MRI**
GAD-7	**Generalized Anxiety Disorder 7-item scale**
GDS-30	**Geriatric Depression Scale**
IADL	**Instrumental Activities of Daily Living**
ICH-GCP	**Harmonization Good Clinical Practice**
IRB	**Institutional Review Board**
IU	**International units**
K-M	**Kaplan-Meier**
LORIS	**Longitudinal Online Research and Imaging System**
MCI	**Mild Cognitive Impairment**
MMSE	**Mini Mental State Examination**
MoCA	**Montreal Cognitive Assessment**
MRI	**Magnetic Resonance Imaging**
PARQ+	**Physical Activity Readiness Questionnaire Plus**
PASE	**Physical Activity Scale for the Elderly**
PRT	**Progressive resistance training**
PTH	**Parathyroid hormone**
RAVLT	**Rey auditory verbal learning**
RPE	**rate of perceived exertion**
RT	**Reaction time**
SYNERGIC	**SYNchronizing Exercises, and Remedies in GaIt and Cognition**
SDST	**Symbol digit substitution test**
SF-36	**Quality of life questionnaire – short form**
SPPB	**Short physical performance battery**

TMT	**Trail making test**
VBM	**Voxel-Based Morphometry**
WAIS	**Wechsler Abbreviated Scale of Intelligence**
WMHs	**White Matter Hyper-intensities**
VEGF	**Vascular endothelial growth factor**
6MWT	**Six Minute Walk Test**

1. INTRODUCTION

1.1 BACKGROUND

Exercises, specifically resistance and aerobic training, have been demonstrated to improve cognitive outcomes, along with improved physical capacity and mobility[1,2]. Both aerobic [3] and resistance training[4] trials of different durations have revealed impressive results, with the most consistent findings being observed after combined interventions, multimodal exercise- lasting roughly six months to one year[1]. For example, our group has recently reported that aerobic exercise improves performance on learning a new sequence of movements. Although the training benefits of progressive resistance training (PRT) have been well documented, PRT has been studied far less extensively than aerobic training in patients with Mild Cognitive Impairment (MCI).

Exercise training is beneficial for cognition even in frail older adults and in those with low mobility as has been shown by our group[4]. Exact mechanisms supporting the benefits of exercises for cognition in humans need to be further explored but numerous studies in animals and humans have demonstrated that aerobic exercise may have neuroprotective and neurorestorative effects [4,5,5,6]. The rationale of combining aerobic and progressive resistance training as a multimodal exercise intervention is supported by research that has revealed potential beneficial effects on insulin-like growth factor-1, insulin sensitivity, and anti-inflammatory and brain-derived neurotrophic factor pathways, which are related to both sarcopenia and cognitive decline[7]. In addition, multimodal exercise interventions have shown positive effects on muscle/lean mass, cognition and brain volume [8].

Similarly, cognitive training (e.g. computer based cognitive process training) has also shown positive results in improving cognition, mobility and postural control. Several recent systematic reviews on the topic[9,10] support the cognitive benefits of training and a seated dual-task cognitive training regimen designed by our group has demonstrated that this type of training can also improve balance in healthy older adults.[11] In line with exercise training, recent research on cognitive training has also supported important improvements in brain plasticity post-intervention [12,13].

In addition, vitamin D deficiency has been linked to cognitive dysfunction, dementia and mobility decline. [1,14-16] Besides its very well-known effects on muscle and bone physiology, vitamin D is considered a neurosteroid hormone which exhibits neuroprotective attributes through antioxidative mechanisms, neuronal calcium regulation, immunomodulation, enhanced nerve conduction and detoxification mechanisms.[14,17-20] Compelling evidence from animal models and epidemiological studies supports a potential beneficial role for vitamin D on cognitive function. In this trial, we will test

if vitamin D supplementation will have a synergistic effect in improving or stabilizing cognitive function in combination with exercises and cognitive training.

Mild Cognitive Impairment is thought to be the optimum stage to intervene with preventive strategies and early treatments, in fact it is estimated that one-year delay in dementia incidence could save $109 billion over a 30 years period the Canadian Health System.[21,22] Robustly designed trials, with longitudinal follow-up, have been recommended in MCI to investigate the comparative benefits of isolated and combined physical exercises and cognitive training. To date, the effect of adding cognitive training and vitamin D to multimodal physical exercises for improving global cognition, executive function and memory in MCI has not been assessed.[23]

The proposed **SYNERGIC TRIAL (SYN**chronizing **E**xercises, and **R**emedies in **G**a**I**t and **C**ognition) is uniquely designed to evaluate the effect of aerobic and progressive resistance training exercises, isolated or combined with cognitive training and vitamin D supplementation, in older adults with MCI. This trial will be conducted by the Motor Exercise and Cognition Team which is part of the Canadian Consortium in Neurodegeneration and Aging (CCNA) and the Canadian Gait and Cognition Network (CGCN).

2. STUDY DESIGN AND HYPOTHESES

2.1. HYPOTHESES

2.1.a Primary Hypotheses

1. Twenty weeks (five months) of supervised multimodal exercise (aerobic and progressive resistance training) alone and/or in combination with cognitive training (CT) and/or oral vitamin D will significantly improve global cognitive function in older adults with MCI, as assessed by primary outcome **Alzheimer's Disease Assessment Scale-Cognitive subscale plus (ADAS-Cog Plus).**
2. Multimodal exercise alone and/or in combination with CT and/or oral vitamin D would significantly improve secondary cognitive outcomes across memory, executive function, and attention, as well mobility outcomes related to falls risk and dual-task gait, and dual-task gait cost.

3. The combination of CT and oral vitamin D with multimodal exercise will be significantly superior to either intervention alone for cognitive and functional benefits, demonstrating a synergistic effect.
4. Mechanistically, our intervention will significantly improve hippocampi volume, blood biomarkers, and BDNF levels [24], and fMRI prefrontal activation in executive control dual-task condition.

2.1.b Secondary Hypotheses

1. All active training interventions will improve brain morphology and biochemistry compared with control –placebo-interventions as defined by: increased hippocampal volume (mm^3) by MRI scanning; positive localized Voxel-Based Morphometry (VBM) brain changes (z-score relative change); decreased whole brain volume of White Matter Hyper-intensities (WMHs) (mm^3); and lead to beneficial hippocampal and anterior and posterior cingulate MRS metabolite changes (% increase in N-acetylaspartate, and increase in phosphocreatine metabolites).
2. All active interventions will improve secondary cognitive outcomes, in the domains of attention, memory, fluency, and executive function, relative to the control condition, and combined training will be superior to either single intervention.
3. Multimodal exercise will preferentially decrease inflammatory markers, and increase fitness (strength and aerobic capacity), muscle strength, and functional mobility, compared to either cognitive or control condition.
4. Cognitive and physical training will produce positive effects on psychological health and quality of life (SF-36) above and beyond the non-specific effects seen after control condition.

3. DESIGN

The **SYNERGIC TRIAL (SYN**chronizing **E**xercises, and **R**emedies in **G**a**I**t and **C**ognition) is a Randomized, modified factorial design with parallel-groups (five arms), and double-blind study **evaluating the cognitive effect of multimodal exercises (as a main intervention) combined with and without cognitive training and vitamin D**. This is achieved using 4 arms (1 to 4) and a 5th arm is added as a control group. Figure 1 illustrates the trial design and Figure 2 summarizes the timeline of the trial consisting of an approximate 12-18 months enrolment period, and 12 months of follow-up. The trial adheres to the Consolidated Standards of Reporting Trials (CONSORT) guidelines for conducting and reporting clinical trials, as extended to non-pharmacologic interventions. Two hundred (200)

participants with Mild Cognitive Impairment (MCI), aged 60 and older will be enrolled and randomized into one of five arms.

Arm 1: **multimodal exercises + cognitive training + Vitamin D**
Arm 2: **multimodal exercises + cognitive training** + placebo D
Arm 3: **multimodal exercises**+ control cognitive training + **Vitamin D**
Arm 4: **multimodal exercises** + control cognitive training+ placebo D;
Arm 5: tone-stretching exercises + control cognitive training+ placebo D.

Note: in **bold** are the active interventions. Arm 5 is the arm with pure control interventions.
Figure 1 shows the flow chart of the trial. **E**: multimodal exercises; **CT:** cognitive training; **cE**: control tone-stretching exercises; and **cCT:** control cognitive training.

4. STUDY OUTCOMES

4.1 PRIMARY OUTCOME: Global cognition assessed using the ADAS-Cog Plus.[25]
We expect that our combined interventions, over 20 weeks, will show improvement and/or less decline in **global cognition.** We expect a three (3) point change (continuous variable) across full intervention/ control groups on the ADAS-Cog Plus scores, a value we considered clinically significant based in previous studies using the ADAS-Cog 13 items. This expected change/ improvement is similar in magnitude to the improvements seen in positive pharmacological clinical trials in dementia in the ADAS-Cog .[26] Additionally, improving in the proportion of participants, expressed as percentages per allocated groups, with abnormal ADAS-Cog Plus (dichotomous variable) after intervention will be considered success of the intervention. ADAS-Cog Plus is described in detail in Section 7. We also expect a significant statistical improvement in the overall transformed score of ADAS-Cog Plus calculated using a validated scoring system[27] Observing a statistically significant difference in this primary outcome will be considered preliminary evidence of efficacy.

4.2 SECONDARY OUTCOMES
Secondary outcomes include neuroimaging, cognitive (neuropsychological assessments), gait and mobility outcomes including incidence of fall, blood biomarkers, quality of life scorings, as described in our primary and secondary hypotheses. Secondary outcomes are described in detail in Section 7.

5. STUDY POPULATION

5.1 PARTICIPANTS, RECRUITMENT, STRATIFICATION, AND BLINDING

We will target older adults aged 60 and over with MCI. Interventions will be done across five sites: London (lead and sponsor site, target recruitment: 70 participants), Waterloo-University of Waterloo (target recruitment: 25), Waterloo-Wilfrid Laurier University (target recruitment: 20), Montreal (target recruitment: 30) and Vancouver (target recruitment: 55). Each intervention site may have more than one recruitment center. For those who are interested in participating, a consent and screening session will be arranged as described in our participant flowchart, Figure 3.

Once informed consent is obtained, we will perform the screening tests. Those who remain eligible after the screening session including the Physical Activity Readiness Questionnaire Plus (PARQ+) and who provide written recommendation from their physician or site physician indicating their appropriateness to participate in an aerobic-based exercise training program will proceed to baseline assessments. Participants will be informed that they will be randomly assigned to one of five arms by the research coordinator and will be blinded to the investigators' hypothesis as to which is the preferred intervention arm.

All groups will have an equal volume and frequency of contact with trainers over the 12 months of the study. All primary and secondary outcomes will be obtained and analyzed by blinded assessors on different days to the training programs.

5.2 INCLUSION AND EXCLUSION CRITERIA

Inclusion criteria:

Participants must meet each of the following criteria for enrolment into the study:

1. At least 60 years of age.
2. Self-reported levels of proficiency in English or French (at Montreal site only) for speaking and understanding spoken language.
3. Able to comply with scheduled visits, treatment plan, and other trial procedures.
4. Able to ambulate at least 10 m independently.
5. Having MCI defined following Albert et al.[28] criteria as:
   a-Objective cognitive impairment in one of the following four cognitive domains: memory, executive function, attention, and language operationalized using one or more of the following: MoCA Test (Montreal Cognitive Assessment) with scores ranging from 13-24/30, Logical Memory below ADNI cutoffs, CERAD word list recall < 6.

b-Preserved activities of daily living operationalized as a Score >14/23 on the Lawton-Brody IADL[29] scale and confirmed by clinician's interviews.

c-Absence of dementia using criteria from the Diagnostic and Statistical Manual of Mental Disorders, Fifth Edition [30] and/or Global CDR = 0.5.

6. Having normal or corrected to normal vision in at least one eye so that they can identify symbols and stimuli presented on a computer screen in front of them.
7. Must be in sufficient health to participate in the study's aerobic-based exercise training program. This will be determined using the PARQ+ screening tool and with an evaluation by a certified exercise physiologist and/or a physician, when appropriate indicating clearance to participate in an aerobic-based exercise training program.

Exclusion criteria:

Participants who exhibit any of the following conditions are to be excluded from the study:

1. Serious underlying disease which, in the opinion of the investigator, may preclude engagement in interventions or may interfere with the participant's ability to participate fully in the study.
2. Participant with uncontrolled major depression, schizophrenia, severe anxiety and drug abuse.
3. Current parkinsonism or any neurological disorder with residual motor deficits (e.g. stroke with motor deficit), active musculoskeletal disorders (e.g. severe osteoarthritis of lower limbs) or history of knee/hip replacement affecting gait performance at clinical evaluation
4. Intention to enroll in other clinical trials during the same time period.
5. Pre-existing or current active participation in physical activity program involving aerobic or resistance training regimen in previous 6 months.
6. Taking Cognitive enhancers, Neuroleptics, Anticholinergics, or Vitamin D in doses more than 1000IU/day or equivalent.
7. Known hypercalcemia and/or disorder that is cause of hypercalcemia (e.g. hyperparathyroidism/ Paget's disease/ Sarcoidosis).
8. Known renal/kidney insufficiency.
9. Uncontrolled hypertension.
10. Uncontrolled diabetes.

5.3 SAMPLE SIZE:

Sample size calculation is based on changes in our main primary outcome: stabilization/improvement in cognition as measured using the **Alzheimer Disease Assessment Scale Cognitive Plus (**ADAS-Cog Plus**)**. We expect a change of three (3) points on the ADAS-Cog Plus scores, a value that is considered clinically significant, and it is similar in magnitude to the improvements seen in positive pharmacological clinical trials in dementia.[26] Two previous studies testing the effect of exercises on cognition in 200 MCI participants shown a mean score of 6-7 and a standard deviation of 3 in the ADAS-Cog.[24,31] Based on these data and assuming a mean change of 3 points in the ADAS-Cog, 34 participants per arm are required to have 80% power to detect a change of three points or more on the ADAS-Cog with a 2-tailed α error of 5%, adjusted for 10 potential pairwise comparisons. We estimate a worst case scenario of 15% of dropout rate during the 20 weeks of follow up to the main endpoint. Therefore, we will aim to recruit 40 per arm for a total of 200 participants.

Sample size estimates per group for the primary outcome: change in ADAS-Cog Plus

	Type I error=0.05	Type I error=0.01	Type I error=0.001
Power=80%	34	50	59
Power=90%	51	54	70
Power=99%	68	82	102

Reference: Independent Groups - Machin D, Campbell M, Fayers, P, Pinol A (1997) Sample Size Tables for Clinical Studies Second Ed. Blackwell Science IBSN 0-86542-870-0 p. 24-25

6. STUDY PROCESS

6.1 INTERVENTIONS

All participants will complete three (3) group training sessions per week (total 20 weeks), under the supervision of trainers. Each session will last approximately 90 minutes and will be comprised of 30 minutes cognitive training (CT) or control cognitive training (cCT) followed by approximately 60 minutes of combined aerobic and progressive strengthening exercise (E) or control tone-stretching exercise (cE) and. All participants will receive a vitamin D (tablet 10,000 IU) or placebo three times per week.

Within each small group of up to eight individuals, participants follow the program tailored to their individual functioning level, with constant monitoring by the trainers. Participants are expected to attend all training sessions and research staff will strongly encourage them to do so. Each training group will have 1-2 trainers present each session to ensure a ratio of one (1) trainer per four (4) participants. To

avoid potential imbalances in exposure time, control interventions for exercise and cognitive training will have the same duration.

Throughout the 12 month trial, participants will be provided with falls calendars, in which they will record any falls that have occurred, and they will be asked to bring them at the training sessions. At the completion of the 20-week intervention, participants are not given ongoing access to the training. During follow-up, participants will be called three months after their completion of the intervention to track incidence of falls, adverse outcomes, such as emergency room visits and change of medications; physical activity will be also be tracked using the Physical Activity Scale for the Elderly (PASE). Following assessments at 6 and 12 months, participants will receive a compensation of $50 for their participation for a total of $100.

6.1.1 COGNITIVE TRAINING (MULTIMODAL CT-INTERVENTION)

Cognitive Training (CT) intervention will involve tablet-based multimodal and multi-domain dual-task training with memory load. The training uses the custom-written program, developed for neuro-rehabilitation and used in previous research trials for cognitive [32-34] and mobility outcomes [11]. Training sessions will take place in groups of up to eight participants before each of the fitness-training session for duration of 30 min max. Participants will perform a concurrent visuo-motor task (dual-task combination) composed of different sets of visual stimuli that have to be identified by tapping designated figures on an iPad (or an Android tablet). Participants will perform discrimination tasks involving sets of items (e.g. letters, numbers, animals, vehicles, fruits, celestial bodies). Each session will involve two sets of items, one for each task. The combination of sets of items will change every four sessions.

Task instructions will always be administered by a trained Research Assistant. Within each training session and for each task combination, participants will complete a first block of single-pure (SP) trials followed by mixed-trial blocks with single-task trials (SM) intermixed and dual-mixed trials (DM: 2 tasks at once). Each training session will involve 80 SP, 128 SM and 192 DM trials (thus 40% of the DM blocks will be SM trials). Within the DM trials, the first 30 training sessions will be performed following a fixed priority instruction (participants will be asked to keep an equal priority on left and right hands.) The following 30 sessions will be performed following a variable priority instruction (participants will be told to vary priority from one hand to another in different blocks). Participants will complete a total of 60 sessions. Importantly, an adaptive continuous feedback on performance was will be provided during the dual-mixed block of the training sessions.

Feedback will take the shape of a speedometer whose indicator moves and changes colour (green, yellow, orange or red) to inform participants of their response speed. Two speedometers (one for each hand) will be displayed at the top of the screen. Each speedometer is associated to one task. In equal priority blocks, participants will be asked to try to maintain both speedometers at least in the yellow zone and prevent them from turning to red. In variable priority blocks, participants will be asked to maintain the speedometer associated to the prioritized task in the green zone, while the other speedometer can be at least in the orange zone. The colour of the speedometers will be determined by the average reaction time (RT) on the last three trials' RT for the DM block compared to the median RT for the SM trials multiplied by a factor of 1.5 (for the first training session, as there are no previous SM trials, the DM block will be compared to 1500ms). In addition, a feedback will be provided at the end of the session, where participants will be informed of mean RT and accuracy achieved throughout sessions (presented in a histogram without explicit values).

6.1.2 EXERCISES (MULTIMODAL EXERCISE – INTERVENTION)

The exercises training program involves 3 weekly sessions of approximately 60 min each. All sessions will be held in appropriate gym facilities and take place between Monday and Friday ensuring that there are not three consecutive days (i.e. training can occur on Monday, Wednesday and Friday or Monday, Thursday and Friday etc.). Staff with experience and/or certification in exercises (graduate student in kinesiology, Canadian Society of Exercise Physiology certificates, etc.) will supervise all training sessions with at least one coach per four participants.

After a general warm-up, participants will execute the strength-training portion by performing light strengthening exercises (pushes and pulls using elastic bands, chair stands, and forward lunges). The next phase will include the following exercises: leg press, leg flexion (lower body), seated press, seated row, and seated pull (upper body). These exercises are made in a circuit alternating between lower and upper body exercises. A list of suggested exercises base on muscle groups will be provided to the trainers (see appendix II). The recovery period between exercises corresponds to the time needed to move from one station to another. While the first portion of this training protocol (12 weeks) will be dedicated to strength endurance exercises the following 12 weeks will focus on maximal strength. Training volume and intensity are described in the table below. Sets represent the number of circuit rounds while repetitions are maximal, which means that the resistance must be adjusted by the trainer so that the participant reaches exhaustion at the last prescribed repetition of each set. Training prescription for all exercises is made in accordance to the American College of Sport Medicine (ACSM) guidelines for strength development in older adults (ACSM, version 1998).

Weeks	Sets			Repetitions			Rest between sets (sec)
	Day 1	Day 2	Day 3	Day 1	Day 2	Day 3	
1-4	2	2	2	15-18	15-18	15-18	30
5-8	3	2	3	12-15	12-15	12-15	30
9-12	3	2	3	10	10	10	60
13-16	2	3	2	8	12	8	60
17-20	3	2	3	6	8	6	60

Following the strength portion of each session, an aerobic training will be prescribed. Participants will be asked to complete 10-20 minutes of cardiovascular exercises using different ergometers (treadmill, elliptical machines, cycling ergometers, rowing machines etc.), using steps or other forms of free aerobic exercises, as long as the cognitive load is minimized. Intensity will be monitored using a Borg scale (0-10) and the target will correspond to 5-6 for the first month, 6-7 for the second month and 7-8 for the last month. Details of the aerobic training are presented in the table below. Each session will end with a five (5) minute recovery period, which will consist of different breathing exercises and stretching.

Weeks	Sets	Duration (min)	Intensity (Borg 0-10)	Rest between sets (minutes)
1-4	2	10	5-6	1
5-8	2	10	5-6	1
9-12	2	10	6-7	1
13-16	2	10	6-7	1
17-20	2	10	7-8	1

Overall rate of perceived exertion (RPE) will be monitored using a Borg scale (0-10) 30 minutes after the end of each session. Participants will have to rate the overall difficulty of the training session. These results will provide information about the training load (session RPE – sRPE), monotony and strain.

6.1.3 VITAMIN D SUPPLEMENTATION

Participants will receive Vitamin D supplementation (1 tablet of 10,000IU of Vitamin D_3) or matching placebo three times per week in order to reach a weekly cumulative dose of 30,000 IU per week (equivalent of 4,258 IU daily). The vitamin D capsules will be provided by the Research Coordinator

(RC) in vials at baseline assessment and every four weeks to complete 5 vials during the training period. Vials will be returned by the participants to the RC at the end of each 4 weeks block. Rationale and bio-safety of the dose: The dose of 10,000 IU/day is currently approved by Health Canada as a supplementation for elderly patients. Heaney et al. have administered doses of 30,000 IU/day Vitamin D_3 to adult men for five months, with no significant changes in serum calcium concentrations or adverse reactions.[35-37] A comprehensive review of toxic effects of vitamin D found that the lowest level at which an adverse effect was observed was a serum calcidiol concentration of 200 nmol/l, corresponding to a daily intake of 40,000 IU. Therefore, three weekly doses of 10,000IU have a 9-fold weekly margin of safety of the established safe dose.

6.2 CONTROL/PLACEBOS

6.2.1 Control Cognitive Training

The active control cognitive training group will participate in skills training courses based on a published study from our research group[38]. Each session will consist of Internet search for 3 hotels, 3 touristic places and 3 restaurants, of participant's preference, in cities determined by the instructor at the beginning of each training session (a list of cities is presented in the manual of procedures for cognitive training). Another task will be watching a 20 minutes National Geographic documentary movie selected by the instructor at the beginning of each session; at the end of the movie participants need to answer three questions about the watched movie. These iPad tasks will be alternated between sessions to prevent boredom. The approximate time to perform both tasks is 20 minutes which is the approximate time to complete the dual-task training on the iPad.

Active control sessions will be held in groups of up to 8 participants and will take place in the same context and physical environment used for dual-task training sessions and lead by the same instructors. Studies compared dual-task training to single-task training, as a control condition, in order to control for exposure of the same specific tasks performed separately [38]. However, it can be argued that single-task training is not engaging or stimulating enough and does not provide a feeling of progression. In response to this, Walton et al. [39] recommended that cognitive training studies use preferably sham trainings whereby clinician interaction and participant expectation effects can be reliably matched. Therefore, this study will compare dual-task training to a non-specific intervention, more comparable to cognitively stimulating activities that older adults can naturally engage in. To assure same time exposure and same social interaction than in active intervention, the control intervention will be held in groups using the same type of device that is used for the active intervention.

6.2.2 Placebo Vitamin D supplementation

Participants will receive Vitamin D supplementation (1 tablet of 10,000IU of Vitamin D_3) or matching placebo three times per week in order to reach a weekly cumulative dose of 30,000 IU per week (equivalent of 4,258 IU daily). The vitamin D capsules will be provided by the Research Coordinator (RC) in vials at baseline assessment and every four weeks to complete 5 vials during the training period. Vials will be returned by the participants to the RC at the end of each 4 weeks block. Rationale and bio-safety of the dose: The dose of 10,000 IU/day is currently approved by Health Canada as a supplementation for elderly patients. Heaney et al. have administered doses of 30,000 IU/day Vitamin D_3 to adult men for five months, with no significant changes in serum calcium concentrations or adverse reactions.[35-37] A comprehensive review of toxic effects of vitamin D found that the lowest level at which an adverse effect was observed was a serum calcidiol concentration of 200 nmol/l, corresponding to a daily intake of 40,000 IU. Therefore, three weekly doses of 10,000IU have a 9-fold weekly margin of safety of the established safe dose.

6.2.3 Control Exercises (Tone-Stretching Exercises)

Participants assigned in the control exercise condition will take part in tone-stretching sessions in groups of up to 8 participants, supervised by a trainer. The exercises will be devoted to improve muscle tone, flexibility, without improving, strength, cardiorespiratory capacity (and mobility). Resistant load and number of repetitions will not progress across exercise sessions. The session will start with a 5-minute warm-up on the machine of their choice. This will be followed by 50 minutes of stretching exercises that will target the entire body, one joint at a time, from head to toes. Exercises will be performed in a seated position as much as possible. The stretching program will be based on the one developed by Stanziano et al.[39] The session will end with 5 minutes of relaxation. Variability in exercises performed between sessions will be encouraged in order to avoid progression throughout the entire program.

6.3 Supervision

All physical exercise training will be fully supervised by research assistants trained in exercise physiology or with kinesiology or physical therapy backgrounds or training. Training frequency is 3 days/week. Each session will take 90 minutes on average.

7.0 STUDY PROCEDURES

Baseline measurements will be obtained prior to randomization. There will be three measurement sessions: baseline, 6 months, and 12 months (Figure 1, Appendix I). Outcomes will be assessed by

trained assessors blinded to group allocation. As outlined in point 5, there are five main interventional/assessment centers. Recruitment will be occurring primary in clinics serving MCI populations already identifies in each site and from the community. In addition, older persons belonging to the community will be invited to participate through different recruitment methods, including case findings by phone interviews and recruitments in primary care and geriatrics clinics. Strategies to increase the awareness of the study will be implemented through explanatory brochures and a dedicated webpage: "www.synergictrial.com". Results of the mobility tests and laboratory examinations can be provided to the participant and to her/his family physician upon their request. Transportation will be paid when necessary. Finally, dissemination of our findings will be conducted in the community through open discussion sessions and booklets and publications in the local newsletter.

7.1 SCREENING SESSION (VISIT 1)

The following study procedures will be performed:

1. Written informed consent from study participant
2. Demographic Information
3. Physical Activity Scale for the Elderly Questionnaire (PASE)
4. he Mini Mental State Examination (MMSE)
5. The Montreal Cognitive Assessment (MoCA), Logical Memory 1 & 2, Consortium to Establish a Registry for Alzheimer's Disease (CERAD) Word List Recall
6. Geriatric Depression Scale (GDS-30)
7. Generalized Anxiety Disorder 7 (GAD-7)
8. Clinical Dementia Rating Scale (CDR)
9. Alzheimer's Disease Cooperative Study Activities of Daily Living (ADCS-ADL) and Lawton Brody Instrumental Activities Of Daily Living (IADL) Scale
10. Physical Activity Readiness Questionnaire Plus (PAR-Q+)
11. Physical Activity Tracker allocated to study participant

During the screening session, global cognitive function will be assessed using the MMSE and the MoCA[40,41] and Physical Activity Readiness Questionnaire Plus (PAR-Q+) will be also done. Physical activity trackers (ActiGraph) will be provided to each participant during the screening session. The physical activity tracker will be worn by participants until first baseline assessment visit, which is scheduled up to four weeks after screening. This strategy will assure an accurate record of physical activity for each participant prior to participating in the training protocol and will be correlated with the response on the PASE questionnaires.

Height, weight, seated and standing blood pressure, resting heart rate will be measured at screening session. Participants will undergo a clinical assessment with baseline to confirm current health status and eligibility for study using the PARQ+.

The participant will be asked to bring an informant with them to complete the CDR. If the participant does not have an informant to attend the visit with them, the informant portion of the assessment can be completed with the informant by phone the day of the visit. This will be arranged and completed by a trained Research Assistant or Coordinator.

7.2 BASELINE EVALUATION (VISIT 2)

Participants will undergo rigorous baseline evaluations, which will take place up to four weeks after the screening session. Baseline assessments will include neuroimaging, neuropsychological, and gait measures. These will be repeated at the 6-month and 12-month follow-up.

1. Log return of the Physical Activity Device
2. Blood Draw
3. Clinical Medical Questionnaire
4. Quality of Life Questionnaire (SF-36)
5. ADAS-Cog Plus Tests (ADAS-Cog 13 Tests, plus the Digit Span Forward and Backward WAIS-III, TMT A and B, Digit Symbol Test, Boston Naming Test)
6. D-KEFS Colour Word Interference Test
7. Short Physical Performance Battery (SPPB)
8. Six Minute Walk Test (6MWT)
9. Dual task control assessment while seated (mental task)
10. Gait assessment using Gait Mat and Accelerometers (when available)
11. Schedule Neuroimaging

7.2.1 TREATMENT ALLOCATION

Randomization Once informed consent has been obtained and the baseline assessment has been completed, participants will be allocated to each of the 5 arms. The randomization sequence of the participants will be generated in each site with a minimum of 4 participants recruited using a central, web-based randomization service. The Research Pharmacy will assign the 'vitamin D/placebo kits' in compliance with randomization lists generated by a computer program and allocation to groups will be done with sequentially numbered opaque sealed envelopes. A block randomization by 5 will be applied to ensure an appropriate balance of the characteristics of participants between each arm. Permuted

blocks of varying size will be employed to ensure balance over time. After baseline assessment, research personnel not involved in measurement or intervention will access the web-based randomization service to determine the group allocation.

7.2.2 BLINDING

In order to minimize a source of bias, this trial is planned to be a double-blinded study. Research personnel performing the outcome assessments will be blinded to group allocation. Participants will be blinded of intervention.

7.3 VISIT 3 – 6 MONTH FOLLOW-UP VISIT

1. Blood Draw
2. Clinical Medical Questionnaire
3. Physical Activity Scale for the Elderly Questionnaire (PASE)
4. Montreal Cognitive Assessment (MoCA)
5. Geriatric Depression Scale (GDS-30)
6. Generalized Anxiety Disorder 7 (GAD-7)
7. Clinical Dementia Rating Scale (CDR)
8. Alzheimer's Disease Cooperative Study Activities of Daily Living (ADCS-ADL)
9. Physical Activity Readiness Questionnaire Plus (PAR-Q+)
10. Quality of Life Questionnaire (SF-36)
11. ADAS-Cog Plus Tests (ADAS-Cog 13 Tests, plus the Digit Span Forward and Backward WAIS-III, TMT A and B, Digit Symbol Test, Boston Naming Test)
12. D-KEFS Colour Word Interference Test
13. Short Physical Performance Battery (SPPB)
14. Six Minute Walk Test (6MWT)
15. Dual task control assessment while seated (mental task)
16. Gait assessment using Gait Mat and Accelerometers (when available)
17. Schedule Neuroimaging

7.4 TELEPHONE FOLLOW-UP (9 MONTH POST-BASELINE TESTING)

Three months after the end of the intervention, a telephone follow-up will take place to document any falls that have been recorded on the participants' falls calendar. This will be done by the study Site Coordinator or Research Assistant.

7.5 VISIT 4 – 12 MONTH FOLLOW-UP VISIT

1. Blood Draw
2. Clinical Medical Questionnaire
3. Physical Activity Scale for the Elderly Questionnaire (PASE)
4. Montreal Cognitive Assessment (MoCA)
5. Geriatric Depression Scale (GDS-30)
6. Generalized Anxiety Disorder 7 (GAD-7)
7. Clinical Dementia Rating Scale (CDR)
8. Alzheimer's Disease Cooperative Study Activities of Daily Living (ADCS-ADL)
9. Physical Activity Readiness Questionnaire Plus (PAR-Q+)
10. Quality of Life Questionnaire (SF-36)
11. ADAS-Cog Plus Tests (ADAS-Cog 13 Tests, plus the Digit Span Forward and Backward WAIS-III, TMT A and B, Digit Symbol Test, Boston Naming Test)
12. D-KEFS Colour Word Interference Test
13. Short Physical Performance Battery (SPPB)
14. Six Minute Walk Test (6MWT)
15. Dual task control assessment while seated (mental task)
16. Gait assessment using Gait Mat and Accelerometers (when available)

7.6 STUDY PROCEDURE DESCRIPTIONS

7.6.1 Clinical-Medical Questionnaires

The presence of chronic diseases, comorbidities, chronic medications, history of previous falls will be recorded. Whenever possible, a family member will be also interviewed in order to confirm the medical history and functional status. Finally, fear of falling will be assessed using the Falls Efficacy Scale and the fear of falling scale.

7.6.2 Functionality score

Self-reported information will be collected at baseline, six and twelve month’s assessment on activities of daily living (ADLs) than are related to cognitive and physical capabilities. Specifically, the Alzheimer's Disease Cooperative Study Activities of Daily Living (ADCS-ADL) Inventory and IADL and Lawton Brody Instrumental Activities Of Daily Living (IADL) Scale will be used.

7.6.3 Blood Biomarkers

The following biomarkers will be drawn at the Baseline Visit,Visit 3 and Visit 4:

- Vitamin D levels, Calcium, PTH, and lipid level will be measured by conventional methods.
- Serum High Sensitive CRP, IL-6 , BDNF, and VEGF receptor 1, IGF-1 will be determined by standardized ELISA methods

Blood samples for this study (completed at baseline and 6-month visit) will be done by a certified study Research Coordinator or Research Assistant. Participants will be asked to fast and will be provided with a small breakfast after the draw is complete. Samples will be collected, processed and stored securely at the respective site in a -80C freezer used for research purposes. Samples will be batch shipped securely to a centralized laboratory for analysis. Sample tubes will be labeled appropriately by the Study Coordinator with only the participant's unique study identifier and time point of collection.

7.6.4 Imaging

The imaging protocol will follow the *Canadian Dementia Imaging Protocol* developed for CCNA by Dr. Simon Duchesne, Laval University and colleagues, the latter having already been used by members of the *Consortium pour l'identification précoce de la maladie d'Alzheimer- Québec (CIMA-Q)* Pre-post regional patterns of brain plasticity will be assessed using structural (high-resolution 3D T1-weighted images = 7 min) in order to get voxel-based volumetric and cortical thickness measures, as well as diffusion tensor imaging (DTI, 30 directions, with AP/PA correction scan = 7 min) to calculate fractional anisotropy and diffusivity in white matter tracts as well as derive tract-based statistics. Cerebrovascular integrity and pathology will be assessed using the following contrasts: PD/T2 = 5 min; FLAIR = 7 min, and T2* = 5min). Functional magnetic resonance imaging at rest (rsfMRI plus field map = 12 min) will also be acquired to measure change in data-driven functional networks. Metabolite levels will be measured from the right motor cortex, cingulate cortex, and hippocampus, using single voxel spectroscopy. The total time for the scan session including scanning and patient setup is normally less than 1.5 hours.

7.6.5 COGNITIVE OUTCOMES

Alzheimer Disease Assessment Scale- Plus (ADAS-Cog Plus**)**

Global cognition will be assessed using the cognitive section of the Alzheimer Disease Assessment Scale Plus (ADAS-Cog Plus)[25]. The scale consists of 10 brief cognitive tests assessing memory, language, executive function and praxis. Scores range from 0 to 90, with higher scores indicating higher severity of cognitive impairment. The ADAS-Cog has been a significant outcome measure in numerous

trials with MCI and AD. The ADAS-Cog Plus has marked advantages as an outcome measure in MCI populations since incorporates items concerning executive function and functionality. Reliability and validity has been confirmed. Since several items are harmonized with the classical ADAS-Cog, it will also provide comparability with previous or existing trials assessing intervention in MCI. The ADAS-Cog Plus includes additional testing in executive function and caregiver reported functionality. Executive function of the ADAS-Cog plus is composed by the following tests: Trail-Making Test (TMT) A & B, the WAIS-R Digit Symbol Substitution Test (DSST), Digit Span Test, and Category Fluency.

In brief, The TMT (A & B) assesses attention, speed, and mental flexibility. For this test, participants connect numbers (TMT-A) and numbers and letters (TMT-B) in order. Time to complete is the outcome of interest for this measure. Trails A is truncated at 3 min and Trails B at 5 min. The DSST measures speed and information processing. Participants are instructed to refer to a number-symbol key at the top of the page and to write the appropriate symbols under the corresponding numbers as quickly as possible. For the Digit Span Test, an auditory attention task, participants are asked to recall a series of numbers forward and backward. For Category Fluency, a measure of speed and flexibility of verbal thought, participants are asked to name as many items as possible in a specified category (vegetables); unique responses during the first minute were counted. Functionality will be assessed by the following five functionality questions : 1) "Ability of writing checks, paying bills, balancing checkbook;" 2) "Assembling tax records, business affairs, or papers"; 3) "Playing a game of skill, working on a hobby;" 4) "Keeping track of current events;" and 5) "Remembering appointments, family occasions, holidays, medications."

Trail Making Test (TMT) A & B (s)

The Trail Making Test (TMT) measures set shifting abilities. Both parts of the test consist of 25 circles distributed across a sheet of paper. For the first portion of the test (TMT A), the participant is required to draw lines to connect numbers in ascending order as quickly as possible. This part assesses visual perception and psychomotor rapidity. TMT B assesses mental shifting and the participant's attention ability, as they are required to do the same task as for TMT A, but alternating between numbers and letters. The participant is asked to perform the task as quickly as possible without lifting his/her pen from the paper. If the experimenter sees a mistake, she/he informs the participant. Results for both TMT A and B are reported as the number of seconds required to complete each task; therefore, higher scores reveal greater impairment. Trail A $\leq$ 40 seconds and Trail B $\leq$ 90 seconds are considered normal average values for these tests for older adults in the range of 60 to 75 years old. To index set shifting, the

difference between part B and part A completion times, smaller difference scores indicate better set shifting. [46]

Digit Span Test WAIS-III (Forwards & Backwards)

These tests measure short term and working memory. For the Digit Forward, participants are instructed to listen to each series of numbers in completion, followed by repeating the numbers in the same order. The final score is the sum of points from each correct trial. The maximum score is 16. For the Digit Backward, participants are instructed to listen to each series of numbers in completion, followed by repeating the numbers in the backward order. The final score is the sum of the points from each correct trial/series, with a maximum score of 14. The difference between the Digit Span Forward and Backward test scores is used as an index of the central executive component of working memory. Smaller difference scores indicate better working memory.[47,48]

Boston Naming Test

This test measures confrontational word retrieval skills. This version is composed by thirty pictures that are shown by the experimenter one by one. Participants are asked to name verbally each of the 15 nouns depicted in the black and white drawings. Following an error in naming, a semantic and/or phonemic cue is provided verbally to participants wherein the participants are given up to 20 seconds to respond following each cue. This task will be digitally recorded and stored. If the participant is not able to retrieve the name even after the semantic cue the experimenter gives a phonemic cue by speaking the part in bold (e.g. **sc**roll; Experimenter says: “sc”).

Generalized Anxiety Disorder 7 (GAD-7)

The Generalized Anxiety Disorder 7 is a self-rated measure of anxiety symptoms and its score is calculated by rating 0, 1, 2, and 3 to the response categories of “not at all”, “several days”, “more than half the days” and “nearly every day”, respectively, and adding together the scores for the seven questions. A score of 10 or higher has a sensitivity of 89% and a specificity of 82% for generalized anxiety disorder; a score of 5 is considered the cut-off for “mild” anxiety and 15 for “severe” anxiety.[45]

Geriatric Depression Scale (GDS-30)

The Geriatric Depression Scale was designed to discriminate the pattern of depressive symptoms from the general characteristics of the elderly population. It is a brief 30-item questionnaire in which participants are asked to respond by answering yes or no in reference to how they felt over the past week. The GDS was found to have a 92% sensitivity and a 89% specificity when evaluated against diagnostic criteria. Cut-off: normal-0-9; mild depressives-10-19; severe depressives-20-30.[49]

Mini Mental State Examination (MMSE)

A 30 point questionnaire used to assess mental status. The MMSE tests 5 areas of cognitive function: orientation, registration, attention and calculation, and recall and language.

Montreal Cognitive Assessment Test (MoCA)

The Montreal Cognitive Assessment (MoCA) was designed as a rapid screening instrument for mild cognitive dysfunction. It assesses different cognitive domains: attention and concentration, executive functions, memory, language, visuoconstructional skills, conceptual thinking, calculations, and orientation. Time to administer the MoCA is approximately 10 minutes. The total possible score is 30 points; a score of 26 or above is considered normal. [41]

7.6.6 MOBILITY OUTCOMES

Gait velocity and variability (single and dual-tasking)

Gait velocity will be assessed as the time taken to walk the middle 6 meters of 8 meters using the Portable Walkway System (ProtoKinetic® and/or GAITRite® Systems, Inc.) These devices automate the acquisition, analysis, and reporting of the objective parameters of gait as the subject walks down the walkway with embedded sensors. As a result, these gait mats allow detecting subtle changes in gait, including gait variability since it has been proven to have a fine discrimination when compared with other quantitative methods.[50] The gait mat is located in a well-lit room with start and end points marked on the floor one meter from either end of the mat to avoid recording acceleration and deceleration phases.

Instrumentation:

Gait performance will be assessed using electronic walkway systems (GAITRite® or PkMas®), which automatically determines spatiotemporal gait parameters from imbedded sensors activated by foot pressure. A computer processes the footsteps, providing data for both spatial and temporal parameters. In addition, among sites without electronic walkway systems, quantitative gait parameters will be assessed using wearable inertial sensors (Gulf Coast Inc.; Shimmer Inc.) worn bilaterally on the ankles and at the hip. Acceleration data will be used to calculate foot-contact and foot-off times determine step and stride timing data. Overall gait velocity is determined by timing the standard walk distance.

For walking conditions all individuals will perform walks along a 6 meter path while wearing hip and ankle worn accelerometers. Individuals will perform three main tasks: 1) preferred walking speed, 2) dual task walking and 3) fast walking. In the case that a participant uses an assistive aid (cane, walker), the tester will determine the participant’s capacity to walk safely without the use of the aid. With the

willingness of the participant, they will then perform one test walk without the use of the aid. If the participant is able to safely walk without the use of the assistive aid, all subsequent testing will be performed without its use. In all walks, participants will start 1 meter before the beginning of the 6 meter platform and continue to travel 1 meter past the end of the platform. This procedure is in place to ensure steady-state walking and to minimize any effects of acceleration and de-acceleration during the course of the walk.

The first walking block will measure self-selected or preferred walking. Participants will be instructed to "walk at a comfortable and secure pace". For preferred walking speed, a total of three walks will be performed. Participants with slow walking speed, less than 0.6m/s, or participants with lower limb disability, will be allowed to complete one walk if they are not able to perform the three trials. We require a minimum of approximately 20-steps to allow measurement of step-to-step variability. For example, an individual who walks at 1.5 m/s would usually only have 4-5 steps per walk. Therefore, we would need at least 3 repetitions. For those walking less than 1.0 m/s, two trials is commonly necessary. For those walking less than 0.5 m/s, a single walk is usually sufficient.

The second walking block will be dual-task walking and includes three separate walks. Before performing the walking task, participants will enumerate out loud the verbal outputs will be performed from a seated position prior to any walks and recorded. The seated assessments will be timed at 10 seconds for dual-tasks and will be performed in the beginning of all cognitive assessments to prevent practice effects in dual-task gait performance (see order of assessments for visit 2, 3 and 4). If during the assessment time restriction appears or the assessor can see the tasks are difficult for the individual, the minimum gait assessment required will be usual gait, naming animals and Serial 7s. The first dual-task administered will have the participant walk on the platform while simultaneously counting backward by serial 1's from 100 (100-99-98…) out loud. The second dual-task will have the participant repeat the walk but this time, while naming animals (fluency test). In the third dual-task, the participant will be asked to walk the platform while subtracting 7's from 100 (100-93-86…) out loud. During this trial, participants are encouraged to keep walking even if they cannot do the subtractions. These dual-task conditions selected are based on previous research which demonstrated that counting backwards requires both working memory and attention[51]. Participants will be instructed to pay attention to both gait and the cognitive task; if a participant stops either task during the trial, they will be prompted to continue. Not instructing to prioritize gait over cognitive task or vice-versa allows both gait and cognitive task to vary. This has previously been shown to provide a better representation of what happens naturally [52,53]. The evaluator will record any counting errors during walking.

The third and last walking block will include fast walking and the participants will be instructed to walk as fast and safe as they can without running.

Six-minute Walking Test (6MWT)

The 6MWT is used to quantitatively measure participants' exercise capacity by measuring the maximum distance that a person can walk in 6 minutes. The test is a modification of the 12-min walk/run test originally developed by Cooper [54] and is commonly used as an assessment of exercise capacity. The 6MWT is useful for predicting the maximal oxygen uptake related to cardiorespiratory fitness and is easily administered in clinical settings [55]. The 6MWT is believed to be a useful instrument because of its ease of administration and similarity to normal daily activities. It is a submaximal test of aerobic capacity which has been successfully used in MCI populations [56].

The 6MWT will be assessed by licensed and trained physical therapists. The participants are instructed to walk from one end of a 10-meter course to the other and back again as many times as possible in 6 min, while under the supervision of a physical therapist. After each minute, participants were informed of the time elapsed and were given standardized encouragement. The distance (meters) walked in 6 min was recorded [55].

Short Physical Performance Battery (SPPB)

It is a widely used instrument for quantifying lower extremity function and mobility in older adults. It is a group of measures that combines the results of the gait speed, chair stand and balance tests [57]. It has been used as a predictive tool for possible disability and can aid in the monitoring of function in older people. The scores range from 0 (worst performance) to 12 (best performance). The SPPB has been shown to have predictive validity showing a gradient of risk for mortality, nursing home admission, and disability. [58]

7.6.7 FUNCTIONAL OUTCOMES AND COMBINED SCORE OF COGNITION AND FUNCTIONALITY (POOLED INDEX)

<u>Functional Outcomes</u>: Activities of daily living and everyday function will be evaluated using the use the ADCS which is a 23-item informant-rated questionnaire that measures, in a range of 0 to 78, an individual's performance of activities of daily living.

<u>Pooled Index</u>: A pool index including measures of cognition, mobility and functionality will be used as a secondary outcome. The cognitive decline seen with aging, due to neurodegenerative or vascular mechanisms, affects not only cognition but also mood, behavior and the ability to perform activities of

daily living. Therefore, treatments are considered successful if they slow down the progression of cognitive decline and maintain functionality and independency. Studies conducted in psychiatric patients and therapies for rheumatoid arthritis have demonstrated the applicability of a pooled index (PI) in measuring overall function and how changes in PI scores can be analyzed to quantify improvement or worsening conditions over time[60-62]. To apply this approach in our cohort, we constructed the **Cognition and Functionality Pooled Index** with six continuous variables sensitive to cognition function in older adults: Trail- Making Test, parts A and B (executive function), Rey Auditory Verbal Learning Test (RAVLT) (semantic memory, verbal learning), Geriatric Depression Scale [GDS] (mood), Alzheimer's disease Activities of Daily Living-International Scale [ADL-IS] (functionality), and dual-task gait cost. The efficiency of a Pool Index is greatest when the combined variables are poorly correlated ($r<0.4$) which reduces variance of PI. The suitability of our 6 source variables was confirmed by calculating the strength of pairwise correlations using Pearson Correlation Coefficient. The pooled index is created by converting all individual scores from the six tests to standardized *z* scores by subtracting the baseline group mean and dividing by the baseline group SD, and then averaging the standardized *z* scores. For tests with more than 1 component, the component scores were first averaged to create a single *z* score for each test (e.g., *z* scores for Trails A and B were averaged to create a single Trails *z* score) so that all tests were weighted equally, and then the mean *z* scores for the 6 tests was calculated.

7.6.8 QUALITY OF LIFE OUTCOMES

Health related quality of life will be evaluated using the SF 36 that provides weightings for quality adjusted life year (QALYs). The SF 36 captures health states based on the following domains: 1) mobility; 2) self-care; 3) usual activities; 4) pain and 5) anxiety or depression. We will calculate scores using the established methodology to determine if there is a statistically significant difference in the incremental cost per QALY change across the 5 arms.

7.6.9 HEALTH RESOURCE UTILIZATION

At Visits 2 and 3, we will ask participants to report any visit to: 1) health care professionals; 2) admissions or visits to hospital; and 3) laboratory work using the health resource utilization questionnaire. This questionnaire has been previously described and supported in previous studies [Neumann PJ, Goldie SJ, Weinstein MC: Preference-based measures in economic evaluation in health care. Annual review of public health. 2000, 21: 587-611. 10.1146/annurev.publhealth.21.1.587.]. We will estimate total health care related costs over the 12 months from a Canadian health care system

perspective. Participants will be instructed to specify total health care expenditure and report the reason for each item. Additionally, participants will be instructed to report health care expenditure due to any adverse events associated with our study. On a per participant basis, costs will be assigned to health care resource utilization using a fully allocated hospital cost model (for in-patient costs) and provincial guides to medical fees (for outpatient costs). Case analyses will consider the costs of all health care resource use and our sensitivity analyses will include only intervention related health care resource costs and a complete case analysis.

8. ANALYSIS

Analysis, planned analysis, frequency of analysis, and correction for multiple testing

Analysis will be conducted as intention to treat and as per-protocol analysis. For the primary endpoint (ADAS-Cog plus), the change from baseline to six months and 12 months will be assessed using a repeated measures analysis of covariance (ANCOVA) model incorporating the baseline values as covariates. Observing a statistically significant difference in the primary outcome will be considered preliminary evidence of efficacy. Additionally, reduction of prevalence rate of abnormal ADAS-Cog plus score (dichotomous variable) will be asses as a primary outcome.

We will also report variances, co-variances, and effect sizes, as well as sampling feasibility (i.e., ease of recruitment, recruitment rate, withdrawal rate). Descriptive statistics for demographic and baseline characteristics will be provided with means and standard deviations, or medians and the interquartile range where appropriate, for continuous characteristics and frequencies and percentages for categorical variables.

If the within-group comparison is statistically significant, Tukey's multiple comparisons will identify significant pairwise differences. Similarly, for the secondary endpoints, six month changes in gait performance and secondary cognitive outcomes of interest will be performed using one way analyses of variance. For group comparisons that are statistically significant, pairwise comparisons will be made using Tukey's multiple comparisons tests. All statistical tests will be two-tailed, and a *p*-value of less than 0.05 will be considered to indicate statistical significance. All calculations will be made using SPSS software package version 23.0 (SPSS Inc., Chicago, IL).

Secondary analyses will be also performed according to an intention-to-treat principle and per protocol analysis. Frequency of the analyses: preliminary analysis will be performed at month 6 to ascertain descriptive characteristics at baseline assessment. Final efficacy analysis will be performed at the end of the trial since no safety issues are involved in this study. The statistical significance of the results will be

determined by Hochberg's variation of the Bonferroni procedure for multiple testing.[39] An economical analysis will be also performed addressing health resources utilization.

9. ADVERSE EVENT (AE) AND SERIOUS ADVERSE EVENT REPORTING (SAE)

Adverse events will be recorded for subjects starting at the time of signing the Informed Consent and their discontinuation within the study. All AEs will be recorded on an ongoing paper log, which will be reviewed by each site leader and forwarded to the team leader at London site, who will determine both the intensity of the event and the relationship of the event to study procedures. Based on the outcome it will be up to the team leader to medically determine if it is justified for the participant to continue in the study or terminate their participation.

SAEs will be recorded on a paper log, starting at the time of signing the Informed Consent and up to the end of their participation within the study. SAEs which are classified as unanticipated will be reported to local REBs. An SAE is defined as any incident that is unexpected, and related or possibly related to participation in the research study. SAEs will be reviewed and monitored by each site team leader until resolved.

9.1 CONCOMITANT MEDICATIONS

Antacids (Magnesium-containing): Hypermagnesemia may develop when these agents are used concurrently with Vitamin D, particularly in patients with chronic renal failure.

Anticonvulsants (Phenytoin, Phenobarbital): Decreased Vitamin D effects may occur when certain anticonvulsants are administered, as they may induce hepatic microsomial enzymes and accelerate the conversion of Vitamin D to inactive metabolites.

Cholestyramine, Colestipol, Mineral Oil: Intestinal absorption of Vitamin D may be impaired. Patients on cholestyramine or colestipol should be advised to allow as much time as possible between the ingestion of these drugs and Vitamin D.

Digoxin: Vitamin D should be used with caution in patients on digoxin as hypercalcemia (which may result with Vitamin D use) may precipitate cardiac arrhythmias.

Thiazide diuretics: There is an increased risk of hypercalcaemia if vitamin D is coadministered with thiazide diuretics and calcium. Plasma-calcium concentrations should be monitored in patients receiving the drugs concurrently (Source: Martindale).

Different Vitamin D analogues should be administered concurrently.

9.2 RESCUE MEDICATION AND RISK MANAGEMENT

Higher doses of Vitamin D may lead to hypervitaminosis D manifested by hypercalcemia and its sequelae. Treatment of acute or chronic intoxication includes withdrawal of Vitamin D3 and any calcium supplements, maintenance of a low calcium diet and if needed corticosteroids or calciuric diuretics, such as furosemide and ethacrymic acid to decrease serum calcium concentrations. All participants will be monitored by site physicians and trained staff should any adverse events arise participants will have access to a phone number and e-mail address provided on the copy of their signed consent form. They will be informed to use these contacts to inform the Principal Investigator about concerns that may be associated with treatment received in each site.

9.3 PREMATURE WITHDRAWAL/DISCONTINUATION CRITERIA

Participants may be withdrawn or be discontinued from the study for any of the following reasons:

1. At the participant's request (withdrawal of consent)
2. At the discretion of the investigator, if deemed appropriate, for any reason
3. If an intolerable or life threatening adverse event occurs

Or if any of the following develop:

4. renal dysfunction
5. dementia
6. severe depression/anxiety/schizophrenia
7. hypercalcaemia and/or hypermagnesemia

Participant data collected to the date of discontinuation or withdrawal will be included in the analysis.

10. ETHICAL CONSIDERATIONS

This study will be conducted in compliance with the outlined protocol, International Conference on Harmonization Good Clinical Practice (ICH-GCP), and all applicable regulatory requirements.

All relevant documents for this study will be submitted to the appropriate Research Ethics Board (REB) for review. Written approval from the REB must be obtained prior to entering and testing participants within this study. The REB will be notified of any protocol amendments regarding new study procedures which may occur during the course of the study. Written approval from the REB on these amendments must be received prior to implementation of any new procedures.

10.1 CONFIDENTIALITY OF PARTICIPANT RECORDS

Participants will be identified by a study identification number. It will be necessary to collect the participants name and contact information as this study will require several scheduling procedures. This information will be kept separate from the participant study identification number.

11. ADMINISTRATIVE REQUIREMENTS

11.1 PROTOCOL AMENDMENTS

The leaders of the study, in collaboration with members of the team, may modify the protocol at any time during the study. Protocol amendments will require REB approval prior to implementation of any changes.

11.2 COMPLETION OF CASE REPORT FORMS (CRFS)

All sites participating in his study will be trained on completion of CRFs. The primary investigators at all sites are responsible for the CRFs with respect to completeness, accuracy, and clarity.

11.3 MONITORING PROCEDURES

All sites will use the same data collection forms to maintain consistency. Monitoring procedures will be performed using the LORIS system platform for uploading data specially developed for the SYNERGIC study by CCNA informatics team platform.

12. PUBLICATION POLICY

The SYNERGIC Trial will follow CCNA publication policy.

12.1 PARTICIPATING CENTERS

The following centers will be participating in this study:

- Gait and Brain Lab & Neurovascular Research Lab, The University of Western Ontario, London, ON
- Imaging Research Laboratories. Robarts Research Institute, The University of Western Ontario, London, ON
- Aging, Mobility, and Cognitive Neuroscience Lab, University of British Columbia, Vancouver, BC
- Brain Research Centre, Centre for Hip Health and Mobility

- Unité de Neuroimagerie Fonctionnelle. Centre de recherche, Institut universitaire de gériatrie de Montréal, Université de Montréal, Montreal
- Division of Geriatrics, McGill Centre of Excellence in Aging and Chronic Disease, McGill University
- PERFORM Center, Concordia University/Centre de recherche, Institut universitaire de gériatrie de Montréal. Université de Montréal (CRIUGM)
- Department of Psychology, Concordia University
- Department of Medicine, Division of Neurology, University of Alberta
- The Sun Life Financial Movement Disorders Research and Rehabilitation Centre, Wilfrid Laurier University
- Centre for Community, Clinical and Applied Research Excellence (CCCARE), University of Waterloo
- Balance, Mobility and Falls Clinic at Toronto Rehab Rehabilitation Affiliate Network of the Centre for Stroke Recovery, Sunnybrook

Figure 1: CONSORT flowchart for the SYNERGIC Trial

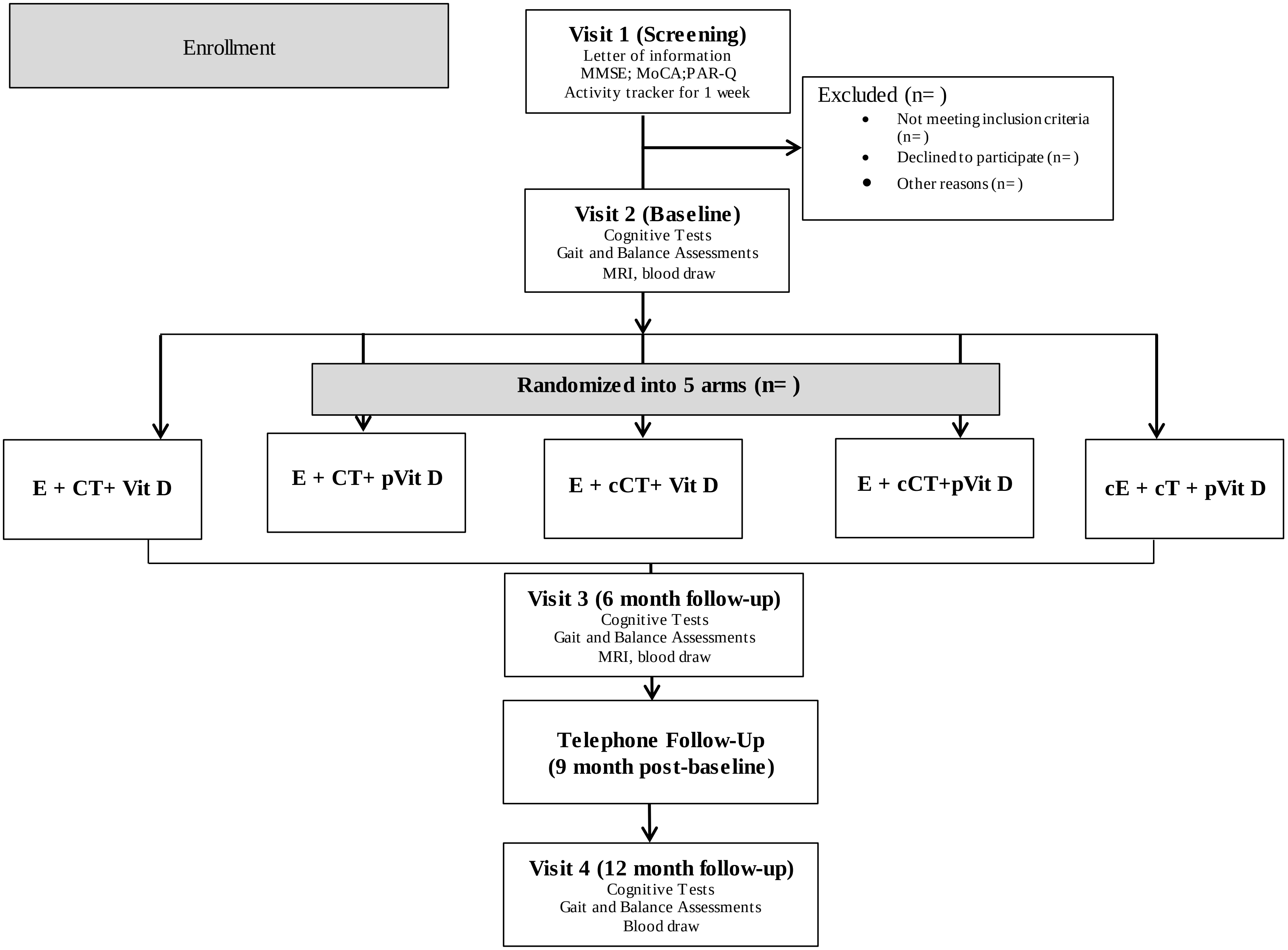

Figure 2. Schematic Timeline of Synergic Trial

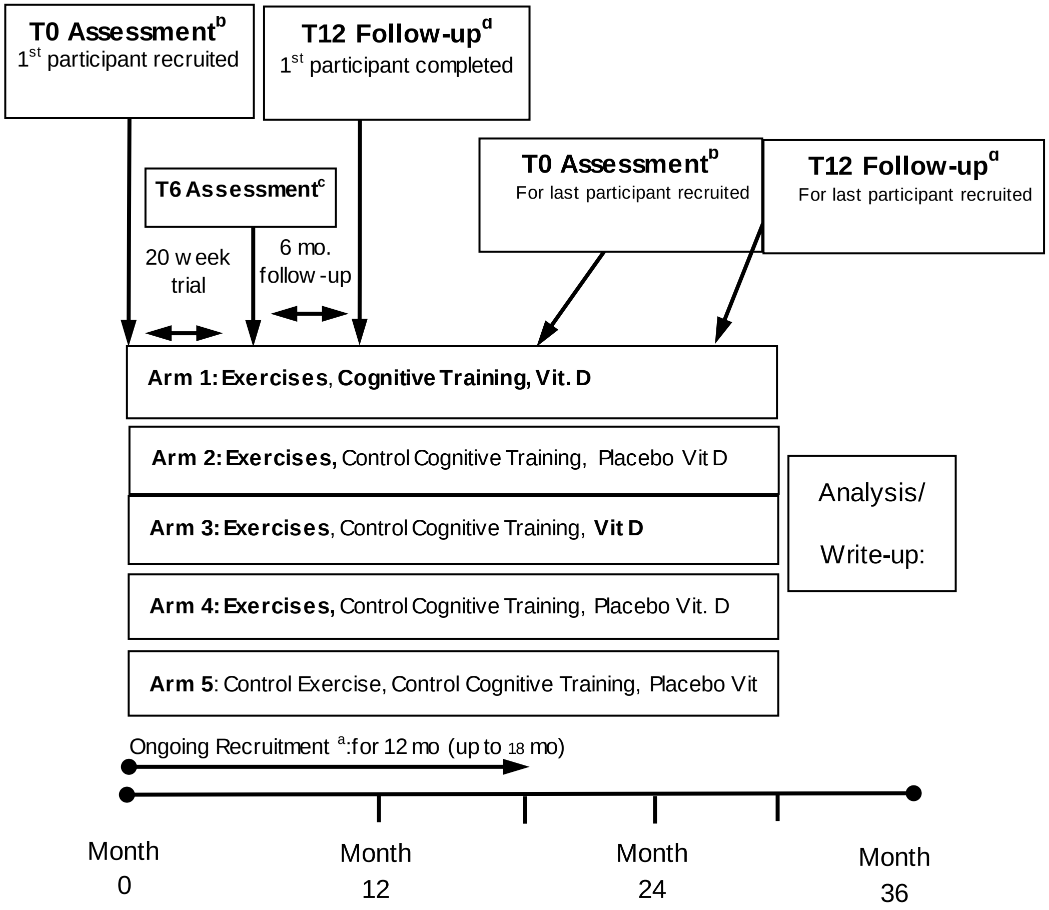

(a) Recruitment of participants will be an on-going process with individuals being assigned to groups as they are enrolled. Recruitment period is expected to be 24 months, at a recruitment rate of 2.5 patients per month per main site (conservative estimate). However, recruitment may continue up to 36 months after the first individual has been recruited. Recruitment period of 24 months

(b) Baseline assessments (T0) will be completed within one-week of participant starting the specific intervention/placebo (i.e. month 0 for the first participant recruited; and month 30 for the last participant recruited). Participants will return to the clinic six months(c) after starting the specific intervention/placebo (T6) to complete the post intervention assessment. Six months participants will return to the clinic again (T12) at which time their final assessment will be completed. The same assessments at T0 will be done.

** 9 months after their first baseline assessment (T0) i.e. 3 months post-intervention, there will be a follow up phone call.

Figure 3. SYNERGIC Patient Flow Chart

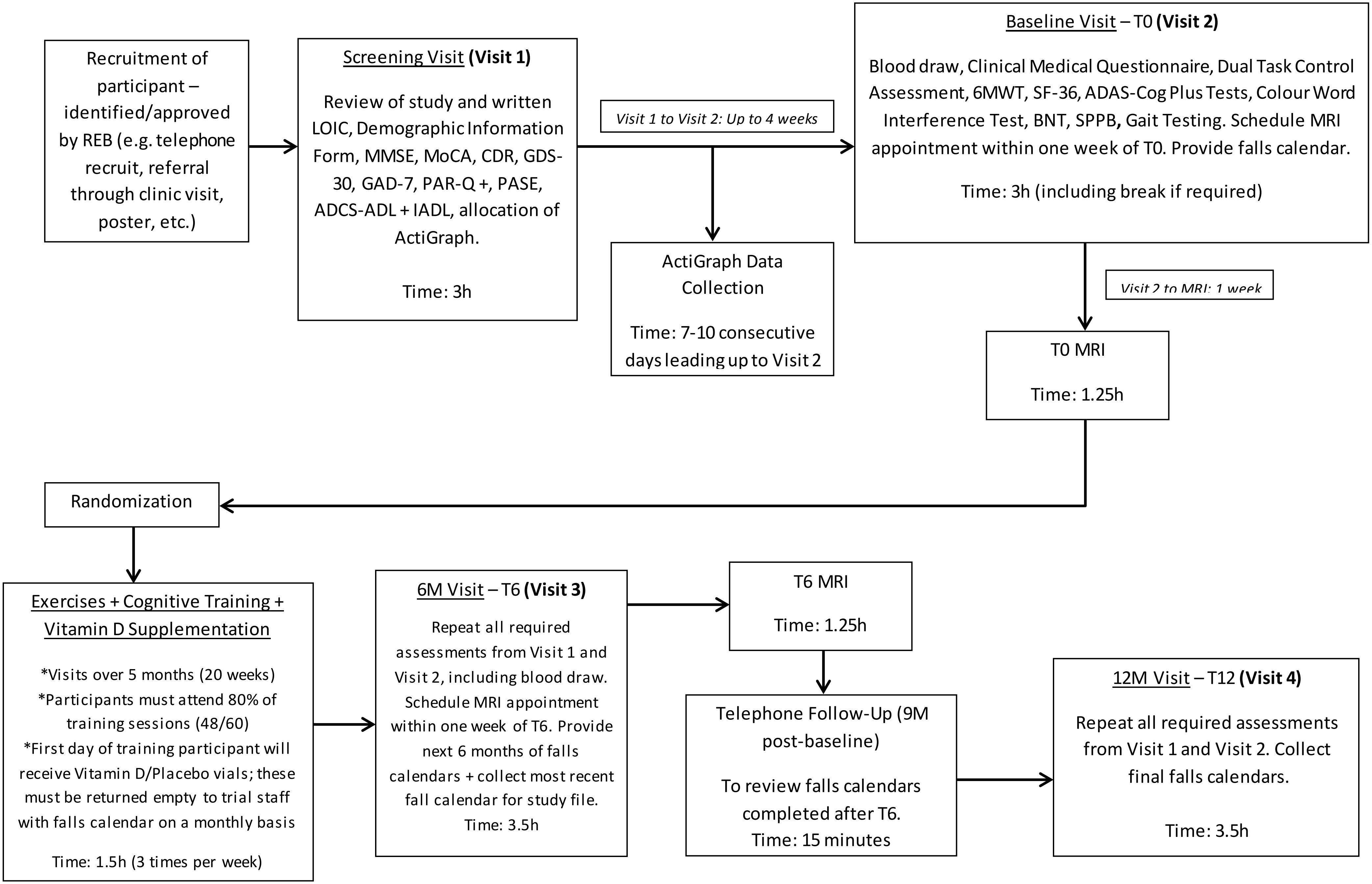

13. Reference List

1. Bherer L, Erickson KI, Liu-Ambrose T: **Physical exercise and brain functions in older adults.** *J Aging Res* 2013, **2013:** 197326.

2. Liu-Ambrose T, Nagamatsu LS, Voss MW, Khan KM, Handy TC: **Resistance training and functional plasticity of the aging brain: a 12-month randomized controlled trial.** *Neurobiol Aging* 2012, **33:** 1690-1698.

3. Colcombe S, Kramer AF: **Fitness effects on the cognitive function of older adults: a meta-analytic study.** *Psychol Sci* 2003, **14:** 125-130.

4. Langlois F, Vu TT, Chasse K, Dupuis G, Kergoat MJ, Bherer L: **Benefits of physical exercise training on cognition and quality of life in frail older adults.** *J Gerontol B Psychol Sci Soc Sci* 2013, **68:** 400-404.

5. Sage MD, Almeida QJ: **A positive influence of vision on motor symptoms during sensory attention focused exercise for Parkinson's disease.** *Mov Disord* 2010, **25:** 64-69.

6. Sage MD, Almeida QJ: **Symptom and gait changes after sensory attention focused exercise vs aerobic training in Parkinson's disease.** *Mov Disord* 2009, **24:** 1132-1138.

7. Cotman CW, Berchtold NC, Christie LA: **Exercise builds brain health: key roles of growth factor cascades and inflammation.** *Trends Neurosci* 2007, **30:** 464-472.

8. Nagamatsu LS, Handy TC, Hsu CL, Voss M, Liu-Ambrose T: **Resistance training promotes cognitive and functional brain plasticity in seniors with probable mild cognitive impairment.** *Arch Intern Med* 2012, **172:** 666-668.

9. Kueider AM, Parisi JM, Gross AL, Rebok GW: **Computerized cognitive training with older adults: a systematic review.** *PLoS One* 2012, **7:** e40588.

10. Reijnders J, van HC, van BM: **Cognitive interventions in healthy older adults and people with mild cognitive impairment: a systematic review.** *Ageing Res Rev* 2013, **12:** 263-275.

11. Li KZ, Roudaia E, Lussier M, Bherer L, Leroux A, McKinley PA: **Benefits of cognitive dual-task training on balance performance in healthy older adults.** *J Gerontol A Biol Sci Med Sci* 2010, **65:** 1344-1352.

12. Ballesteros S, Prieto A, Mayas J, Toril P, Pita C, Ponce de LL *et al.*: **Brain training with non-action video games enhances aspects of cognition in older adults: a randomized controlled trial.** *Front Aging Neurosci* 2014, **6:** 277.

13. Chapman SB, Aslan S, Spence JS, Hart JJ, Jr., Bartz EK, Didehbani N *et al.*: **Neural mechanisms of brain plasticity with complex cognitive training in healthy seniors.** *Cereb Cortex* 2015, **25:** 396-405.

14. Annweiler C, Montero-Odasso M, Muir SW, Beauchet O: **Vitamin D and Brain Imaging in the Elderly: Should we Expect Some Lesions Specifically Related to Hypovitaminosis D?** *Open Neuroimag J* 2012, **6:** 16-18.

15. Beauchet O, Annweiler C, Verghese J, Fantino B, Herrmann FR, Allali G: **Biology of gait control: Vitamin D involvement.** *Neurology* 2011, **76:** 1617-1622.

16. Muir SW, Montero-Odasso M: **Effect of vitamin D supplementation on muscle strength, gait and balance in older adults: a systematic review and meta-analysis.** *J Am Geriatr Soc* 2011, **59:** 2291-2300.

17. Annweiler C, Schott AM, Rolland Y, Blain H, Herrmann FR, Beauchet O: **Dietary intake of vitamin D and cognition in older women: a large population-based study.** *Neurology* 2010, **75:** 1810-1816.

18. Annweiler C, Schott AM, Berrut G, Chauvire V, Le Gall D, Inzitari M *et al.*: **Vitamin D and ageing: neurological issues.** *Neuropsychobiology* 2010, **62:** 139-150.

19. Annweiler C, Montero-Odasso M, Llewellyn DJ, Richard-Devantoy S, Duque G, Beauchet O: **Meta-analysis of memory and executive dysfunctions in relation to vitamin D.** *J Alzheimers Dis* 2013, **37:** 147-171.

20. Annweiler C, Muir SW, Nabeel S, Gopaul K, Beauchet O, Montero-Odasso M: **Higher serum vitamin D concentration is associated with better balance in older adults with supra-optimal vitamin D status.** *J Am Geriatr Soc* 2013, **61:** 163-165.

21. Ostbye T, Crosse E: **Net economic costs of dementia in Canada.** *CMAJ* 1994, **151:** 1457-1464.

22. Toronto Rehabilitation Institute. Fact Sheet: Disabilities and aging in Canada. http://www.torontorehab.com/research/documents/iDAPT-Fact%20Sheet-%20Disabilities%20in%20Canada.pdf . 8-26-2009.

Ref Type: Electronic Citation

23. Ohman H, Savikko N, Strandberg TE, Pitkala KH: **Effect of physical exercise on cognitive performance in older adults with mild cognitive impairment or dementia: a systematic review.** *Dement Geriatr Cogn Disord* 2014, **38:** 347-365.

24. Suzuki T, Shimada H, Makizako H, Doi T, Yoshida D, Ito K *et al.*: **A randomized controlled trial of multicomponent exercise in older adults with mild cognitive impairment.** *PLoS One* 2013, **8:** e61483.

25. Skinner J, Carvalho JO, Potter GG, Thames A, Zelinski E, Crane PK *et al.*: **The Alzheimer's Disease Assessment Scale-Cognitive-Plus (ADAS-Cog-Plus): an expansion of the ADAS-Cog to improve responsiveness in MCI.** *Brain Imaging Behav* 2012, **6:** 489-501.

26. Takeda A, Loveman E, Clegg A, Kirby J, Picot J, Payne E *et al.*: **A systematic review of the clinical effectiveness of donepezil, rivastigmine and galantamine on cognition, quality of life and adverse events in Alzheimer's disease.** *Int J Geriatr Psychiatry* 2006, **21:** 17-28.

27. Crane PK, Carle A, Gibbons LE, Insel P, Mackin RS, Gross A *et al.*: **Development and assessment of a composite score for memory in the Alzheimer's Disease Neuroimaging Initiative (ADNI).** *Brain Imaging Behav* 2012, **6:** 502-516.

28. Albert MS, DeKosky ST, Dickson D, Dubois B, Feldman HH, Fox NC *et al.*: **The diagnosis of mild cognitive impairment due to Alzheimer's disease: recommendations from the National Institute on Aging-Alzheimer's Association workgroups on diagnostic guidelines for Alzheimer's disease.** *Alzheimers Dement* 2011, **7:** 270-279.

29. Lawton MP, Brody EM: **Assessment of older people: self-maintaining and instrumental activities of daily living.** *Gerontologist* 1969, **9:** 179-186.

30. American Psychiatric Association: *Diagnostic and Statistical Manual of Mental Disorders (4th ed. - Text Revision; [DSV-IV-TR]).* Washington, DC: Author; 2000.

31. Fiatarone Singh MA, Gates N, Saigal N, Wilson GC, Meiklejohn J, Brodaty H *et al.*: **The Study of Mental and Resistance Training (SMART) study-resistance training and/or cognitive training in mild cognitive impairment: a randomized, double-blind, double-sham controlled trial.** *J Am Med Dir Assoc* 2014, **15:** 873-880.

32. Bherer L, Kramer AF, Peterson MS, Colcombe S, Erickson K, Becic E: **Training effects on dual-task performance: are there age-related differences in plasticity of attentional control?** *Psychol Aging* 2005, **20:** 695-709.

33. Bherer L, Kramer AF, Peterson MS, Colcombe S, Erickson K, Becic E: **Testing the limits of cognitive plasticity in older adults: application to attentional control.** *Acta Psychol (Amst)* 2006, **123:** 261-278.

34. Bherer L, Kramer AF, Peterson MS, Colcombe S, Erickson K, Becic E: **Transfer effects in task-set cost and dual-task cost after dual-task training in older and younger adults: further evidence for cognitive plasticity in attentional control in late adulthood.** *Exp Aging Res* 2008, **34:** 188-219.

35. Vieth R: **The mechanisms of vitamin D toxicity.** *Bone Miner* 1990, **11:** 267-272.

36. Vieth R, Chan PC, MacFarlane GD: **Efficacy and safety of vitamin D3 intake exceeding the lowest observed adverse effect level.** *Am J Clin Nutr* 2001, **73:** 288-294.

37. Venning G: **Recent developments in vitamin D deficiency and muscle weakness among elderly people.** *BMJ* 2005, **330:** 524-526.

38. Lussier M, Brouillard P, Bherer L: **Limited Benefits of Heterogeneous Dual-Task Training on Transfer Effects in Older Adults.** *J Gerontol B Psychol Sci Soc Sci* 2015.

39. Stanziano DC, Roos BA, Perry AC, Lai S, Signorile JF: **The effects of an active-assisted stretching program on functional performance in elderly persons: a pilot study.** *Clin Interv Aging* 2009, **4:** 115-120.

40. Julayanont P, Brousseau M, Chertkow H, Phillips N, Nasreddine ZS: **Montreal Cognitive Assessment Memory Index Score (MoCA-MIS) as a predictor of conversion from mild cognitive impairment to Alzheimer's disease.** *J Am Geriatr Soc* 2014, **62:** 679-684.

41. Nasreddine ZS, Phillips NA, Bedirian V, Charbonneau S, Whitehead V, Collin I *et al.*: **The Montreal Cognitive Assessment, MoCA: a brief screening tool for mild cognitive impairment.** *J Am Geriatr Soc* 2005, **53:** 695-699.

42. Vakil E, Greenstein Y, Blachstein H: **Normative data for composite scores for children and adults derived from the Rey Auditory Verbal Learning Test.** *Clin Neuropsychol* 2010, **24:** 662-677.

43. Rey A: **L'examen clinique en psychologie.** 1958.

44. Schmidt M: *Rey auditory verbal learning test: A handbook*. Western Psychological Services Los Angeles; 1996.

45. Strauss E, Sherman EM, Spreen O. A compendium of neuropsychological tests. 2006. New York: Oxford University Press.

Ref Type: Generic

46. Tombaugh TN: **Trail Making Test A and B: normative data stratified by age and education.** *Arch Clin Neuropsychol* 2004, **19:** 203-214.

47. Yamamoto D, Kazui H, Takeda M: **[Wechsler Adult Intelligence Scale-III (WAIS-III)].** *Nihon Rinsho* 2011, **69 Suppl 8:** 403-407.

48. Liu-Ambrose T, Nagamatsu LS, Graf P, Beattie BL, Ashe MC, Handy TC: **Resistance training and executive functions: a 12-month randomized controlled trial.** *Arch Intern Med* 2010, **170:** 170-178.

49. Yesavage JA, Brink TL, Rose TL, Lum O, Huang V, Adey M *et al.*: **Development and validation of a geriatric depression screening scale: a preliminary report.** *J Psychiatr Res* 1982, **17:** 37-49.

50. Shore WS, deLateur BJ, Kuhlemeier KV, Imteyaz H, Rose G, Williams MA: **A comparison of gait assessment methods: Tinetti and GAITRite electronic walkway.** *J Am Geriatr Soc* 2005, **53:** 2044-2045.

51. Hittmair-Delazer M, Semenza C, Denes G: **Concepts and facts in calculation.** *Brain* 1994, **117 (Pt 4):** 715-728.

52. Verghese J, Kuslansky G, Holtzer R, Katz M, Xue X, Buschke H *et al.*: **Walking while talking: effect of task prioritization in the elderly.** *Arch Phys Med Rehabil* 2007, **88:** 50-53.

53. Yogev-Seligmann G, Hausdorff JM, Giladi N: **The role of executive function and attention in gait.** *Mov Disord* 2008, **23:** 329-342.

54. Cooper KH: **A means of assessing maximal oxygen intake: correlation between field and treadmill testing.** *JAMA* 1968, **203:** 201-204.

55. Steffen TM, Hacker TA, Mollinger L: **Age- and gender-related test performance in community-dwelling elderly people: Six-Minute Walk Test, Berg Balance Scale, Timed Up & Go Test, and gait speeds.** *Phys Ther* 2002, **82:** 128-137.

56. Makizako H, Shimada H, Doi T, Park H, Yoshida D, Suzuki T: **Six-Minute Walking Distance Correlated with Memory and Brain Volume in Older Adults with Mild Cognitive Impairment: A Voxel-Based Morphometry Study.** *Dementia and Geriatric Cognitive Disorders Extra* 2013, **3:** 223-232.

57. Guralnik JM, Ferrucci L, Pieper CF, Leveille SG, Markides KS, Ostir GV *et al.*: **Lower extremity function and subsequent disability consistency across studies, predictive models, and value of gait speed alone compared with the Short Physical Performance Battery.** *The Journals of Gerontology Series A: Biological Sciences and Medical Sciences* 2000, **55:** M221-M231.

58. Guralnik JM, Simonsick EM, Ferrucci L, Glynn RJ, Berkman LF, Blazer DG *et al.*: **A short physical performance battery assessing lower extremity function: association with self-reported disability and prediction of mortality and nursing home admission.** *J Gerontol* 1994, **49:** M85-M94.

59. Lord SR, Menz HB, Tiedemann A: **A physiological profile approach to falls risk assessment and prevention.** *Phys Ther* 2003, **83:** 237-252.

60. Speechley M, Barton JC, Passmore L, Harrison H, Reboussin DM, Harris EL *et al.*: **Potential nonresponse bias in a clinical examination after initial screening using iron phenotyping and HFE genotyping in the hemochromatosis and iron overload screening study.** *Genet Test Mol Biomarkers* 2009, **13:** 721-728.

61. Smythe HA, Helewa A, Goldsmith CH: **"Independent assessor" and "pooled index" as techniques for measuring treatment effects in rheumatoid arthritis.** *J Rheumatol* 1977, **4:** 144-152.

62. Goldsmith CH, Smythe HA, Helewa A: **Interpretation and power of a pooled index.** *J Rheumatol* 1993, **20:** 575-578.

APPENDIX I

		Visit Table				
**Procedure**	Visit 1 Screening	Visit 2 Baseline	Randomization	Visit 3 6M Visit	Telephone Follow-up (9M post-Baseline)	Visit 4 12M Visit
Written Informed Consent	X					
Demographic Information	X					
PASE Questionnaire	X			X		X
Mini Mental State Examination (MMSE)	X					
Montreal Cognitive Assessment (MoCA) Logical Memory 1 & 2 * CERAD Word List Recall *	X			X		X
Geriatric Depression Scale (GDS-30)	X			X		X
Generalized Anxiety Disorder 7 (GAD-7)	X			X		X
Clinical Dementia Rating (CDR)	X			X		X
Physical Activity Readiness Questionnaire Plus (PAR-Q +)	X					
ADCS-ADL and IADL	X			X		X
Clinical Medical Questionnaire		X		X		X
Dual Task Control Assessment		X		X		X
Six Minute Walk Test (6MWT) ^		X		X		X
Quality of Life Questionnaire (SF-36)		X		X		X
ADAS-Cog 13 (+ tests **)		X		X		X
Digit Span Forward and Backward WAIS-III **		X		X		X
TMT A and B **		X		X		X
Digit Symbol Test **		X		X		X
Boston Naming Test **		X		X		X
Colour Word Interference Test		X		X		X
Envelope Test		X		X		X
Short Physical Performance Battery (SPPB)		X		X		X
Gait Assessment using Gait Mat and accelerometers (when available)		X		X		X
Neuroimaging (MRI)		X		X		
Blood Draw		X		X		X
Falls Calendar ^^		X		X	X	X

***** These tests are implemented to harmonize with inclusion criteria for COMPASS-ND and are not mandatory if the MoCA score ≤ 24.
****** Testing included in the ADAS Cog Plus.
^ 6MWT can be completed at the Baseline Visit or at Day 1 of intervention sessions at the gym facilities. Please note date and location of the 6MWT in patient file.
^^ Calendar will be given to participant to complete and will be submitted to Research Staff at exercise training.

Statistical Analysis Plan
(SAP)

SYNchronizing **E**xercises, **R**emedies in **G**aIt and **C**ognition (SYNERGIC) Trial

Version: Final
Authors: Manuel Montero-Odasso, MD, PhD, University of Western Ontario
Surim Son, PhD(c), University of Western Ontario
Yanina Sarquis-Adamson, PhD, Parkwood Institute
Guangyong Zou, PhD, University of Western Ontario and Robarts Research Institute
Mark Speechley, PhD, University of Western Ontario
Date: Sept 2022

1. Introduction

The SYNERGIC TRIAL (SYNchronizing Exercises, Remedies in GaIt and Cognition) is a randomized, phase II, fractional factorial, five-arm, double-blind controlled study evaluating the effect of aerobic-resistance exercise with and without cognitive training and vitamin D supplementation on cognitive function.

Specifically, physical exercise is a progressive and supervised aerobic (AE) and resistance training (RT) program tailored for older adults' physical capabilities. Computerized cognitive training will be delivered using the Neuropeak cognitive training program designed to target working memory and attention. Vitamin D supplementation will be delivered in capsules with 10,000 IU, 3 times per week, reaching a weekly dose of 30000 IU. Control interventions will be sham cognitive training, balance and toning exercise (BAT), and matching vitamin D placebo capsules.

2. Study Objectives

To evaluate:

Objective 1 The effect of supervised aerobic-resistance exercise on cognitive function

Hypothesis 1. Twenty weeks of supervised aerobic-resistance exercise will significantly improve cognitive function in older adults with MCI, as assessed by primary outcome Alzheimer's Disease Assessment Scale-Cognitive (ADAS-Cog 13 and plus variant) and secondary outcomes, compared to a balance and toning (BAT) control.

Objective 2. The effect of adding cognitive training to aerobic-resistance exercise on cognitive function.

Hypothesis 2. Adding cognitive training to aerobic-resistance exercise will significantly improve primary and secondary outcomes compared to aerobic-resistance exercise without cognitive training.

Objective 3. The effect of adding vitamin D supplementation to aerobic-resistance exercise on cognitive function.

Hypothesis 3. Adding vitamin D supplementation in weekly dose of 30000 IU to aerobic-resistance exercise will significantly improve primary and secondary outcomes compared to aerobic-resistance exercise without vitamin D supplementation.

Objective 4. The effect of multi-domain intervention (aerobic-resistance exercise + cognitive training + Vitamin D supplementation) on cognitive function.

Hypothesis 4. The multi-domain intervention (aerobic-resistance exercise + cognitive training + vitamin D supplementation) will significantly improve primary and secondary outcomes compared to the control intervention.

3. Study Population

We will target older adults aged 60 to 85 years old with mild cognitive impairment (MCI).

4. Study Design

This is a Phase II, randomized, double-blind, five-arm controlled trial. The trial design follows a fractional factorial design as shown in Figure 1. Participants will be recruited primarily from the community and from clinics serving MCI population across five sites: London (lead and sponsor site), Waterloo-University of Waterloo, Waterloo-Wilfrid Laurier University, Montreal, and Vancouver. All eligible participants will be randomized to 5 arms in 1:1:1:1:1 ratio:

Arm 1: combined AE and RT exercise + cognitive training + vitamin D

Arm 2: combined AE and RT exercise + cognitive training + placebo vitamin D

Arm 3: combined AE and RT exercise + control cognitive training + vitamin D

Arm 4: combined AE and RT exercise + control cognitive training+ placebo vitamin D

Arm 5: BAT exercise + control cognitive training + placebo vitamin D

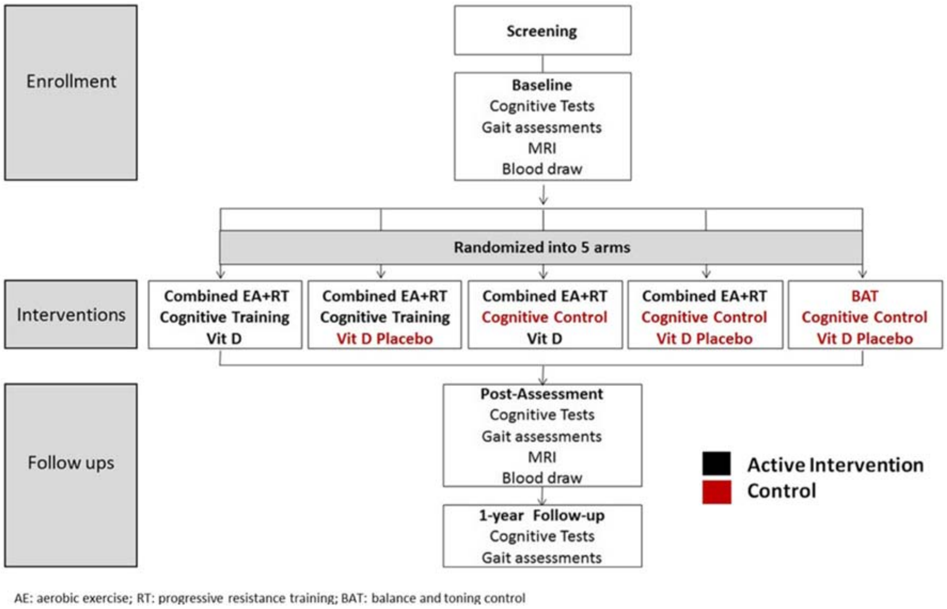

Figure 1. Trial design overview

5. Study Endpoint

The study endpoint will be at 6 Month (post-intervention).

6. Study Assessment

The participant's eligibility will be assessed during the screening visit. Assessment which will be performed during three visits: baseline, post-intervention at 6 months, and 6-month follow up after the postintervention at 12 months. The measures at each assessment are described in Table 1.

Table 1. Assessments across study visits

Procedure	Visit 1 Screening	Visit 2 Baseline	Visit 3 6 month	Telephone Follow-up at 9 month	Visit 4 12 month
Written Informed Consent	X				
Demographic Information	X				
Mini Mental State Examination (MMSE)	X				
Physical Activity Readiness Questionnaire Plus (PARQ +)	X				
Logical Memory 1 & 2	X				
CERAD Word List Recall	X				
PASE Questionnaire	X				
Montreal Cognitive Assessment (MoCA)	X		X		X
Generalized Anxiety Disorder 7 (GAD-7)	X		X		X
Geriatric Depression Scale (GDS-30)	X		X		X
Clinical Dementia Rating (CDR)	X		X		X
Activities of Daily Living (ADCS-ADL and IADL)	X		X		X
Clinical Medical Questionnaire		X	X		X
Dual Task Control Assessment		X	X		X
ADAS-Cog 13 (+ tests [a])		X	X		X
Trail Making Test A & B[a]		X	X		X
Digit Symbol Test [a]		X	X		X
Digit Span Forward and Backward WAIS-III [a]		X	X		X
Boston Naming Test [a]		X	X		X
Verbal Fluency Test [a]		X	X		X
Colour Word Interference Test		X	X		X
Quality of Life Questionnaire (SF-36)		X	X		X
Short Physical Performance Battery (SPPB)		X	X		X
Gait Assessment using Gait Mats and accelerometers (when available)		X	X		X
Six Minute Walk Test (6MWT)[b]		X	X		X
Neuroimaging (MRI)					
Blood Draw		X	X		
Falls Calendar[c]		X	X	X	X

[a]Testing included in the ADAS-Cog plus
[b]This test may be completed at the gym facility on the first day of intervention
[c]Calendar will be given to participant to complete and will be submitted to Research Staff at exercise training

7. Determination of Sample Size

Sample size calculation is based on changes in our primary outcome: global cognitive function measured using the Alzheimer Disease Assessment Scale Cognitive 13 and

the plus variant (ADAS-Cog 13 and plus). A target sample of 200 participants (40 per arm) was determined based on 80% power to detect either main effects or an interaction of at least a moderate (Cohen's d ~0.5)[1] effect size of changes in the ADAS-Cog-13.[2] The final sample size accounted for a 15% attrition.

8. Primary Outcomes

Primary outcome will be changes in cognitive function measured with the ADAS-Cog 13 items (ADAS-Cog-13) and the plus variant (ADAS-Cog-plus) at endpoint, 6 Month. Improvement at endpoint in either variant is considered evidence of efficacy.

8.1 Change in cognitive function on ADAS-Cog-13 at post-intervention (Month 6)

The ADAS-Cog 13 consists of 13 brief cognitive tests assessing memory, language, attention, concentration and praxis. The individual tests are listed in Table 2. Scores range from 0 to 84, with higher scores indicating severe cognitive impairment.

8.2 Change in cognitive function on ADAS-Cog-Plus at post-intervention (Month 6)

Four additional tests focusing on executive function will be added to the ADAS-Cog 13 to comprise the plus variant[3] (Table 2). Scores will be transformed into an algorithm validated by Crane et al.[4] The higher scores will indicate severe of cognitive impairment.

Table 2. Individual tests included in the ADAS-Cog

**ADAS-Cog-13**	**ADAS-Cog-Plus**
Word recall	Word recall
Delayed word recall	Delayed word recall
Following commands	Following commands
Constructional praxis	Constructional praxis
Ideational praxis	Ideational praxis
Naming objects	Naming objects
Orientations	Orientations
Word recognition	Word recognition
Remembering instructions	Remembering instructions
Comprehension	Comprehension
Word finding	Word finding
Spoken language	Spoken language
Number cancellation	Number cancellation
	Trial Making A and B
	Digit symbol substitution
	WAIS III digit forward and backward
	Category fluency – Animals and Vegetable

9. Secondary Outcomes*

Secondary outcomes include neuropsychological assessments, gait and mobility outcomes (including incidence of fall), neuroimaging, and blood biomarkers. *Note that secondary outcomes will be not reported in the primary manuscript.

9.1 Change in cognitive function on neuropsychological tests

The secondary cognitive outcomes will include:

a) Montreal Cognitive Assessment test for global cognition
b) The ADAS-Cog Recall word list for verbal semantic memory
c) Trail Making Test A and B
d) Digit Symbol Substitution Test
e) Digit Span Test Forward and Backwards
f) Boston Naming Test
g) Verbal fluency Animals and Vegetables

9.2 Change in gait performance

Gait performance will be evaluated using gait speed and gait variability under single and dual-tasking. Gait speed will be measured as the time taken to walk 6 m using an electronic walkway system (ProtoKinetic® and/or GAITRite® Systems, Inc.). Gait variability of spatial and temporal gait variables (stride time, stride length, double support time and step width) will be calculated using the coefficient of variation (CV = (standard deviation / mean) ×100).

9.3 Change in mobility and falls

Mobility will be measured with the Short Physical Performance Battery (SPPB), and the 6 min walk test (6MWT).

A fall will be defined as ‘unintentionally coming to rest on the ground, floor, or other lower level and not due to a seizure, syncope, or an acute stroke’. Recurrent falls are defined as ‘two or more events in a 12-month period’. Falls will be recorded throughout the 12-month trial, participants will be provided with a falls calendars, on which they will record any falls that have occurred, and they will be asked to bring them monthly to the training sessions to review with a research staff member. After the completion of the intervention, participants will be contacted at month 9 and 12 to report incidental falls.

9.4 Change in Neuroimaging

Pre-post regional patterns of brain plasticity will be assessed using structural (high-resolution 3D T1-weighted images = 7 min) in order to get voxel-based volumetric and cortical thickness measures, as well as diffusion tensor imaging (DTI, 30 directions, with

AP/PA correction scan = 7 min) to calculate fractional anisotropy and diffusivity in white matter tracts as well as derive tract-based statistics. Cerebrovascular integrity and pathology will be assessed using the following contrasts: PD/T2 = 5 min; FLAIR = 7 min, and T2* = 5 min). Functional magnetic resonance imaging at rest (rsfMRI plus field map = 12 min) will also be acquired to measure change in data-driven functional networks.

9.5 Change in Blood biomarkers measures

Serum biomarkers of inflammation (C reactive protein, and interleukin 6), neuroplasticity (brain-derived neurotropic factor), endothelial health (vascular endothelial growth factor 1), and vitamin D serum levels will be measured before and after intervention.

9.6 Covariates

Pre-specified covariates will be age, sex, years of education and number of comorbidities.

10. Statistical Analysis

10.1 Descriptive Analysis

The baseline characteristics will be summarized using means and standard deviation, or medians and interquartile range where appropriate, for continuous characteristics, and frequencies and percentages for categorical variable.

10.2 Primary Efficacy Analysis

The primary analysis will be based on the intention-to-treat principle, including everyone who were randomized. Comparisons between arms will be conducted according to pre-specifications in the protocol. These comparisons align well with the recommended analysis and reporting of factorial trials[5], which used “inside the table” and “at the margin” analyses for different comparisons. The “inside the table” analysis will be used to compare each intervention effect to control, while “at the margins” analysis will be used to assess the effect of adding intervention.

The major interest would be the effect of the intervention on change in primary outcomes at 6 Month compared to the control arm (arm 5). The primary outcome measure of mean change in ADAS-Cog-13 and ADAS-Cog-Plus scores will be analyzed using a linear mixed model with restricted likelihood estimation. The model will be fitted with participant-specific random intercept and fixed effects of time, intervention arm, and time x intervention arm interaction. The time variable will be used as a categorical variable (0 = baseline; 1 = Month 6.) The intervention arm variable will be dummy coded, using the control group as a reference category. The interaction term will be

used to assess between-group differences (representing a mean difference in change in cognitive scores over time between two groups). With dummy coding of the intervention arm, the interaction term will allow to compare the mean change in each intervention arm to the arm 5 (control). The within-group difference (representing a mean change in cognitive scores over time within the group) will be measured with marginalized mean from the linear mixed regression model. The model will be further adjusted for participant characteristics including age, sex, education, and comorbidities.

An unstructured covariance matrix will be used for all models. The model assumption will be assessed using the normal quantile plots of residuals for normality and the residuals vs. fitted plot for variance homogeneity. If the assumptions are not fully met, the data will be transformed (i.e., log). Missing data will not be imputed and will be assumed to be missing at random, as the mixed model approach is well-known to handle such data effectively.[6] The baseline data from individuals who did not complete Month 6 assessment will be included in the linear mixed model.

The mean difference between groups will be presented with their 95% CI and p-value, while the mean change within group will be presented with their standard error. The obtained marginalized mean and standard deviation will be used to calculate the effect size ($\frac{mean\ change\ in\ treatment\ X - mean\ change\ in\ treatment\ Y}{pooled\ standard\ deviation\ of\ change}$). As outlined in our published protocol, we will calculate the proportion of participants with improvement in primary outcomes.

All tests will be two-sided, and p-value of less than 0.05 will be considered as statistically significant. Adjustments for multiple comparisons will not be made as the trial is intended to compare the intervention effect to the control rather than pairwise.[7] Per-protocol analysis will be performed as well.

As per recommended analyses of fractional factorial design[8], "at the margin" analyses will be performed by collapsing arms according to the pre-specified comparison. Collapsing intervention arms allow to increase the study power to detect the difference in effect. More details are provided below for each pre-specified comparison.

10.2.1 Effect of aerobic-resistance exercise

To address objective 1 (the effect of supervised aerobic-resistance exercise on cognitive function), the intervention arms providing exercise intervention (arm 1 to 4) will be collapsed as one category and compared to the control (arm 5). The new independent variable will be created to represent exercise intervention: aerobic-resistance exercise intervention (collapsed arm 1 to 4), and control exercise intervention – BAT – (arm 5). The new intervention variable will be fitted into the same linear mixed model described above. Using the interaction between exercise intervention x time, the

change in primary outcome will be contrasted between participants receiving the aerobic-resistance exercise and those not receiving the aerobic-resistance exercise.

10.2.2 Effect of adding cognitive training

The objective 2 (the effect of adding cognitive training to aerobic-resistance exercise on cognitive function) will be examined by pooling the intervention arms providing both exercise and cognitive intervention (arm 1 and 2) and pooling the intervention arms providing exercise intervention but not cognitive intervention (arm 3 and 4). A new independent variable will be created for addition of cognitive training: both exercise and cognitive intervention (pooled arm 1 and 2) and exercise but no cognitive intervention (pooled arm 3 and 4). Using the interaction term in the linear mixed model, the contrast will be made between the two collapsed arms to assess whether the effect of adding cognitive training to aerobic-resistance exercise differ from aerobic-resistance exercise. If there is a significant difference observed, the two collapsed arms will be compared to the control arm (arm 5). This marginal analysis will allow to evaluate the main effect of aerobic-resistance exercise alone (pooled arm 1 and 2) and aerobic-resistance exercise with cognitive training (pooled arm 3 and 4) compared to the control (arm 5) with higher power (as a result of increase in sample size).[5,8]

10.2.3 Effect of adding vitamin D supplements

The objective 2 (the effect of adding vitamin D to aerobic-resistance exercise on cognitive function) will be examined by pooling the intervention arms providing both exercise and vitamin D intervention (arm 1 and 3) and pooling the intervention arms providing exercise intervention but not vitamin D (arm 2 and 4). A new independent variable will be created to represent addition of vitamin D: both aerobic-resistance exercise and vitamin D intervention (pooled arm 1 and 3) and aerobic-resistance exercise but no vitamin D intervention (pooled arm 2 and 4). The linear mixed model will be repeated using this new independent variable and the two pooled arms will be compared. This will allow to explore whether adding vitamin D to aerobic-resistance exercise improve the outcome compared to the aerobic-resistance exercise. If a significant difference is observed, the two pooled arms will be compared to the control arm (arm 5) to assess the main effect.

10.2.4 Effect of multidomain intervention

The objective 4 (effect of multi-domain intervention (aerobic-resistance exercise + cognitive training + Vitamin D supplement) on cognitive function) will be assessed using the time x intervention arm interaction fitted in the linear mixed model in section 10.2. The interaction term between time x intervention arm 1 will represents the effect of multidomain intervention (arm 1) compared to the control (arm 5).

10.2.5 Long-term effect: From Baseline and Post-intervention to Follow up

The long-term intervention effect from baseline and post-intervention to follow-up (Month 12) will be assessed using the linear mixed model. Time will be used as a categorical variable (Baseline, Month 6, and Month 12). Individuals with incomplete follow up assessments will still be included in the linear mixed model.

10.2.6 Post-hoc analyses

ADAS-Cog-Plus is a construction proposed originally by Skinner et al.[9], adding extra tests for executive function and verbal fluency to increase sensitivity to detect progression from MCI to dementia. Post hoc analyses will be performed to explore the effect of intervention on ADAS-Cog-Plus scores by adding individual Plus items, one by one, to ADAS-Cog-13.

10.3 Secondary Outcomes Analyses

The secondary analysis will be performed in an intention-to-treat population and a per-protocol population. The details of the statistical analysis plan for secondary outcomes are not included in this analysis plan as the secondary outcome analyses will not be included in this primary manuscript and performed afterwards.

11. Data management and Quality Control

All sites will use the same data collection forms to maintain consistency. Data storage and monitoring procedures will be performed using the LORIS (Longitudinal Online Research and Imaging System) system platform for uploading data specially developed for the SYNERGIC study by CCNA informatics team platform. Data quality will be assessed either using Excel and the online CCNA-LORIS platform database. All data will be entered and managed in LORIS database, and a double data entry system will be used to assure accuracy of entry. Outliers and missing data will be managed using statistical software adopted for the final analysis. Original completed CRFs will be uploaded to the media module, initial and double data entries will be performed, and arising conflicts will be resolved in the conflict resolver module. Once the conflicts are resolved, the data will be validated by the clinical team and will be ready for release through the data query tool.
See: https://www.frontiersin.org/articles/10.3389/fninf.2018.00085/full

12. Programming Plans

Data analysis will be performed using statistical software SPSS (SPSS version 23.0, SPSS Inc., Chicago, IL) and R version 3.5.1 (R Project for Statistical Computing).

References

1. Cohen J. *Statistical power analysis for the behavioral sciences.* 2nd ed. Hillsdale, N.J.: L. Erlbaum Associates; 1988.
2. Montero-Odasso M, Almeida QJ, Burhan AM, et al. SYNERGIC TRIAL (SYNchronizing Exercises, Remedies in Gait and Cognition) a multi-Centre randomized controlled double blind trial to improve gait and cognition in mild cognitive impairment. *BMC Geriatr.* 2018;18(1):93.
3. Skinner J, Carvalho JO, Potter GG, et al. The Alzheimer's Disease Assessment Scale-Cognitive-Plus (ADAS-Cog-Plus): an expansion of the ADAS-Cog to improve responsiveness in MCI. *Brain Imaging Behav.* 2012;6(4):489-501.
4. Crane PK, Carle A, Gibbons LE, et al. Development and assessment of a composite score for memory in the Alzheimer's Disease Neuroimaging Initiative (ADNI). *Brain Imaging Behav.* 2012;6(4):502-516.
5. McAlister FA, Straus SE, Sackett DL, Altman DG. Analysis and reporting of factorial trials: a systematic review. *JAMA.* 2003;289(19):2545-2553.
6. Mallinckrodt CH, Lane PW, Schnell D, Peng Y, Mancuso J. Recommendations for the primary analysis of continuous endpoints in longitudinal clinical trials. *Drug Information Journal.* 2008;42:303-319.
7. Juszczak E, Altman DG, Hopewell S, Schulz K. Reporting of Multi-Arm Parallel-Group Randomized Trials: Extension of the CONSORT 2010 Statement. *JAMA.* 2019;321(16):1610-1620.
8. Chakraborty B, Collins LM, Strecher VJ, Murphy SA. Developing multicomponent interventions using fractional factorial designs. *Stat Med.* 2009;28(21):2687-2708.
9. Skinner J, Carvalho JO, Potter GG, et al. The Alzheimer's Disease Assessment Scale-Cognitive-Plus (ADAS-Cog-Plus): an expansion of the ADAS-Cog to improve responsiveness in MCI. *Brain Imaging Behav.* 2012;6(4):489-501.

Date: 1 August 2018

To: Dr. Manuel Montero Odasso

Project ID: 107670

Study Title: SYNchronizing Exercises, Remedies in GaIt and Cognition (SYNERGIC) Trial. A Randomized Controlled Double Blind Trial

Application Type: HSREB Amendment Form

Review Type: Delegated

Meeting Date / Full Board Reporting Date: 21/Aug/2018

Date Approval Issued: 01/Aug/2018

REB Approval Expiry Date: 19/Jul/2019

Dear Dr. Manuel Montero Odasso ,

The Western University Health Sciences Research Ethics Board (HSREB) has reviewed and approved the WREM application form for the amendment, as of the date noted above.

Documents Approved:

Document Name	Document Type	Document Date
107670_31July2018	Protocol	31/Jul/2018
CCNA_LOICWestern_Waterloo_31July2018	Consent Form	31/Jul/2018
SYNERGIC Protocol_v _11 June 2018	Protocol	11/Jun/2018

Documents Acknowledged:

Document Name	Document Type	Document Date
NOL217410 _26July2018	Sponsor Correspondence	26/Jul/2018
Summary of Changes HC _ 11 June 2018	Summary of Changes	11/Jun/2018

REB members involved in the research project do not participate in the review, discussion or decision.

The Western University HSREB operates in compliance with, and is constituted in accordance with, the requirements of the TriCouncil Policy Statement: Ethical Conduct for Research Involving Humans (TCPS 2); the International Conference on Harmonisation Good Clinical Practice Consolidated Guideline (ICH GCP); Part C, Division 5 of the Food and Drug Regulations; Part 4 of the Natural Health Products Regulations; Part 3 of the Medical Devices Regulations and the provisions of the Ontario Personal Health Information Protection Act (PHIPA 2004) and its applicable regulations. The HSREB is registered with the U.S. Department of Health & Human Services under the IRB registration number IRB 00000940.

Please do not hesitate to contact us if you have any questions.

Sincerely,

Patricia Sargeant, Ethics Officer (ext. 85990) on behalf of Dr. Joseph Gilbert, HSREB Chair

Note: This correspondence includes an electronic signature (validation and approval via an online system that is compliant with all regulations).

Date: 23 November 2018

To: Dr. Manuel Montero Odasso

Project ID: 107670

Study Title: SYNchronizing Exercises, Remedies in GaIt and Cognition (SYNERGIC) Trial. A Randomized Controlled Double Blind Trial

Application Type: HSREB Amendment Form

Review Type: Delegated

Meeting Date / Full Board Reporting Date: 04/Dec/2018

Date Approval Issued: 23/Nov/2018

REB Approval Expiry Date: 19/Jul/2019

Dear Dr. Manuel Montero Odasso ,

The Western University Health Sciences Research Ethics Board (HSREB) has reviewed and approved the WREM application form for the amendment, as of the date noted above.

Documents Approved:

Document Name	Document Type	Document Date
107670_16Nov2018	Protocol	16/Nov/2018
SYNERGIC Protocol_v _16 Nov 2018	Protocol	16/Nov/2018

Documents Acknowledged:

Document Name	Document Type	Document Date
Summary of Changes _ 16 Nov 2018	Summary of Changes	16/Nov/2018

REB members involved in the research project do not participate in the review, discussion or decision.

The Western University HSREB operates in compliance with, and is constituted in accordance with, the requirements of the TriCouncil Policy Statement: Ethical Conduct for Research Involving Humans (TCPS 2); the International Conference on Harmonisation Good Clinical Practice Consolidated Guideline (ICH GCP); Part C, Division 5 of the Food and Drug Regulations; Part 4 of the Natural Health Products Regulations; Part 3 of the Medical Devices Regulations and the provisions of the Ontario Personal Health Information Protection Act (PHIPA 2004) and its applicable regulations. The HSREB is registered with the U.S. Department of Health & Human Services under the IRB registration number IRB 00000940.

Please do not hesitate to contact us if you have any questions.

Sincerely,

Patricia Sargeant, Ethics Officer (ext. 85990) on behalf of Dr. Joseph Gilbert, HSREB Chair

Note: This correspondence includes an electronic signature (validation and approval via an online system that is compliant with all regulations).

Western Research

Date: 4 March 2019

To: Dr. Manuel Montero Odasso

Project ID: 107670

Study Title: SYNchronizing Exercises, Remedies in GaIt and Cognition (SYNERGIC) Trial. A Randomized Controlled Double Blind Trial

Application Type: HSREB Amendment Form

Review Type: Delegated

Meeting Date / Full Board Reporting Date: 12/Mar/2019

Date Approval Issued: 04/Mar/2019

REB Approval Expiry Date: 19/Jul/2019

Dear Dr. Manuel Montero Odasso ,

The Western University Health Sciences Research Ethics Board (HSREB) has reviewed and approved the WREM application form for the amendment, as of the date noted above.

Documents Approved:

Document Name	Document Type	Document Date
107670_19Feb2019	Protocol	19/Feb/2019
Gait and Brain Webpage _Participant Recruitment Form	Recruitment Materials	27/Feb/2019
SYNERGIC Protocol_v _15Feb2019	Protocol	15/Feb/2019

REB members involved in the research project do not participate in the review, discussion or decision.

The Western University HSREB operates in compliance with, and is constituted in accordance with, the requirements of the TriCouncil Policy Statement: Ethical Conduct for Research Involving Humans (TCPS 2); the International Conference on Harmonisation Good Clinical Practice Consolidated Guideline (ICH GCP); Part C, Division 5 of the Food and Drug Regulations; Part 4 of the Natural Health Products Regulations; Part 3 of the Medical Devices Regulations and the provisions of the Ontario Personal Health Information Protection Act (PHIPA 2004) and its applicable regulations. The HSREB is registered with the U.S. Department of Health & Human Services under the IRB registration number IRB 00000940.

Please do not hesitate to contact us if you have any questions.

Sincerely,

Patricia Sargeant, Ethics Officer (ext. 85990) on behalf of Dr. Joseph Gilbert, HSREB Chair

Note: This correspondence includes an electronic signature (validation and approval via an online system that is compliant with all regulations).

Date: 18 October 2019

To: Dr. Manuel Montero Odasso

Project ID: 107670

Study Title: SYNchronizing Exercises, Remedies in GaIt and Cognition (SYNERGIC) Trial. A Randomized Controlled Double Blind Trial

Application Type: HSREB Amendment Form

Review Type: Delegated

Meeting Date / Full Board Reporting Date: 05/Nov/2019

Date Approval Issued: 18/Oct/2019

REB Approval Expiry Date: 19/Jul/2020

Dear Dr. Manuel Montero Odasso ,

The Western University Health Sciences Research Ethics Board (HSREB) has reviewed and approved the WREM application form for the amendment, as of the date noted above.

Documents Approved:

Document Name	Document Type	Document Date	Document Version
Synergic Radio Advertisement 570News UW Site_Oct2019	Recruitment Materials	18/Oct/2019	1

REB members involved in the research project do not participate in the review, discussion or decision.

The Western University HSREB operates in compliance with, and is constituted in accordance with, the requirements of the TriCouncil Policy Statement: Ethical Conduct for Research Involving Humans (TCPS 2); the International Conference on Harmonisation Good Clinical Practice Consolidated Guideline (ICH GCP); Part C, Division 5 of the Food and Drug Regulations; Part 4 of the Natural Health Products Regulations; Part 3 of the Medical Devices Regulations and the provisions of the Ontario Personal Health Information Protection Act (PHIPA 2004) and its applicable regulations. The HSREB is registered with the U.S. Department of Health & Human Services under the IRB registration number IRB 00000940.

Please do not hesitate to contact us if you have any questions.

Sincerely,

Patricia Sargeant, Ethics Officer (ext. 85990) on behalf of Dr. Joseph Gilbert, HSREB Chair

Note: This correspondence includes an electronic signature (validation and approval via an online system that is compliant with all regulations).

Date: 30 October 2019

To: Dr. Manuel Montero Odasso

Project ID: 107670

Study Title: SYNchronizing Exercises, Remedies in GaIt and Cognition (SYNERGIC) Trial. A Randomized Controlled Double Blind Trial

Application Type: HSREB Amendment Form

Review Type: Delegated

Meeting Date / Full Board Reporting Date: 19/Nov/2019

Date Approval Issued: 30/Oct/2019

REB Approval Expiry Date: 19/Jul/2020

Dear Dr. Manuel Montero Odasso ,

The Western University Health Sciences Research Ethics Board (HSREB) has reviewed and approved the WREM application form for the amendment, as of the date noted above.

Documents Approved:

Document Name	Document Type	Document Date	Document Version
107670_28Oct2019_Clean	Summary of Changes	28/Oct/2019	2
Gait&Brain Brochure_Oct2019	Recruitment Materials	28/Oct/2019	1
SYNERGIC Protocol _18Oct2019_Clean	Protocol	18/Oct/2019	1

Documents Acknowledged:

Document Name	Document Type	Document Date	Document Version
Summary of Changes_18Oct2019	Summary of Changes	18/Oct/2019	1

REB members involved in the research project do not participate in the review, discussion or decision.

The Western University HSREB operates in compliance with, and is constituted in accordance with, the requirements of the TriCouncil Policy Statement: Ethical Conduct for Research Involving Humans (TCPS 2); the International Conference on Harmonisation Good Clinical Practice Consolidated Guideline (ICH GCP); Part C, Division 5 of the Food and Drug Regulations; Part 4 of the Natural Health Products Regulations; Part 3 of the Medical Devices Regulations and the provisions of the Ontario Personal Health Information Protection Act (PHIPA 2004) and its applicable regulations. The HSREB is registered with the U.S. Department of Health & Human Services under the IRB registration number IRB 00000940.

Please do not hesitate to contact us if you have any questions.

Sincerely,

Patricia Sargeant, Ethics Officer (ext. 85990) on behalf of Dr. Joseph Gilbert, HSREB Chair

Note: This correspondence includes an electronic signature (validation and approval via an online system that is compliant with all regulations).

Date: 15 April 2020

To: Dr. Manuel Montero Odasso

Project ID: 107670

Study Title: SYNchronizing Exercises, Remedies in GaIt and Cognition (SYNERGIC) Trial. A Randomized Controlled Double Blind Trial

Application Type: HSREB Amendment Form

Review Type: Delegated

Meeting Date / Full Board Reporting Date: 21/Apr/2020

Date Approval Issued: 15/Apr/2020

REB Approval Expiry Date: 19/Jul/2020

Dear Dr. Manuel Montero Odasso ,

The Western University Health Sciences Research Ethics Board (HSREB) has reviewed and approved the WREM application form for the amendment, as of the date noted above. **However, the HSREB has concerns regarding the shift in study procedures affecting the objectives of the study. The HSREB is concerned about the ability for the study team to execute the new study procedures with this participant population, and the potential for the transmission of the virus during the COVID-19 precautions. Approval for this amendment has been granted based on the study team's efforts to mitigate the concerns of the HSREB. The HSREB would like a report of the progress of this amendment (via a Reportable Event-FYI or Correspondence via WREM) by June 15, 2020.**

Documents Approved:

Document Name	Document Type	Document Date	Document Version
107670_14April2020_Clean	Protocol	14/Apr/2020	
CCNA_LOICAdd_UWO_UWW_14April2020	Consent Form	14/Apr/2020	2
Concurrent Call Script_7April2020	Paper Survey	07/Apr/2020	
SYN Take Home Program (Group 1-2)_3Apr2020	Other Materials	03/Apr/2020	
SYN Take Home Program (Group 3-4)_3Apr2020	Other Materials	03/Apr/2020	
SYN Take Home Program (Group 5)_3Apr2020	Other Materials	03/Apr/2020	
Take Home Worksheet (Group5)_3April2020	Other Materials	03/Apr/2020	
Take Home Worksheet (Groups1-2)_3April2020	Other Materials	03/Apr/2020	
Take Home Worksheet (Groups3-4)_3April2020	Other Materials	03/Apr/2020	

Documents Acknowledged:

Document Name	Document Type	Document Date
Summary of Changes_7April2020	Summary of Changes	07/Apr/2020

REB members involved in the research project do not participate in the review, discussion or decision.

The Western University HSREB operates in compliance with, and is constituted in accordance with, the requirements of the TriCouncil Policy Statement: Ethical Conduct for Research Involving Humans (TCPS 2); the International Conference on Harmonisation Good Clinical Practice Consolidated Guideline (ICH GCP); Part C, Division 5 of the Food and Drug Regulations; Part 4 of the Natural Health Products Regulations; Part 3 of the Medical Devices Regulations and the provisions of the Ontario Personal Health Information Protection Act (PHIPA 2004) and its applicable regulations. The HSREB is registered with the U.S. Department of Health & Human Services under the IRB registration number IRB 00000940.

Please do not hesitate to contact us if you have any questions.

Sincerely,

Patrica Sargeant, Ethics Officer (psargean@uwo.ca) on behalf of Dr. Joseph Gilbert, HSREB Chair

Note: This correspondence includes an electronic signature (validation and approval via an online system that is compliant with all regulations).

Date: 14 May 2020

To: Dr. Manuel Montero Odasso

Project ID: 107670

Study Title: SYNchronizing Exercises, Remedies in GaIt and Cognition (SYNERGIC) Trial. A Randomized Controlled Double Blind Trial

Application Type: HSREB Amendment Form

Review Type: Delegated

Meeting Date / Full Board Reporting Date: 02/Jun/2020

Date Approval Issued: 14/May/2020

REB Approval Expiry Date: 19/Jul/2020

Dear Dr. Manuel Montero Odasso ,

The Western University Health Sciences Research Ethics Board (HSREB) has reviewed and approved the WREM application form for the amendment, as of the date noted above.

Documents Approved:

Document Name	Document Type	Document Date	Document Version
107670_13May2020_Clean	Protocol	13/May/2020	1
CCNA_LOICAdd_UWO_UWW_14May2020	Consent Form	14/May/2020	1

Documents Acknowledged:

Document Name	Document Type	Document Date	Document Version
Summary of Changes_14May2020	Summary of Changes	14/May/2020	1

REB members involved in the research project do not participate in the review, discussion or decision.

The Western University HSREB operates in compliance with, and is constituted in accordance with, the requirements of the TriCouncil Policy Statement: Ethical Conduct for Research Involving Humans (TCPS 2); the International Conference on Harmonisation Good Clinical Practice Consolidated Guideline (ICH GCP); Part C, Division 5 of the Food and Drug Regulations; Part 4 of the Natural Health Products Regulations; Part 3 of the Medical Devices Regulations and the provisions of the Ontario Personal Health Information Protection Act (PHIPA 2004) and its applicable regulations. The HSREB is registered with the U.S. Department of Health & Human Services under the IRB registration number IRB 00000940.

Please do not hesitate to contact us if you have any questions.

Sincerely,

Patricia Sargeant, Ethics Officer (psargean@uwo.ca) on behalf of Dr. Joseph Gilbert, HSREB Chair

Note: This correspondence includes an electronic signature (validation and approval via an online system that is compliant with all regulations).

Western Research

Date: 12 June 2020

To: Dr. Manuel Montero Odasso

Project ID: 107670

Study Title: SYNchronizing Exercises, Remedies in GaIt and Cognition (SYNERGIC) Trial. A Randomized Controlled Double Blind Trial

Application Type: HSREB Amendment Form

Review Type: Delegated

Meeting Date / Full Board Reporting Date: 07/Jul/2020

Date Approval Issued: 12/Jun/2020

REB Approval Expiry Date: 19/Jul/2020

Dear Dr. Manuel Montero Odasso ,

The Western University Health Sciences Research Ethics Board (HSREB) has reviewed and approved the WREM application form for the amendment, as of the date noted above.

Documents Approved:

Document Name	Document Type	Document Date
107670_8June2020_Clean	Protocol	08/Jun/2020

Documents Acknowledged:

Document Name	Document Type	Document Date
Summary of Changes_8June2020	Summary of Changes	08/Jun/2020

REB members involved in the research project do not participate in the review, discussion or decision.

The Western University HSREB operates in compliance with, and is constituted in accordance with, the requirements of the TriCouncil Policy Statement: Ethical Conduct for Research Involving Humans (TCPS 2); the International Conference on Harmonisation Good Clinical Practice Consolidated Guideline (ICH GCP); Part C, Division 5 of the Food and Drug Regulations; Part 4 of the Natural Health Products Regulations; Part 3 of the Medical Devices Regulations and the provisions of the Ontario Personal Health Information Protection Act (PHIPA 2004) and its applicable regulations. The HSREB is registered with the U.S. Department of Health & Human Services under the IRB registration number IRB 00000940.

Please do not hesitate to contact us if you have any questions.

Sincerely,

Patricia Sargeant, Ethics Officer (psargean@uwo.ca) on behalf of Dr. Joseph Gilbert, HSREB Chair

Note: This correspondence includes an electronic signature (validation and approval via an online system that is compliant with all regulations).

Summary of Changes Document
SYNERGIC Trial # 201619

Proposed Changes	Rationale
**Imaging**<br>Spectroscopy	The rationale for spectroscopy is related that we have previously found that NAA/Cr levels in the motor cortex are associated with vitamin D status (Annweiler et. al. Vitamin D and Caudal Primary Motor Cortex: A Magnetic Resonance Spectroscopy Study, Plos One, 9: e87314, 2014) in MCI. In addition, we have shown that NAA/Cr levels in the motor cortex are associated with worse stride time variability under dual task conditions (Annweiler et al. Motor Cortex and Gait in Mild Cognitive Impairment: A Magnetic Resonance Spectroscopy and Volumetric Imaging Study. Brain. 136: 859-871, 2013).<br><br>These studies suggest that NAA/Cr levels are associated with reduced vitamin D levels, and gait disturbance. These studies provide the rational to examine the effect of exercise training and vitamin D supplementation (the focus of SYNERGIC) in a similar cohort on this metabolic indicator in the motor cortex. This metabolic measurement may be a more sensitive indicator than brain volumetrics and tissue microstructure (DTI) to exercise and vitamin D supplementation induced changes - although there is also evidence to support the use of these other measures in the current study.
**Blood work**<br>Blood draw added at T12 (final) study visit.	Blood work is completed with participants currently at Baseline and also after intervention completes at their 6-month visit. This amendment proposes adding blood work to the final visit (12-month visit) as a follow-up. This blood work will help to explore the role of exercise and cognitive training on biomarker levels and if there is any sustained effect of exercise or interventions on a deeper (biological) level.
**Site Recruitment**<br>Distribution of enrolled participants amended.	The total enrolment will remain at 200 participants. 10 participant spots from the Montreal site will be transferred to the London site (Parkwood Institute). 10 participant spots will be transferred from the Vancouver site and split evenly to the London (5) and Waterloo (5) site. This change is being implemented as the London and Waterloo sites since these sites are reaching trial milestones.

Summary of Changes Document
SYNERGIC Trial # 201619

Proposed Changes	Rationale
**Blood Biomarkers**<br>IGF-1 to be added to blood work analysis.	IGF-1 is a biomarker that has recently been associated with poor cognitive response to exercises and with pathologies that can affect vascular and brain health, like the metabolic syndrome. Adding IGF1 in this trial will help our researchers to determine profiles of responders to the exercise and cognitive training intervention, among our participants. Blood work is approved to be collected at baseline, T6 (post-intervention) and T12 (six months post-intervention). Other biomarkers and analysis currently examined are Vitamin D levels, Calcium, PTH, Lipid levels Serum High Sensitive CRP, IL-6, BDNF, and VEGF receptor 1. This addition will have no impact on the participants as blood work previously collected will be utilized and no additional draw will occur.
**Site Recruitment**<br>Distribution of enrolled participants amended.	The total enrolment will remain at 200 participants. 5 participant spots from the Montreal site will be transferred to the London site (Parkwood Institute). 5 participant spots will be transferred from the Vancouver site to the London. This change is being implemented as the London site is reaching trial milestones.

Summary of Changes Document
SYNERGIC Trial # 201619

Proposed Changes	Rationale
**Webpage Recruitment**<br>Addition of form for interested participants.	For interested participants who visit the Gait and Brain webpage, a form will be available for them to complete in order to be contacted regarding the study and possible screening visit.
**Site Recruitment**<br>Distribution of enrolled participants amended.	The total enrolment will remain at 200 participants. 5 participant spots from the Montreal site will be transferred to the London site (Parkwood Institute). This change will be implemented as the London site is reaching trial milestones.

Summary of Changes Document
SYNERGIC Trial # 201619

Proposed Changes	Rationale
**Recruitment Materials**<br>Gait & Brain Lab Brochure	For interested participants, a brochure will be available for them to read more about the ongoing research studies in the Gait & Brain Lab, including the Synergic Trial. If interested, participants can then contact the research coordinator.
**Protocol Document: Non-Local Site Recruitment**<br>Distribution of enrolled participants amended.	The total enrolment will remain at 200 participants. 5 participant spots from the Montreal site will be transferred to the Wilfred Laurier (WLU) site. This change will be implemented as the WLU site is reaching trial milestones.

CLIENT: **University of Waterloo**	REP: Patricia Azevedo
LENGTH: 30 seconds	DATE: 10/15/2019
WRITER: Ian Hunter	TITLE: Trial Study
CART#: **CSO-**	RUN DATE: **10/21 – 10/27**
PROD INSTRUCTIONS: Cold voice, no music. MP3 to Patricia.	

"Are you 60 years of age or older?

The University of Waterloo Brain and Body Lab is hosting a clinical trial for participants who are experiencing memory problems.

This five-month trial by the Brain and Body Lab focuses on the effects of physical exercise, mental exercise and vitamin D.

To learn more about this research study, contact Laura Middleton at the University of Waterloo or visit Synergic Trial dot com.

That's S-Y-N-E-R-G-I-C Trial dot com."

© This script is property of Rogers Communications Inc

Summary of Changes Document
SYNERGIC Trial # 107670

Proposed Changes	Rationale
**Changes to study procedures, design**<br>Home-Based Program	In line with global efforts to mitigate the spread of the COVID-19 pandemic, we have temporarily suspended the SYNERGIC Trial. This decision has been made with careful consideration for the well-being of our participants who are classified as the high-risk population, and the inability to deliver safe exercise to this population while maintaining physical distance measures. In line with this decision, all interventional sites began limiting access from an institutional-level.<br>Due to institutional closures, in-gym exercise sessions are no longer possible. For participants who had not yet completed 80% of interventional sessions (therefore no T6 could be done), they will be offered a take-home maintenance program. This applies to 1 participant at University of Waterloo site. This program will provide continuation of cognitive and exercise training, and vitamin D supplementation until the 20 weeks of intervention has been reached or until institutional closures cease. To maintain participant supervision in the study, research staff will contact participants 3 times weekly on days that would normally be interventional days. With participant consent, these will be a Zoom video call during intervention, where staff have the ability to supervise technique and provide insight into adaptations/progressions where applicable. If video calls are not available, telephone calls will be completed instead.
**Changes to participant tools/study instruments**<br>Addition of new study data collection tools/forms	Calls during interventional visits will follow a call script. This script includes data collection of vitamin D supplementation, cognitive training information, and exercise training information for each session. This will also feature any questions the participant may have regarding their tasks, any reported pain or discomfort, and any modifications suggested by the researcher.
Addition of new tools/forms to be provided to participants	Participants will receive all necessary materials to continue home-based cognitive, exercise, and vitamin D intervention. This includes iPads, training manual, worksheets, vitamin D, and resistance bands where necessary (if participant does not have their own). Documents will be sent electronically. A drop-off will be arranged by the Research Coordinator with the participant for physical materials to ensure quick and sanitary delivery. Proper sanitization of materials will be conducted by the RC prior to packaging in a box and hand hygiene will be adhered to when delivering. This box will then be delivered to an agreed upon location with proper physical distancing.

	Training manual and worksheets will be specific to randomization arm. Arms 1 & 2 will receive a training manual featuring exercises that can be done at home with minimal/no equipment which target the same muscle groups as the gym-based intervention. These exercises have 3 options for progression. Starting level and progressions for each participant will be made by the researcher supervising via Zoom using input from the participant and observations. This training manual also features all warm-up and cool-down exercises, and information on cardiovascular exercises that can be completed at home. This training manual will also have information on how to access Neuropeak cognitive training at home. The training manual for arms 3-4 will be the same as that for arms 1-2, with the exception of the cognitive training section. This section will instead feature information on how to complete the control cognitive tasks at home. The worksheets participants would normally receive for cognitive control training will be provided, as well as the lists of places and informational videos (with links) that they have not yet completed during intervention. The training manual for arm 5 will feature the same cognitive training section as arms 3-4, and warm-up and cool-down stretches will remain consistent across all groups. Arm 5's exercise training will feature light balance and toning (BAT) exercises they can complete at home. Participants will receive a training worksheet specific to their grouping, on which they can log vitamin D intake, cognitive training, and exercise training for the day, and note any questions they want to ask the research staff or reminders for themselves.
**Changes to how data is collected/accessed**	Intervention information will be collected via Zoom video call during intervention and recorded on the concurrent call script/sheet. Storage of information will not change. The participants will also record their intervention information.<br><br>Should the participants reach the end of their interventional sessions (20 weeks total) prior to reversal of institutional closures, the 6-month assessment will be collected via Zoom to avoid loss of participant data. The assessment will be abbreviated as gait, balance, blood work, and MRI will not be collected, however all cognitive testing and questionnaires will be collected as usual. No new assessment materials will be used. The assessor will indicate all responses possible on hard-copy versions (the storage of which will not change). For assessments that require a paper-based copy for participant use, the required response sheets will be sent electronically to participants to be printed ahead of time (without instructions so as to not allow training). During the assessment, the participant will be asked to point the camera towards their response sheet

	and a screenshot of the response sheet will be taken by the researcher. The participant will not be in this frame. Screenshots will be saved in a secure drive. Following assessments, these screenshots will be printed and included in the participant's study folder. Any identifiable information within the screenshot will be removed accordingly. All cognitive tests will be done via Zoom, however participants will have the choice to complete questionnaires in a separate telephone call.
**Changes to the Letter of Information/Consent**	LOIC addendum document will be presented to participants currently in intervention to gain consent for participation in home-based program, regular video call supervision, and 6-month post-intervention video call assessment. LOIC addendum will be sent electronically to participants in intervention, reviewed using a telephone call with Research Coordinator, signed by participant if consenting, and a scanned copy will be sent back to the Research Coordinator. As these updates apply only to those currently in intervention, the updated LOIC will not be presented to past participants or participants who are currently in the follow-up period at this time.

Summary of Changes Document
SYNERGIC Trial # 107670

Proposed Changes	Rationale
**Changes to study procedures, design (Visit 4)**	Due to gradual opening of institutions and the staggered nature of these openings between London and Waterloo, the T12 assessment will be offered in the currently approved format (virtual) and the previously approved format (in-person). In-person assessments will be done when possible, but virtual formats will continue to be implemented a) at sites that have not yet opened for in-person appointments, and b) in the case where participants do not yet feel comfortable returning to the lab.

Summary of Changes Document
SYNERGIC Trial # 107670

Proposed Changes	Rationale
**Changes to study procedures, design (Visit 4)**	Due to continuing restrictions regarding in-person research, participants who are due for a T12 assessment prior to the return to normal operations will be invited to complete a 12-month assessment over Zoom. The assessment will mimic the current virtual T6 assessment occurring for this study, and will be abbreviated as gait, balance, blood work, and MRI will not be collected, however all cognitive testing and questionnaires will be collected as usual. No new assessment materials will be used. The assessor will indicate all responses possible on hard-copy versions (the storage of which will not change). For assessments that require a paper-based copy for participant use, the required response sheets will be sent electronically to participants to be printed ahead of time (without instructions so as to not allow training). During the assessment, the participant will be asked to point the camera towards their response sheet and a screenshot of the response sheet will be taken by the researcher. The participant will not be in this frame. Screenshots will be saved in a secure drive. Following assessments, these screenshots will be printed and included in the participant's study folder. Any identifiable information within the screenshot will be removed accordingly. All cognitive tests will be done via Zoom, however participants will have the choice to complete questionnaires in a separate telephone call.
**Changes to the Letter of Information/Consent**	LOIC addendum document will be presented to participants currently in post-intervention follow up period to gain consent for participation in 12-month video call assessment. LOIC addendum will be sent electronically to participants in intervention, reviewed using a telephone call with Research Coordinator, signed by participant if consenting, and a scanned copy will be sent back to the Research Coordinator. As these updates apply only to those currently in post-intervention follow up period, the updated LOIC will not be presented to past participants.
