# Supplementary material for: Effects of Exercise Alone or Combined With Cognitive Training and Vitamin D Supplementation to Improve Cognition in Adults With Mild Cognitive Impairment: A Randomized Clinical Trial
Source: JAMA Netw Open. 2023 Jul 20;6(7):e2324465. doi: 10.1001/jamanetworkopen.2023.24465 (PMC10359965; doi:10.1001/jamanetworkopen.2023.24465)
Supplement: Supplement 2. — eMethods. eTable 1. Details of the Participants Who Were Withdrawn From the SYNERGIC Trial Due to Medical Reasons eTable 2. The Effect of Exercise, With (Arm 1+2) and Without (Arm 3+4) Cognitive Intervention on ADAS-Cog 13 Scores at 6-Month End Point, Compared With Control (Arm 5), and Adjusted for Age, Sex, Education, and Comorbidities eTable 3. Within-Group and Between-Group Intervention Effect Across 5 Intervention Arms at 6-Month End Point eTable 4. Within-Group and Between-Group Intervention Effect Across 5 Intervention Arms at 6-Month End Point, Adjusted for Age, Sex, Education, and Comorbidities eTable 5. Number of Participants With Improvement in ADAS-Cog-13 and Plus Variant at 6 Months by Intervention Arm eTable 6. Per-Protocol Analysis of the Effect of Within-Group and Between-Group Intervention Across 5 Intervention Arms at 6-Month End Point eTable 7. Per-Protocol Analysis for the Effect of Exercise Intervention With Addition of Cognitive Training and Vitamin D at 6-Month End Point eTable 8. Per-Protocol Analysis for the Effect of Exercise and Cognitive Intervention and Exercise Alone at 6-Month End Point eTable 9. The Effect of Intervention on ADAS-Cog-Plus Scores at 6-Month End Point Calculated by Cumulatively Adding Individual Plus Items eTable 10. The Effect of Exercise, With (Arm 1+2) and Without (Arm 3+4) Cognitive Intervention on ADAS-Cog-Plus Scores at 6-Month End Point Compared With Control (Arm 5), Calculated by Cumulatively Adding Individual Plus Items eTable 11. Change in ADAS-Cog-13 and ADAS-Cog-Plus at 12-Month Follow-up eFigure 1. Change in ADAS-Cog-13 Scores From Baseline to 12 Months Across 5 Arms eTable 12. The Effect of Exercise, With (Arm 1+2) and Without (Arm 3+4) Cognitive Intervention at 12-Month Follow-up, Compared With Control (Arm 5) Using “At the Margin” Analysis eFigure 2. Change in ADAS-Cog-13 Scores From Baseline to 12 Months eTable 13. Exercise and Physical Activity Level Reported at Month 12 eTable 14. Self-Reported Adverse [file jamanetwopen-e2324465-s002.pdf]

Supplemental Online Content

Montero-Odasso M, Zou G, Speechley M, et al; Canadian Gait and Cognition Network. Effects of exercise alone or combined with cognitive training and vitamin D supplementation to improve cognition in adults with mild cognitive impairment: a randomized clinical trial. *JAMA Netw Open.* 2023;6(7):e2324465. doi:10.1001/jamanetworkopen.2023.24465

eMethods.

eTable 1. Details of the Participants Who Were Withdrawn From the SYNERGIC Trial Due to Medical Reasons

eTable 2. The Effect of Exercise, With (Arm 1+2) and Without (Arm 3+4) Cognitive Intervention on ADAS-Cog 13 Scores at 6-Month End Point, Compared With Control (Arm 5), and Adjusted for Age, Sex, Education, and Comorbidities

eTable 3. Within-Group and Between-Group Intervention Effect Across 5 Intervention Arms at 6-Month End Point

eTable 4. Within-Group and Between-Group Intervention Effect Across 5 Intervention Arms at 6-Month End Point, Adjusted for Age, Sex, Education, and Comorbidities

eTable 5. Number of Participants With Improvement in ADAS-Cog-13 and Plus Variant at 6 Months by Intervention Arm

eTable 6. Per-Protocol Analysis of the Effect of Within-Group and Between-Group Intervention Across 5 Intervention Arms at 6-Month End Point

eTable 7. Per-Protocol Analysis for the Effect of Exercise Intervention With Addition of Cognitive Training and Vitamin D at 6-Month End Point

eTable 8. Per-Protocol Analysis for the Effect of Exercise and Cognitive Intervention and Exercise Alone at 6-Month End Point

eTable 9. The Effect of Intervention on ADAS-Cog-Plus Scores at 6-Month End Point Calculated by Cumulatively Adding Individual Plus Items

eTable 10. The Effect of Exercise, With (Arm 1+2) and Without (Arm 3+4) Cognitive Intervention on ADAS-Cog-Plus Scores at 6-Month End Point Compared With Control (Arm 5), Calculated by Cumulatively Adding Individual Plus Items

eTable 11. Change in ADAS-Cog-13 and ADAS-Cog-Plus at 12-Month Follow-up

eFigure 1. Change in ADAS-Cog-13 Scores From Baseline to 12 Months Across 5 Arms

eTable 12. The Effect of Exercise, With (Arm 1+2) and Without (Arm 3+4) Cognitive Intervention at 12-Month Follow-up, Compared With Control (Arm 5) Using "At the Margin" Analysis

eFigure 2. Change in ADAS-Cog-13 Scores From Baseline to 12 Months

eTable 13. Exercise and Physical Activity Level Reported at Month 12

eTable 14. Self-Reported Adverse Events and Health Care Follow-up Diagnosis During the Study

eReferences.

This supplemental material has been provided by the authors to give readers additional information about their work.

© 2023 Montero-Odasso M et al. *JAMA Network Open.*

eMethods.

MCI Alberta criteria[1] and operationalization in the SYNERGIC trial

Criterion	Operationalization
i) Subjective cognitive complains	Self-reported and/or by an informant
ii) Objective cognitive impairment in **1** of the following 4 cognitive domains: memory, executive function, attention, and language	1 or more of the following:<br>- Montreal Cognitive Assessment (MoCA) scores ranging from: 13-24/30<br>- Logical Memory below Alzheimer's Disease Neuroimaging Initiative (ADNI) cut-offs: <9 for 16+ years of education; <5 for 8-15 years of education; <3 for 0-7 years of education.<br>- Consortium to Establish a Registry for Alzheimer's Disease (CERAD) word list recall < 6.
iii) Preserved activities of daily living	Score >14/23 on the Lawton-Brody Instrumental Activities Of Daily Living (IADL)
iv) Absence of dementia	- Diagnostic and Statistical Manual of Mental Disorders (V Edition)<br>and/or<br>- Global Clinical Dementia Rating (CDR) ≤ 0.5

Details of Cognitive training intervention

Cognitive Training (CT) intervention will involve tablet-based multimodal and multi-domain dual-task training with memory load. The training uses the custom-written program, developed for neuro- rehabilitation and used in previous research trials for cognitive[2-4] and mobility outcomes.[5] Training sessions will take place in groups of up to eight participants before each of the fitness-training session for duration of 30 min max. Participants will perform a concurrent visuo-motor task (dual-task combination) composed of different sets of visual stimuli that have to be identified by tapping designated figures on an iPad (or an Android tablet). Participants will perform discrimination tasks involving sets of items (e.g. letters, numbers, animals, vehicles, fruits, celestial bodies). Each session will involve two sets of items, one for each task. The combination of sets of items will change every four sessions.

Task instructions will always be administered by a trained Research Assistant. Within each training session and for each task combination, participants will complete a first block of single-pure (SP) trials followed by mixed-trial blocks with single-task trials (SM) intermixed and dual-mixed trials (DM: 2 tasks at once). Each training session will involve 80 SP, 128 SM and 192 DM trials (thus 40% of the DM blocks will be SM trials). Within the DM trials, the first 30 training sessions will be performed following a fixed priority instruction (participants will be asked to keep an equal priority on left and right hands.) The following 30 sessions will be performed following a variable priority instruction (participants will be told to vary priority from one hand to another in different blocks). Participants will complete a total of 60 sessions. Importantly, an adaptive continuous feedback on performance was will be provided during the dual-mixed block of the training sessions. Feedback will take the shape of a speedometer whose indicator moves and changes colour (green, yellow, orange or red) to inform participants of their response speed. Two speedometers (one for each hand) will be displayed at the top of the screen. Each speedometer is associated to one task. In equal priority blocks, participants will be asked to try to maintain both speedometers at least in the yellow zone and prevent them from turning to red. In variable priority blocks, participants will be asked to maintain the speedometer associated to the prioritized task in the green zone, while the other speedometer can be at least in the orange zone. The colour of the speedometers will be determined by the average reaction time (RT) on the last three trials' RT for the DM block compared to the median RT for the SM trials multiplied by a factor of 1.5 (for the first training session, as there are no previous

SM trials, the DM block will be compared to 1500ms). In addition, a feedback will be provided at the end of the session, where participants will be informed of mean RT and accuracy achieved throughout sessions (presented in a histogram without explicit values).

Details of Control Cognitive training

The active control cognitive training group will participate in skills training courses based on a published study from our research group.[6] Each session will consist of Internet search for 3 hotels, 3 touristic places and 3 restaurants, of participant's preference, in cities determined by the instructor at the beginning of each training session (a list of cities is presented in the manual of procedures for cognitive training). Another task will be watching a 20 minutes National Geographic documentary movie selected by the instructor at the beginning of each session; at the end of the movie participants need to answer three questions about the watched movie. These iPad tasks will be alternated between sessions to prevent boredom. The approximate time to perform both tasks is 20 minutes which is the approximate time to complete the dual-task training on the iPad.

Active control sessions will be held in groups of up to 8 participants and will take place in the same context and physical environment used for dual-task training sessions and lead by the same instructors. Studies compared dual-task training to single-task training, as a control condition, in order to control for exposure of the same specific tasks performed separately.[6] However, it can be argued that single- task training is not engaging or stimulating enough and does not provide a feeling of progression. In response to this, it was recommended that cognitive training studies use preferably sham trainings whereby clinician interaction and participant expectation effects can be reliably matched. Therefore, this study will compare dual-task training to a non-specific intervention, more comparable to cognitively stimulating activities that older adults can naturally engage in. To assure same time exposure and same social interaction than in active intervention, the control intervention will be held in groups using the same type of device that is used for the active intervention.

Details of Exercise intervention

The exercises training program involves 3 weekly sessions of approximately 60 min each. All sessions will be held in appropriate gym facilities and take place between Monday and Friday ensuring that there are not three consecutive days (i.e. training can occur on Monday, Wednesday and Friday or Monday, Thursday and Friday etc.). Staff with experience and/or certification in exercises (graduate student in kinesiology, Canadian Society of Exercise Physiology certificates, etc.) will supervise all training sessions with at least one coach per four participants.

After a general warm-up, participants will execute the strength-training portion by performing light strengthening exercises (pushes and pulls using elastic bands, chair stands, and forward lunges). The next phase will include the following exercises: leg press, leg flexion (lower body), seated press, seated row, and seated pull (upper body). These exercises are made in a circuit alternating between lower and upper body exercises. A list of suggested exercises base on muscle groups will be provided to the trainers. The recovery period between exercises corresponds to the time needed to move from one station to another. While the first portion of this training protocol (12 weeks) will be dedicated to strength endurance exercises the following 12 weeks will focus on maximal strength. Training volume and intensity are described in the table below. Sets represent the number of circuit rounds while repetitions are maximal, which means that the resistance must be adjusted by the trainer so that the participant reaches exhaustion at the last prescribed repetition of each set. Training prescription for all exercises is made in accordance to the American College of Sport Medicine (ACSM) guidelines for strength development in older adults (ACSM, version 1998).

Weeks	Sets	Repetitions	Rest between sets (sec)

© 2023 Montero-Odasso M et al. *JAMA Network Open.*

	Day 1	Day 2	Day 3	Day 1	Day 2	Day 3	
1-4	2	2	2	15-18	15-18	15-18	30
5-8	3	2	3	12-15	12-15	12-15	30
9-12	3	2	3	10	10	10	60
13-16	2	3	2	8	12	8	60
17-20	3	3	4	6	8	6	60

Following the strength portion of each session, an aerobic training will be prescribed. Participants will be asked to complete 10-20 minutes of cardiovascular exercises using different ergometers (treadmill, elliptical machines, cycling ergometers, rowing machines etc.), using steps or other forms of free aerobic exercises, as long as the cognitive load is minimized. Intensity will be monitored using a Borg scale (0-10) and the target will correspond to 5-6 for the first month, 6-7 for the second month and 7-8 for the last month. Details of the aerobic training are presented in the table below. Each session will end with a five (5) minute recovery period, which will consist of different breathing exercises and stretching.

Weeks	Sets	Duration (min)	Intensity (Borg 0-10)	Rest between sets (minutes)
1-4	2	10	5-6	1
5-8	2	10	5-6	1
9-12	2	10	6-7	1
13-16	2	10	6-7	1
17-20	2	10	7-8	1

Overall rate of perceived exertion (RPE) will be monitored using a Borg scale (0-10) 30 minutes after the end of each session. Participants will have to rate the overall difficulty of the training session. These results will provide information about the training load (session RPE – sRPE), monotony and strain.

Details of Control Exercise (Tone-Stretching Exercises)

Participants assigned in the control exercise condition will take part in tone-stretching sessions in groups of up to 8 participants, supervised by a trainer. The exercises will be devoted to improve muscle tone, flexibility, without improving, strength, cardiorespiratory capacity (and mobility). Resistant load and number of repetitions will not progress across exercise sessions. The session will start with a 5-minute warm-up on the machine of their choice. This will be followed by 50 minutes of stretching exercises that will target the entire body, one joint at a time, from head to toes. Exercises will be performed in a seated position as much as possible. The stretching program will be based on the one developed by Stanziano et al.[7] The session will end with 5 minutes of relaxation. Variability in exercises performed between sessions will be encouraged in order to avoid progression throughout the entire program.

Details of Vitamin D Supplementation

Participants will receive Vitamin D supplementation (1 tablet of 10,000IU of Vitamin D3) or matching placebo three times per week in order to reach a weekly cumulative dose of 30,000 IU per week (equivalent of 4,258 IU daily). The vitamin D capsules will be provided by the Research Coordinator (RC) in vials at baseline assessment and every four weeks to complete 5 vials during the training period. Vials will be returned by the participants to the RC at the end of each 4 weeks block. Rationale and bio- safety of the dose: The dose of 10,000 IU/day is currently approved by Health Canada as a supplementation for elderly patients. Heaney et al. have administered doses of 30,000 IU/day Vitamin D3 to adult men for five months, with no significant changes in serum calcium concentrations or adverse reactions.[8-10] A comprehensive review of toxic effects of vitamin D found that the lowest level at which an adverse effect was observed was a serum calcidiol concentration of 200 nmol/l, corresponding to a daily intake of 40,000 IU. Therefore, three weekly doses of 10,000IU have a 9-fold weekly margin of safety of the established safe dose.

© 2023 Montero-Odasso M et al. *JAMA Network Open.*

Details of Placebo Vitamin D supplementation

Participants will receive Vitamin D supplementation (1 tablet of 10,000IU of Vitamin D3) or matching placebo three times per week in order to reach a weekly cumulative dose of 30,000 IU per week (equivalent of 4,258 IU daily). The vitamin D capsules will be provided by the Research Coordinator (RC) in vials at baseline assessment and every four weeks to complete 5 vials during the training period. Vials will be returned by the participants to the RC at the end of each 4 weeks block. Rationale and bio- safety of the dose: The dose of 10,000 IU/day is currently approved by Health Canada as a supplementation for elderly patients. Heaney et al. have administered doses of 30,000 IU/day Vitamin D3 to adult men for five months, with no significant changes in serum calcium concentrations or adverse reactions.[8-10] A comprehensive review of toxic effects of vitamin D found that the lowest level at which an adverse effect was observed was a serum calcidiol concentration of 200 nmol/l, corresponding to a daily intake of 40,000 IU. Therefore, three weekly doses of 10,000IU have a 9-fold weekly margin of safety of the established safe dose.

Supervision

All physical exercise training will be fully supervised by research assistants trained in exercise physiology or with kinesiology or physical therapy backgrounds or training. Training frequency is 3 days/week. Each session will take 90 minutes on average.

Additional tests included in the ADAS-Cog plus version

1. Trail-Making Test (TMT) A & B,
2. WAIS-R Digit Symbol Substitution Test (DSST)
3. Digit Span forward & backward (DSFB)
4. Category Fluency test (Animal and Vegetable)

© 2023 Montero-Odasso M et al. *JAMA Network Open.*

eTable 1. Details of the participants who were withdrawn from the SYNERGIC Trial due to medical reasons.

Arm	Withdrawal reason
1	None
2	Too sick to continue (Herpes zoster)
	Knee pain
	Back pain
	Hemorrhagic stroke due to amyloid angiopathy (bleeding in the basal ganglia affecting mobility)
	Hip pain
3	Too sick to continue (Pneumonia)
	Knee pain
	Systolic readings too high when exercising
	Hip fracture
4	Back pain
	Minor stroke
	New diagnosis of stage 2 breast cancer
	Too sick to continue (Shingles)
5	None

© 2023 Montero-Odasso M et al. *JAMA Network Open.*

eTable 2. The effect of exercise, with (arm 1+2) and without (arm 3+4) cognitive intervention on ADAS-Cog 13 scores at 6-month endpoint, compared to control (arm 5), and adjusted for age, sex, education, and comorbidities.

	Within-group difference from baseline			Between-group difference from baseline			
	Exercise and Cognitive Intervention (Arm 1+2)	Exercise (Arm 3+4)	Control (Arm 5)	Exercise and Cognitive Intervention (Arm 1+2) vs Control (Arm 5)		Exercise (Arm 3+4) vs. Control (Arm 5)	
	Mean (SE)	Mean (SE)	Mean (SE)	MD (95% CI)	p-value	MD (95% CI)	p-value
**Primary Outcomes**							
ADAS-Cog-13	-2.29 (0.45)	-0.75 (0.45)	0.38 (0.69)	-2.66 (-4.26, -1.06)	0.001	-1.13 (-2.74, 0.48)	0.17
ADAS-Cog-Plus	-0.13 (0.05)	0.01 (0.05)	-0.05 (0.08)	-0.09 (-0.26, 0.10)	0.38	0.06 (-0.12, 0.24)	0.51
**Individual items in ADAS-Cog**							
Word Recall	-0.28 (0.14)	-0.38 (0.15)	0.03 (0.22)	-0.31 (-0.83, 0.20)	0.23	-0.42 (-0.93, 0.10)	0.12
Delayed Recall	-0.44 (0.22)	-0.18 (0.23)	0.29 (0.34)	-0.73 (-1.52, 0.07)	0.08	-0.46 (-1.26, 0.34)	0.26
Following Commands	0.008 (0.08)	0.13 (0.08)	0.19 (0.13)	-0.18 (-0.48, 0.12)	0.23	-0.06 (-0.36, 0.24)	0.70
Constructional Praxis	0.09 (0.12)	-0.03 (0.12)	-0.27 (0.18)	0.36 (-0.05, 0.77)	0.09	0.24 (-0.17, 0.65)	0.25
Ideational Praxis	0.02 (0.05)	0.02 (0.05)	0.03 (0.08)	-0.006 (-0.19, 0.17)	0.95	-0.006 (-0.19, 0.17)	0.95
Naming Objects	-0.64 (0.18)	-0.06 (0.18)	-0.17 (0.27)	-0.47 (-1.10, 0.15)	0.14	0.11 (-0.52, 0.73)	0.74
Orientation	-0.19 (0.12)	-0.03 (0.12)	0.43 (0.18)	-0.63 (-1.05, -0.20)	0.004	-0.46 (-0.88, -0.03)	0.04
Word Recognition	-0.69 (0.24)	-0.04 (0.24)	0.41 (0.38)	-1.09 (-1.97, -0.22)	0.02	-0.45 (-1.32, 0.43)	0.32
Remembering Instructions	-0.006 (0.05)	-0.06 (0.05)	-0.05 (0.08)	0.05 (-0.14, 0.23)	0.61	-0.004 (-0.19, 0.18)	0.97
Comprehension	-0.01 (0.06)	-0.03 (0.06)	0.02 (0.09)	-0.03 (-0.25, 0.18)	0.76	-0.05 (-0.27, 0.16)	0.63
Word Finding	0.02 (0.07)	-0.05 (0.07)	-0.14 (0.11)	0.16 (-0.10, 0.41)	0.23	0.09 (-0.17, 0.34)	0.50
Spoken Language	-0.03 (0.06)	-0.13 (0.06)	-0.13 (0.09)	0.01 (-0.10, 0.31)	0.33	-0.001 (-0.21, 0.21)	0.99
Number Cancellation	-0.15 (0.11)	0.13 (0.11)	-0.09 (0.17)	-0.06 (-0.45, 0.32)	0.77	0.22 (-0.17, 0.61)	0.26
Trail Making A	-3.30 (1.92)	-1.93 (1.95)	5.43 (2.98)	-8.74 (-15.68, -1.86)	0.01	-7.36 (-14.35, -0.46)	0.04
Trail Making B	7.92 (6.83)	-7.26 (6.89)	4.21 (10.69)	3.71 (-21.00, 28.49)	0.77	-11.48 (-36.29, 13.31)	0.37
Digit Symbol Substitution	1.41 (1.10)	0.61 (1.17)	-1.84 (1.74)	3.24 (-0.78, 7.26)	0.12	2.44 (-1.65, 6.54)	0.25
WAIS III Digit Span Forward	-1.14 (0.40)	-1.20 (0.40)	-0.71 (0.61)	-0.43 (-1.85, 0.98)	0.55	-0.49 (-1.91, 0.92)	0.50
WAIS III Digit Span Backward	-1.20 (0.31)	-0.78 (0.31)	-0.55 (0.47)	-0.65 (-1.75, 0.45)	0.25	-0.23 (-1.32, 0.88)	0.69
Category Fluency Animals	0.82 (0.50)	-0.41 (0.51)	1.50 (0.77)	-0.68 (-2.45, 1.11)	0.46	-1.91 (-3.70, -0.12)	0.04
Category Fluency Vegetables	0.09 (0.44)	-0.78 (0.44)	0.87 (0.67)	-0.78 (-2.33, 0.79)	0.33	-1.65 (-3.21, -0.09)	0.04

CI, Confidence interval; MD, Mean Difference; SE, Standard error.
Adjusted for age, sex, number of years of education, and number of comorbidities. Marginal means and standard errors obtained from linear mixed models are reported for within-group differences. Between-group differences were assessed using the interaction between time x intervention arm. Lower scores indicate cognitive improvement.

© 2023 Montero-Odasso M et al. *JAMA Network Open.*

eTable 3. Within-group and between-group intervention effect across 5 intervention arms at 6-month endpoint.

	Within group difference from baseline					Between group difference											
	Arm 1	Arm 2	Arm 3	Arm 4	Arm 5	Arm 1 vs Arm 5			Arm 2 vs. Arm 5			Arm 3 vs. Arm 5			Arm 4 vs. Arm 5		
	mean (SE)	mean (SE)	mean (SE)	mean (SE)	mean (SE)	MD (95% CI)	p	ES	MD (95% CI)	p	ES	MD (95% CI)	p	ES	MD (95% CI)	p	ES
**Primary Outcome**																	
ADAS-Cog-13	-2.41 (0.62)	-2.16 (0.65)	-0.38 (0.62)	-1.34 (0.65)	0.23 (0.67)	-2.64 (-4.42,-0.87)	0.005	0.71	-2.39 (-4.20, -0.57)	0.01	0.63	-0.61 (-2.39,1.16)	0.50	0.16	-1.57 (-3.38,0.25)	0.10	0.41
ADAS-Cog-Plus	-0.16 (0.07)	-0.09 (0.07)	0.02 (0.07)	0.004 (0.07)	-0.07 (0.08)	-0.09 (-0.29, 0.11)	0.39	0.21	-0.02 (-0.22, 0.19)	0.88	0.04	0.09 (-0.11, 0.29)	0.37	-0.22	0.08 (-0.12, 0.28)	0.46	-0.18
**Individual items in ADAS-Cog**																	
Word Recall	-0.41 (0.20)	-0.16 (0.21)	-0.37 (0.20)	-0.41 (0.21)	0.02 (0.22)	-0.43 (-0.99, 0.14)	0.15	0.35	-0.18 (-0.76, 0.40)	0.55	0.15	-0.18 (-0.76, 0.40)	0.19	0.32	-0.43 (-1.01, 0.16)	0.16	0.35
Delayed Recall	-0.21 (0.31)	-0.72 (0.32)	-0.46 (0.31)	0.01 (0.32)	0.27 (0.33)	-0.48 (-1.36, 0.39)	0.29	0.26	-0.99 (-1.88, -0.09)	0.03	0.53	-0.73 (-1.61, 0.14)	0.11	0.39	-0.26 (-1.15, 0.64)	0.58	0.14
Following Commands	0.08 (0.12)	0.10 (0.12)	0.13 (0.12)	0.07 (0.12)	0.19 (0.12)	-0.27 (-0.60, 0.06)	0.11	0.39	-0.09 (-0.43, 0.24)	0.58	0.14	-0.06 (-0.39, 0.27)	0.71	0.09	-0.13 (-0.46, 0.21)	0.46	0.18
Constructional Praxis	0.005 (0.16)	0.15 (0.16)	0.14 (0.16)	-0.22 (0.16)	-0.27 (0.17)	0.28 (-0.18, 0.73)	0.23	-0.29	0.42 (-0.04, 0.87)	0.07	-0.44	0.41 (-0.04, 0.85)	0.08	-0.43	0.06 (-0.40, 0.51)	0.81	-0.06
Ideational Praxis	0.006 (0.07)	0.05 (0.07)	-0.007 (0.07)	0.04 (0.07)	0.03 (0.08)	0.03 (-0.23, 0.17)	0.80	0.06	0.01 (-0.19, 0.22)	0.90	-0.03	-0.04 (-0.24, 0.16)	0.70	-0.09	-0.01 (-0.19, 0.22)	0.91	0.03
Naming Objects	-0.65 (0.24)	-0.60 (0.25)	-0.07 (0.24)	-0.07 (0.25)	-0.10 (0.26)	-0.55 (-1.24, 0.14)	0.12	0.38	-0.50 (-1.21, 0.20)	0.17	0.34	0.03 (-0.66, 0.72)	0.94	-0.02	0.03 (-0.67, 0.74)	0.93	-0.02
Orientation	-0.18 (0.17)	-0.22 (0.17)	0.06 (0.17)	-0.16 (0.17)	0.39 (0.18)	-0.57 (-1.05, -0.10)	0.02	0.57	-0.61 (-1.09, -0.13)	0.02	0.60	-0.57 (-1.05, -0.10)	0.17	0.33	-0.55 (-1.04, -0.07)	0.03	0.55
Word Recognition	-1.00 (0.34)	-0.39 (0.35)	0.07 (0.34)	-0.19 (0.35)	0.33 (0.37)	-1.33 (-2.32, -0.36)	0.009	0.64	-0.72 (-1.71, 0.27)	0.16	0.35	-0.26 (-1.23, 0.71)	0.61	0.13	-0.53 (-1.52, 0.47)	0.31	0.25
Remembering Instructions	0.004 (0.07)	-0.02 (0.82)	-0.08 (0.26)	-0.03 (0.71)	-0.05 (0.08)	0.05 (-0.15, 0.25)	0.63	-0.12	0.03 (-0.18, 0.23)	0.78	-0.07	-0.03 (-0.23, 0.17)	0.74	0.08	0.02 (-0.19, 0.22)	0.86	-0.04
Comprehension	-0.02 (0.08)	-0.01 (0.09)	0.02 (0.08)	-0.08 (0.09)	0.03 (0.09)	-0.05 (-0.29, 0.19)	0.68	0.10	-0.03 (-0.28, 0.21)	0.78	0.07	-0.01 (-0.25, 0.22)	0.92	0.03	-0.11 (-0.35, 0.13)	0.37	0.22
Word Finding	0.03 (0.10)	0.01 (0.11)	-0.04 (0.10)	-0.11 (0.11)	-0.09 (0.11)	0.12 (-0.18, 0.41)	0.43	-0.19	0.10 (-0.20, 0.40)	0.52	-0.16	0.05 (-0.24, 0.34)	0.75	-0.08	-0.02 (-0.32, 0.28)	0.90	0.03
Spoken Language	-0.03 (0.08)	-0.02 (0.08)	-0.11 (0.08)	-0.14 (0.08)	-0.12 (0.09)	0.09 (-0.14, 0.32)	0.43	-0.19	0.10 (-0.13, 0.33)	0.39	-0.21	0.01 (-0.22, 0.24)	0.93	-0.02	-0.02 (-0.26, 0.21)	0.85	0.05
Number Cancellation	-0.08 (0.15)	-0.20 (0.15)	0.26 (0.14)	-0.009 (0.15)	-0.14 (0.16)	0.05 (-0.37, 0.47)	0.81	-0.06	-0.06 (-0.49, 0.37)	0.79	0.07	0.40 (-0.02, 0.81)	0.07	-0.44	0.13 (-0.30, 0.56)	0.55	-0.15

© 2023 Montero-Odasso M et al. *JAMA Network Open.*

Trail Making A	-6.0 (2.68)	-0.21 (2.78)	-1.87 (2.66)	-0.46 (2.85)	5.25 (2.98)	-11.25 (-19.02, -3.49)	0.006	0.69	-5.46 (-13.38, 2.43)	0.18	0.34	-7.12 (-14.88, 0.62)	0.08	0.43	-5.71 (-13.72, 2.27)	0.17	0.34
Trail Making B	14.30 (9.44)	0.19 (9.87)	-4.89 (9.24)	-7.11 (10.11)	5.18 (10.64)	9.13 (-18.42, 36.65)	0.52	-0.16	-4.99 (-33.08, 23.10)	0.73	0.09	-10.06 (-37.37, 17.20)	0.48	0.17	-12.29 (-40.69, 16.12)	0.40	0.21
Digit Symbol Substitution	1.60 (1.53)	1.16 (1.60)	-0.06 (1.57)	0.98 (1.73)	-1.29 (1.72)	2.89 (-1.56, 7.35)	0.21	-0.31	2.45 (-2.11, 7.00)	0.30	-0.26	1.34 (-3.28, 5.74)	0.60	-0.13	2.27 (-2.46, 6.98)	0.35	-0.23
WAIS III Digit Span Forward	-1.26 (0.56)	-1.14 (0.58)	-1.41 (0.56)	-0.99 (0.58)	-0.83 (0.60)	-0.43 (-2.02, 1.16)	0.60	0.13	-0.31 (-1.93, 1.31)	0.71	0.09	-0.58 (-2.16, 1.01)	0.48	0.17	-0.16 (-1.78, 1.46)	0.85	0.05
WAIS III Digit Span Backward	-1.27 (0.43)	-1.19 (0.45)	-1.12 (0.43)	-0.50 (0.45)	-0.66 (0.47)	-0.60 (-1.83, 0.63)	0.34	0.23	-0.53 (-1.78, 0.73)	0.42	0.20	-0.46 (-1.68, 0.77)	0.47	0.17	0.17 (-1.09, 1.42)	0.80	-0.06
Category Fluency Animals	0.37 (0.69)	1.27 (0.72)	0.32 (0.70)	-1.12 (0.72)	1.63 (0.74)	-1.26 (-3.22, 0.71)	0.22	0.30	-0.36 (-2.36, 1.64)	0.73	0.09	-1.31 (-3.28, 0.67)	0.20	0.31	-2.75 (-4.76, -0.74)	0.009	0.65
Category Fluency Vegetables	0.09 (0.62)	0.07 (0.64)	-0.96 (0.61)	-0.37 (0.64)	1.11 (0.66)	-1.02 (-2.77, 0.74)	0.26	0.27	-1.04 (-2.82, 0.74)	0.26	0.28	-2.07 (-3.81, -0.34)	0.02	0.56	-1.48 (-3.26, 0.30)	0.11	0.40

CI, Confidence interval; ES, Effect size; MD, Mean Difference; p, p-value; SE, Standard error.

Marginal means and standard errors obtained from linear mixed models are reported for within-group differences. Between-group differences were assessed using the interaction between time x intervention arm. Lower scores indicate cognitive improvement.

© 2023 Montero-Odasso M et al. *JAMA Network Open.*

eTable 4. Within-group and between-group intervention effect across 5 intervention arms at 6-month endpoint, adjusted for age, sex, education, and comorbidities.

	Within-group difference from baseline					Between-group difference from baseline							
	Arm 1 mean (SE)	Arm 2 mean (SE)	Arm 3 mean (SE)	Arm 4 mean (SE)	Arm 5 mean (SE)	Arm 1 vs Arm 5 MD (95% CI)	p	Arm 2 vs. Arm 5 MD (95% CI)	p	Arm 3 vs. Arm 5 MD (95% CI)	p	Arm 4 vs. Arm 5 MD (95% CI)	p
**Primary Outcomes**													
ADAS-Cog-13	-2.42 (0.62)	-2.14 (0.65)	-0.32 (0.62)	-1.25 (0.66)	0.38 (0.69)	-2.80 (-4.61, -0.98)	0.003	-2.51 (-4.37, -0.66)	0.009	-0.70 (-2.51, 1.11)	0.45	-1.62 (-3.49, 0.25)	0.07
ADAS-Cog-Plus	-0.16 (0.07)	-0.09 (0.07)	0.02 (0.07)	0.01 (0.07)	-0.04 (0.08)	-0.12 (-0.32, 0.08)	0.25	-0.05 (-0.25, 0.16)	0.65	0.07 (-0.13, 0.27)	0.52	0.06 (-0.15, 0.26)	0.59
**Individual items in ADAS-Cog**													
Word Recall	-0.34 (0.20)	-0.18 (0.21)	-0.36 (0.20)	-0.44 (0.21)	-0.01 (0.22)	-0.33 (-0.90, 0.25)	0.27	-0.17 (-0.75, 0.42)	0.58	-0.35 (-0.92, 0.23)	0.24	-0.43 (-1.02, 0.17)	0.16
Delayed Recall	-0.12 (0.31)	-0.72 (0.32)	-0.40 (0.31)	0.01 (0.33)	0.27 (0.34)	-0.39 (-1.28, 0.50)	0.40	-0.99 (-1.90, -0.08)	0.03	-0.67 (-1.56, 0.22)	0.14	-0.26 (-1.17, 0.66)	0.59
Following Commands	-0.08 (0.12)	0.10 (0.12)	0.16 (0.12)	0.10 (0.12)	0.19 (0.13)	-0.28 (-0.61, 0.07)	0.12	-0.09 (-0.44, 0.25)	0.61	-0.03 (-0.37, 0.31)	0.85	-0.09(-0.44, 0.26)	0.62
Constructional Praxis	0.02 (0.16)	0.16 (0.17)	0.14 (0.16)	-0.21 (0.17)	-0.27 (0.17)	0.29 (-0.17, 0.76)	0.22	0.43 (-0.04, 0.90)	0.08	0.41 (-0.05, 0.87)	0.09	0.06 (-0.41, 0.54)	0.79
Ideational Praxis	-0.66 (0.24)	-0.62 (0.25)	-0.08 (0.24)	-0.04 (0.26)	-0.17 (0.27)	-0.50 (-1.21, 0.21)	0.17	-0.45 (-1.18, 0.27)	0.22	0.09 (-0.62, 0.80)	0.81	0.13 (-0.60, 0.86)	0.73
Naming Objects	-0.16 (0.17)	-0.23 (0.17)	0.11 (0.16)	-0.19 (0.18)	0.43 (0.18)	-0.60 (-1.08, -0.12)	0.02	-0.66 (-1.15, -0.17)	0.01	-0.32 (-0.80, 0.16)	0.19	-0.32 (-0.80, 0.16)	0.19
Orientation	-0.99 (0.34)	-0.38 (0.35)	0.14 (0.33)	-0.25 (0.36)	0.41 (0.38)	-1.40 (-2.39, -0.40)	0.007	-0.78 (-1.79, 0.22)	0.13	-0.27 (-1.26, 0.72)	0.59	-0.66 (-1.68, 0.36)	0.21
Word Recognition	0.004 (0.07)	-0.02 (0.07)	-0.08 (0.07)	-0.03 (0.08)	-0.05 (0.08)	0.06 (-0.15, 0.27)	0.59	0.04 (-0.18, 0.25)	0.74	-0.03 (-0.24, 0.18)	0.80	0.02 (-0.19, 0.24)	0.84
Remembering Instructions	-0.03 (0.09)	-0.004 (0.09)	0.02 (0.09)	-0.09 (0.09)	0.02 (0.09)	-0.04 (-0.29, 0.21)	0.74	-0.02 (-0.28, 0.23)	0.85	-0.004 (-0.25, 0.24)	0.98	-0.11 (-0.37, 0.14)	0.39
Comprehension	0.02 (0.10)	0.007 (0.10)	-0.05 (0.10)	-0.05 (0.11)	-0.14 (0.11)	0.16 (-0.13, 0.45)	0.28	0.15 (-0.15, 0.44)	0.34	0.09 (-0.20, 0.38)	0.54	0.08 (-0.22, 0.38)	0.58
Word Finding	-0.03 (0.08)	-0.02 (0.08)	-0.11 (0.08)	-0.15 (0.09)	-0.13 (0.09)	0.10 (-0.14, 0.34)	0.40	0.11 (-0.13, 0.35)	0.37	0.02 (-0.22, 0.26)	0.88	-0.02 (-0.27, 0.22)	0.87
Spoken Language	-0.10 (0.15)	-0.21 (0.15)	0.25 (0.15)	-0.003 (0.16)	-0.09 (0.17)	-0.004 (-0.44, 0.43)	0.99	-0.12 (-0.56, 0.32)	0.603	0.34 (-0.09, 0.77)	0.13	0.09 (-0.36, 0.53)	0.70
Number Cancellation	-6.19 (2.66)	-0.19 (2.76)	-2.05 (2.64)	-1.79 (2.88)	5.43 (2.98)	-11.62 (-19.44, -3.80)	0.004	-5.61 (-13.57, 2.34)	0.17	-7.47 (-15.27, 0.33)	0.06	-7.22 (-15.33, 0.90)	0.08
Trail Making A	14.93 (9.49)	0.23 (9.91)	-5.84 (9.29)	-8.97 (10.36)	4.20 (10.72)	10.73 (-17.32, 38.78)	0.45	-3.97 (-32.57, 24.63)	0.79	-10.04 (-37.82, 17.74)	0.48	-13.17 (-42.37, 16.03)	0.38
Trail Making B	1.61 (1.53)	1.17 (1.60)	0.16 (1.58)	1.16 (1.77)	-1.84 (1.76)	3.45 (-1.12, 8.01)	0.14	3.01 (-1.65, 7.67)	0.21	2.00 (-2.62, 6.62)	0.40	3.00 (-1.89, 7.88)	0.23

© 2023 Montero-Odasso M et al. *JAMA Network Open.*

	Within-group difference from baseline					Between-group difference from baseline							
	Arm 1 mean (SE)	Arm 2 mean (SE)	Arm 3 mean (SE)	Arm 4 mean (SE)	Arm 5 mean (SE)	Arm 1 vs Arm 5		Arm 2 vs. Arm 5		Arm 3 vs. Arm 5		Arm 4 vs. Arm 5	
						MD (95% CI)	p	MD (95% CI)	p	MD (95% CI)	p	MD (95% CI)	p
Digit Symbol Substitution	-1.18 (0.55)	-1.10 (0.57)	-1.47 (0.55)	-0.89 (0.59)	-0.71 (0.61)	-0.48 (-2.09, 1.14)	0.56	-0.40 (-2.04, 1.24)	0.63	-0.77 (-2.38, 0.84)	0.35	-0.19 (-1.84, 1.47)	0.83
WAIS III Digit Span Forward	-1.23 (0.43)	-1.18 (0.44)	-1.11 (0.43)	-0.40 (0.46)	-0.55 (0.47)	-0.68 (-1.93, 0.57)	0.29	-0.63 (-1.90, 0.64)	0.33	-0.56 (-1.81, 0.68)	0.38	0.15 (-1.13, 1.44)	0.81
WAIS III Digit Span Backward	0.36 (0.69)	1.33 (0.72)	0.28 (0.70)	-1.18 (0.74)	1.50 (0.76)	-1.14 (-3.16, 0.87)	0.27	-0.17 (-2.23, 1.88)	0.87	-1.22 (-3.25, 0.80)	0.24	-2.69 (-4.76, -0.61)	0.01
Category Fluency Animals	0.36 (0.69)	1.33 (0.72)	0.28 (0.70)	-1.18 (0.74)	1.50 (0.76)	-1.14 (-3.16, 0.87)	0.27	-0.17 (-2.23, 1.88)	0.87	-1.22 (-3.25, 0.80)	0.24	-2.69 (-4.76, -0.61)	0.01
Category Fluency Vegetables	0.09 (0.62)	0.09 (0.64)	-1.02 (0.61)	-0.51 (0.65)	0.87 (0.67)	-0.78 (-2.57, 1.01)	0.39	-0.78 (-2.59, 1.03)	0.40	-1.89 (-3.66, -0.12)	0.04	-1.38 (-3.21, 0.45)	0.14

CI, Confidence interval; MD, Mean Difference; SE, Standard error.
Adjusted for age, sex, number of years of education, and number of comorbidities. Marginal means and standard errors obtained from linear mixed models are reported for within-group differences. Between-group differences were assessed using the interaction between time x intervention arm. Lower scores indicate cognitive improvement.

© 2023 Montero-Odasso M et al. *JAMA Network Open.*

eTable 5. Number of participants with improvement in ADAS-Cog-13 and plus variant at 6 months by intervention arm

	Overall (N=171)	Arm 1 (N=34)	Arm 2 (N=35)	Arm 3 (N=36)	Arm 4 (N=33)	Arm 5 (N=33)	p-value*
ADAS-Cog-13							
Any improvement	91 (53.2%)	22 (64.7%)	22 (62.9%)	18 (50.0%)	18 (54.5%)	11 (33.3%)	0.06
Clinical improvement	46 (26.9%)	15 (44.1%)	13 (37.1%)	5 (13.9%)	8 (24.2%)	5 (15.2%)	0.02
ADAS-Cog-Plus							
Any improvement	87 (50.9%)	20 (58.8%)	19 (54.3%)	14 (38.9%)	16 (48.5%)	18 (54.5%)	0.30

Note: Any improvement was defined as any decline in ADAS-Cog from baseline to month 6 (lower score indicates cognitive improvement)
Clinical improvement was defined as a decline of 3 or more points in ADAS-Cog-13 from baseline to month 6 (lower score indicates cognitive improvement)
* Chi-square test was used to compare the proportion of participants with improvement across the arms.

© 2023 Montero-Odasso M et al. *JAMA Network Open.*

eTable 6. Per-protocol analysis for the effect of within-group and between-group intervention across 5 intervention arms at 6-month endpoint.

	Within-group difference from baseline					Between-group difference from baseline							
	Arm 1	Arm 2	Arm 3	Arm 4	Arm 5	Arm 1 vs Arm 5		Arm 2 vs. Arm 5		Arm 3 vs. Arm 5		Arm 4 vs. Arm 5	
	mean (SE)	mean (SE)	mean (SE)	mean (SE)	mean (SE)	MD (95% CI)	p	MD (95% CI)	p	MD (95% CI)	p	MD (95% CI)	p
**Primary Outcomes**													
ADAS-Cog-13	-2.30 (0.62)	-2.30 (0.65)	-0.33 (0.62)	-1.32 (0.65)	0.17 (0.68)	-2.47 (-4.25, -0.69)	0.008	-2.46 (-4.29, -0.64)	0.01	-0.50 (-2.28, 1.29)	0.59	-1.49 (-3.32, 0.34)	0.12
ADAS-Cog-Plus	-0.16 (0.07)	-0.10 (0.07)	0.02 (0.07)	-0.002 (0.07)	-0.08 (0.08)	-0.08 (-0.28, 0.12)	0.46	-0.02 (-0.22, 0.19)	0.87	0.10 (-0.10, 0.30)	0.33	0.08 (-0.13, 0.28)	0.47
**Individual items in ADAS-Cog**													
Word Recall	-0.40 (0.20)	-0.23 (0.21)	-0.33 (0.20)	-0.39 (0.21)	0.05 (0.22)	-0.45 (-1.03, 0.13)	0.13	-0.28 (-0.87, 0.31)	0.36	-0.38 (-0.96, 0.19)	0.20	-0.45 (-1.04, 0.15)	0.15
Delayed Recall	-0.16 (0.31)	-0.82 (0.32)	-0.45 (0.31)	0.11 (0.32)	0.27 (0.34)	-0.43 (-1.31, 0.45)	0.35	-1.09 (-1.99, -0.19)	0.02	-0.72 (-1.60, 0.16)	0.12	-0.16 (-1.07, 0.74)	0.73
Following Commands	-0.06 (0.12)	0.14 (0.12)	0.16 (0.12)	0.11 (0.12)	0.23 (0.13)	-0.30 (-0.63, 0.04)	0.09	-0.09 (-0.43, 0.26)	0.62	-0.07 (-0.41, 0.27)	0.68	-0.12 (-0.47, 0.22)	0.48
Constructional Praxis	0.13 (0.16)	0.18 (0.16)	0.16 (0.16)	0.25 (0.16)	-0.35 (0.17)	0.48 (0.03, 0.92)	0.04	0.52 (0.07, 0.98)	0.03	0.51 (0.06, 0.95)	0.03	0.10 (-0.36, 0.55)	0.68
Ideational Praxis	0.00 (0.07)	0.04 (0.08)	0.00 (0.07)	0.07 (0.08)	0.04 (0.08)	-0.04 (-0.25, 0.17)	0.71	-0.004 (-0.22, 0.21)	0.97	-0.04 (-0.25, 0.17)	0.71	0.03 (-0.18, 0.24)	0.77
Naming Objects	-0.61 (0.25)	-0.64 (0.26)	-0.06 (0.25)	-0.11 (0.26)	-0.15 (0.27)	-0.46 (-1.16, 0.24)	0.21	-0.49 (-1.21, 0.23)	0.19	0.09 (-0.61, 0.79)	0.81	0.05 (-0.67, 0.77)	0.90
Orientation	-0.13 (0.17)	-0.25 (0.18)	0.10 (0.17)	-0.21 (0.18)	0.42 (0.18)	-0.55 (-1.04, -0.07)	0.03	-0.67 (-1.17, -0.18)	0.009	-0.33 (-0.81, 0.16)	0.19	-0.64 (-1.13, -0.14)	0.01
Word Recognition	-0.90 (0.35)	-0.46 (0.36)	0.10 (0.34)	-0.14 (0.36)	0.28 (0.38)	-1.18 (-2.18, -0.18)	0.02	-0.74 (-1.76, 0.27)	0.16	-0.18 (-1.18, 0.81)	0.72	-0.42 (-1.44, 0.59)	0.42
Remembering Instructions	0.00 (0.07)	0.00 (0.08)	-0.10 (0.07)	-0.04 (0.08)	-0.08 (0.08)	0.08 (-0.14, 0.29)	0.48	0.08 (-0.14, 0.29)	0.49	-0.02 (-0.23, 0.19)	0.86	0.04 (-0.18, 0.26)	0.71
Comprehension	-0.03 (0.09)	0.00 (0.09)	0.00 (0.09)	-0.11 (0.09)	0.00 (0.10)	-0.03 (-0.29, 0.22)	0.81	0.00 (-0.26, 0.26)	0.99	0.00 (-0.25, 0.25)	0.99	-0.11 (-0.37, 0.15)	0.43
Word Finding	0.03 (0.11)	0.00 (0.11)	-0.06 (0.11)	-0.14 (0.11)	-0.12 (0.12)	0.15 (-0.16, 0.45)	0.35	0.12 (-0.20, 0.43)	0.48	0.05 (-0.25, 0.36)	0.75	-0.03 (-0.34, 0.29)	0.87
Spoken Language	-0.03 (0.08)	-0.04 (0.09)	-0.13 (0.08)	-0.18 (0.09)	-0.15 (0.09)	0.12 (-0.12, 0.36)	0.33	0.12 (-0.13, 0.37)	0.36	0.02 (-0.22, 0.27)	0.84	-0.02 (-0.27, 0.22)	0.85
Number Cancellation	-0.10 (0.15)	-0.21 (0.15)	0.29 (0.15)	-0.04 (0.15)	-0.17 (0.17)	0.07 (-0.36, 0.50)	0.75	-0.05 (-0.48, 0.39)	0.83	0.46 (0.03, 0.88)	0.04	0.13 (-0.31, 0.57)	0.56
Trail Making A	-6.10 (2.72)	-0.26 (2.87)	-1.65 (2.72)	-0.44 (2.92)	4.51 (3.10)	-10.61 (-18.60, -2.61)	0.01	-4.76 (-12.94, 3.41)	0.26	-6.16 (-14.15, 1.83)	0.14	-4.95 (-13.20, 3.29)	0.25
Trail Making B	15.94 (9.40)	1.51 (9.93)	-2.76 (9.25)	-6.29 (10.13)	6.17 (10.8)	9.77 (-18.0, 37.5)	0.50	-4.66 (-33.1, 23.8)	0.75	-8.93 (-36.5, 18.6)	0.53	-12.46 (-41.1, 16.2)	0.40

© 2023 Montero-Odasso M et al. *JAMA Network Open.*

	Within-group difference from baseline					Between-group difference from baseline							
	Arm 1	Arm 2	Arm 3	Arm 4	Arm 5	Arm 1 vs Arm 5		Arm 2 vs. Arm 5		Arm 3 vs. Arm 5		Arm 4 vs. Arm 5	
	mean (SE)	mean (SE)	mean (SE)	mean (SE)	mean (SE)	MD (95% CI)	p	MD (95% CI)	p	MD (95% CI)	p	MD (95% CI)	p
Digit Symbol Substitution	1.62 (1.55)	1.39 (1.63)	0.03 (1.60)	1.21 (1.76)	-1.33 (1.76)	2.95 (-1.58, 7.49)	0.21	2.73 (-1.91, 7.36)	0.27	1.37 (-3.23, 5.97)	0.57	2.54 (-2.27, 7.35)	0.31
WAIS III Digit Span Forward	-1.29 (0.56)	-1.04 (0.59)	-1.65 (0.56)	-0.89 (0.59)	-0.77 (0.61)	-0.52 (-2.12, 1.08)	0.53	-0.27 (-1.90, 1.37)	0.75	-0.88 (-2.47, 0.72)	0.29	-0.12 (-1.76, 1.51)	0.88
WAIS III Digit Span Backward	-1.29 (0.43)	-1.21 (0.45)	-1.29 (0.43)	-0.57 (0.45)	-0.62 (0.47)	-0.67 (-1.91, 0.56)	0.29	-0.60 (-1.87, 0.67)	0.36	-0.67 (-1.91, 0.56)	0.29	0.04 (-1.22, 1.31)	0.95
Category Fluency Animals	0.32 (0.70)	1.29 (0.73)	0.30 (0.71)	-1.11 (0.73)	1.81 (0.76)	-1.49 (-3.48, 0.51)	0.15	-0.52 (-2.56, 1.52)	0.62	-1.51 (-3.52, 0.50)	0.15	-2.91 (-4.96, -0.87)	0.006
Category Fluency Vegetables	0.13 (0.63)	0.07 (0.65)	-0.90 (0.62)	-0.29 (0.65)	1.15 (0.67)	-1.02 (-2.80, 0.76)	0.09	-1.08 (-2.89,0.73)	0.25	-2.06 (-3.82, -0.29)	0.03	-1.44 (-3.25, 0.37)	0.13

CI, Confidence interval; MD, Mean Difference; SE, Standard error.
Marginal means and standard errors obtained from linear mixed models are reported for within-group differences. Between-group differences were assessed using the interaction between time x intervention arm. Lower scores indicate cognitive improvement.

© 2023 Montero-Odasso M et al. *JAMA Network Open.*

eTable 7. Per-protocol analysis for the effect of exercise intervention with addition of cognitive training and vitamin D at 6-month endpoint.

	Mean Change (SE) within group	Mean Difference between group (95% CI)	p-value
**ADAS-Cog-13**			
**Exercise intervention**			
Arm 1+4 (Exercise)	-1.55 (0.32)	-1.72 (-3.21, -0.23)	0.03
Arm 5 (Control)	0.17 (0.69)		
**Adding Cognitive intervention**			
Arm 1+2 (Exercise + Cognitive training)	-2.30 (0.45)	-1.50 (-2.75, -0.25)	0.02
Arm 3+4 (Exercise)	-0.80 (0.45)		
**Adding Vitamin D intervention**			
Arm 1+3 (Exercise + Vitamin D)	-1.32 (0.45)	0.49 (-0.78, 1.77)	0.45
Arm 2+4 (Exercise)	-1.81 (0.47)		
**ADAS-Cog-Plus**			
**Exercise intervention**			
Arm 1+4 (Exercise)	-0.06 (0.04)	0.02 (-0.14, 0.19)	0.81
Arm 5 (Control)	-0.08 (0.08)		
**Adding Cognitive intervention**			
Arm 1+2 (Exercise + Cognitive training)	-0.13 (0.05)	-0.14 (-0.27, -0.001)	0.05
Arm 3+4 (Exercise)	0.01 (0.05)		
**Adding Vitamin D intervention**			
Arm 1+3 (Exercise + Vitamin D)	-0.07 (0.05)	-0.02 (-0.16, 0.12)	0.80
Arm 2+4 (Exercise)	-0.05 (0.05)		

CI, Confidence interval; MD, Mean Difference; SE, Standard error.
Marginal means and standard errors obtained from linear mixed models are reported for within-group differences. Between-group differences were assessed using the interaction between time x intervention arm. Lower scores indicate cognitive improvement.

© 2023 Montero-Odasso M et al. *JAMA Network Open.*

eTable 8. Per-protocol analysis for the effect of exercise and cognitive intervention and exercise alone at 6-month endpoint.

	Within-group difference from baseline			Between-group difference from baseline			
	Exercise and Cognitive Intervention (Arm 1+2)	Exercise (Arm 3+4)	Control (Arm 5)	Exercise and Cognitive Intervention (Arm 1+2) vs Control (Arm 5)		Exercise (Arm 3+4) vs. Control (Arm 5)	
	Mean (SE)	Mean (SE)	Mean (SE)	MD (95% CI)	p-value	MD (95% CI)	p-value
**Primary Outcomes**							
ADAS-Cog-13	-2.30 (0.45)	-0.80 (0.45)	0.17 (0.68)	-2.47 (-4.05, -0.88)	0.003	-0. 97(-2.55, 0.62)	0.24
ADAS-Cog-Plus	-0.13 (0.05)	0.01 (0.05)	-0.08 (0.08)	-0.05 (-0.22, 0.13)	0.60	0.09 (-0.09, 0.27)	0.33
**Individual items in ADAS-Cog**							
Word Recall	-0.32 (0.15)	-0.36 (0.15)	0.05 (0.22)	-0.37 (-0.88, 0.14)	0.16	-0.41 (-0.92, 0.10)	0.12
Delayed Recall	-0.48 (0.22)	-0.19 (0.22)	0.27 (0.34)	-0.74 (-1.54, 0.05)	0.07	-0.46 (-1.25, 0.34)	0.26
Following Commands	0.03 (0.08)	0.14 (0.08)	0.23 (0.13)	-0.20 (-0.49, 0.10)	0.20	-0.10 (-0.39, 0.20)	0.53
Constructional Praxis	0.15 (0.11)	-0.03 (0.11)	-0.35 (0.17)	0.50 (0.10, 0.90)	0.02	0.31 (-0.09, 0.71)	0.13
Ideational Praxis	0.02 (0.05)	0.03 (0.05)	0.04 (0.08)	-0.02 (-0.21, 0.16)	0.81	-0.006 (-0.19, 0.18)	0.95
Naming Objects	-0.63 (0.18)	-0.08 (0.18)	-0.15 (0.27)	-0.47 (-1.10, 0.15)	0.14	0.07 (-0.55, 0.69)	0.83
Orientation	-0.19 (0.12)	-0.05 (0.12)	0.42 (0.18)	-0.61 (-1.04, -0.18)	0.007	-0.47 (-0.91, -0.04)	0.03
Word Recognition	-0.69 (0.25)	-0.02 (0.25)	0.28 (0.38)	-0.97 (-1.86, -0.08)	0.03	-0.30 (-1.19, 0.59)	0.51
Remembering Instructions	0.00 (0.05)	-0.07 (0.05)	-0.08 (0.08)	0.08 (-0.11, 0.27)	0.43	0.009 (-0.18, 0.20)	0.92
Comprehension	-0.02 (0.06)	-0.05 (0.06)	0.00 (0.10)	-0.02 (-0.24, 0.21)	0.88	-0.05 (-0.28, 0.18)	0.66
Word Finding	0.02 (0.08)	-0.10 (0.08)	-0.12 (0.12)	0.13 (-0.14, 0.40)	0.34	0.01 (-0.26, 0.29)	0.92
Spoken Language	-0.03 (0.06)	-0.15 (0.06)	-0.15 (0.09)	0.12 (-0.10, 0.34)	0.28	0.001 (-0.21, 0.22)	0.99
Number Cancellation	-0.15 (0.11)	0.14 (0.11)	-0.17 (0.17)	0.01 (-0.37, 0.40)	0.94	0.30 (-0.08, 0.69)	0.13
Trail Making A	-3.33 (1.98)	-1.09 (1.99)	4.51 (3.10)	-7.84 (-15.04, -0.63)	0.03	-5.60 (-12.82, 1.62)	0.13
Trail Making B	9.12 (6.81)	-4.36 (6.81)	6.17 (10.76)	2.95 (-22.01, 27.90)	0.82	-10.53 (-35.49, 14.43)	0.41
Digit Symbol Substitution	1.51 (1.11)	0.57 (1.17)	-1.33 (1.74)	2.85 (-1.21, 6.90)	0.17	1.90 (-2.22, 6.02)	0.37
WAIS III Digit Span Forward	-1.17 (0.40)	-1.29 (0.40)	-0.77 (0.61)	-0.40 (-1.83, 1.02)	0.58	-0.52 (-1.94, 0.91)	0.48
WAIS III Digit Span Backward	-1.25 (0.31)	-0.95 (0.31)	-0.62 (0.47)	-0.64 (-1.74, 0.47)	0.26	-0.33 (-1.44, 0.77)	0.56
Category Fluency Animals	0.78 (0.51)	-0.38 (0.51)	1.81 (0.76)	-1.03 (-2.82, 0.76)	0.26	-2.19 (-3.98, -0.39)	0.02
Category Fluency Vegetables	0.10 (0.45)	-0.61 (0.44)	1.15 (0.67)	-1.05 (-2.63, 0.53)	0.19	-1.76 (-3.34, -0.19)	0.03

CI, Confidence interval; MD, Mean Difference; SE, Standard error.
Adjusted for age, sex, education, and comorbidities. Marginal means and standard errors obtained from linear mixed models are reported for within-group differences. Between-group differences were assessed using the interaction between time x intervention arm. Lower scores indicate cognitive improvement.

eTable 9. The effect of intervention on ADAS-Cog-Plus scores at 6-month endpoint calculated by cumulatively adding individual Plus items.

© 2023 Montero-Odasso M et al. *JAMA Network Open.*

	Within-group differences					Between-group differences											
	Arm 1	Arm 2	Arm 3	Arm 4	Arm 5	Arm 1 vs Arm 5			Arm 2 vs. Arm 5			Arm 3 vs. Arm 5			Arm 4 vs. Arm 5		
	Mean (SE)	Mean (SE)	Mean (SE)	Mean (SE)	Mean (SE)	MD (95% CI)	p	ES	MD (95% CI)	p	ES	MD (95% CI)	p	ES	MD (95% CI)	p	ES
**Addition of Executive Function tests**																	
ADAS-Cog-13 + TMT-A	-0.35 (0.08)	-0.15 (0.08)	-0.07 (0.08)	-0.16 (0.08)	0.01 (0.09)	-0.36 (-0.59, -0.13)	0.003	0.74	-0.16 (-0.39, 0.08)	0.19	0.31	-0.08 (-0.31, 0.15)	0.51	0.16	-0.17 (-0.40, 0.07)	0.17	0.33
ADAS-Cog-13 + TMT-A + TMT-B	-0.28 (0.08)	-0.12 (0.08)	-0.07 (0.08)	-0.14 (0.08)	-0.02 (0.09)	-0.26 (-0.49, -0.03)	0.03	0.53	-0.10 (-0.33, 0.13)	0.41	0.20	-0.05 (-0.28, 0.18)	0.66	0.10	-0.13 (-0.36, 0.11)	0.30	0.25
ADAS-Cog-13 + TMT-A + TMT-B + DSST	-0.27 (0.08)	-0.13 (0.08)	-0.07 (0.08)	-0.14 (0.08)	-0.01 (0.08)	-0.26 (-0.48, -0.04)	0.02	0.56	-0.12 (-0.34, 0.11)	0.31	0.24	-0.06 (-0.28, 0.16)	0.58	0.13	-0.13 (-0.35, 0.10)	0.27	0.27
ADAS-Cog-13 + TMT-A + TMT-B + DSST + Forward	-0.26 (0.07)	-0.12 (0.08)	-0.06 (0.07)	-0.13 (0.08)	-0.002 (0.08)	-0.26 (-0.47, -0.05)	0.02	0.57	-0.11 (-0.33, 0.10)	0.31	0.24	-0.06 (-0.27, 0.15)	0.59	0.13	-0.12 (-0.34, 0.09)	0.27	0.27
ADAS-Cog-13 + TMT-A + TMT-B + DSST + Forward + Backward	-0.22 (0.07)	-0.08 (0.08)	-0.03 (0.07)	-0.11 (0.08)	0.03 (0.08)	-0.25 (-0.46, -0.04)	0.02	0.57	-0.11 (-0.32, 0.10)	0.33	0.24	-0.06 (-0.27, 0.15)	0.58	0.13	-0.13 (-0.34, 0.08)	0.22	0.29
**Addition of Executive Function tests and Fluency tests**																	
ADAS-Cog-13 + TMT-A + TMT-B + DSST + Forward + Backward + Animal	-0.20 (0.07)	-0.11 (0.07)	-0.02 (0.07)	-0.05 (0.07)	-0.04 (0.07)	-0.16 (-0.35, 0.03)	0.10	0.21	-0.08 (-0.27, 0.11)	0.43	0.04	0.02 (-0.17, 0.21)	0.86	-0.22	-0.01 (-0.20, 0.18)	0.91	-0.18
ADAS-Cog-13 + TMT-A + TMT-B + DSST + Forward + Backward + Animal + Vegetable	-0.16 (0.07)	-0.09 (0.07)	0.02 (0.07)	0.004 (0.07)	-0.07 (0.08)	-0.09 (-0.29, 0.11)	0.39	0.21	-0.02 (-0.22, 0.19)	0.88	0.04	0.09 (-0.11, 0.29)	0.37	-0.22	0.08 (-0.12, 0.28)	0.46	-0.18

Animal, Category Fluency Test Animal; Backward, Digit Span Backward; CI, Confidence interval; DSST, Digit Symbol Substitution Test; Forward, Digit Span Forward; MD, Mean Difference; p, p-value; SE, Standard error; TMT-A, Trail Making Test A; TMT-B, Trail Making Test B; Vegetable, Category Fluency Test Vegetable.
Marginal means and standard errors obtained from linear mixed models are reported for within-group differences. Between-group differences were assessed using the interaction between time x intervention arm. Lower scores indicate cognitive improvement.

eTable 10. The effect of exercise, with (arm 1+2) and without (arm 3+4) cognitive intervention on ADAS-Cog-Plus scores at 6-month endpoint compared to control (arm 5), calculated by cumulatively adding individual Plus items.

	Within-group differences	Between-group differences

© 2023 Montero-Odasso M et al. *JAMA Network Open.*

	Arm 1+2<br>Mean (SE)	Arm 3+4<br>Mean (SE)	Arm 5<br>Mean (SE)	Arm 1+2 vs Arm 5<br>MD (95% CI)	p-value	ES	Arm 3+4 vs. Arm 5<br>MD (95% CI)	p-value	ES
**Additional of Executive tests**									
ADAS-Cog-13 + TMT-A	-0.25 (0.06)	-0.11 (0.06)	0.01 (0.09)	-0.27 (-0.47, -0.06)	0.01	0.41	-0.12 (-0.33, 0.08)	0.25	0.19
ADAS-Cog-13 + TMT-A + TMT-B	-0.20 (0.06)	-0.11 (0.06)	-0.02 (0.09)	-0.18 (-0.39, 0.02)	0.08	0.29	-0.09 (-0.29, 0.12)	0.41	0.14
ADAS-Cog-13 + TMT-A + TMT-B + DSST	-0.20 (0.06)	-0.10 (0.06)	-0.01 (0.08)	-0.19 (-0.39, 0.002)	0.06	0.32	-0.09 (-0.29, 0.10)	0.35	0.16
ADAS-Cog-13 + TMT-A + TMT-B + DSST + Forward	-0.19 (0.05)	-0.09 (0.05)	-0.002 (0.08)	-0.19 (-0.38, 0.0003)	0.05	0.32	-0.09 (-0.28, 0.10)	0.35	0.15
ADAS-Cog-13 + TMT-A + TMT-B + DSST + Forward + Backward	-0.15 (0.05)	-0.07 (0.05)	0.03 (0.08)	-0.18 (-0.36, 0.003)	0.06	0.31	-0.09 (-0.28, 0.09)	0.32	0.17
**Addition of Executive Function tests and Fluency tests**									
ADAS-Cog-13 + TMT-A + TMT-B + DSST + Forward + Backward + Animal	-0.16 (0.05)	-0.03 (0.05)	-0.04 (0.07)	-0.12 (-0.29, 0.05)	0.16	0.23	0.003 (-0.16, 0.17)	0.97	0.007
ADAS-Cog-13 + TMT-A + TMT-B + DSST + Forward + Backward + Animal + Vegetable	-0.13 (0.05)	0.01 (0.05)	-0.07 (0.07)	-0.05 (-0.23, 0.13)	0.56	0.1	0.09 (-0.09, 0.27)	0.34	-0.16

Animal, Category Fluency Test Animal; Backward, Digit Span Backward; CI, Confidence interval; DSST, Digit Symbol Substitution Test; Forward, Digit Span Forward; MD, Mean Difference; p, p-value; SE, Standard error; TMT-A, Trail Making Test A; TMT-B, Trail Making Test B; Vegetable, Category Fluency Test Vegetable.
Marginal means and standard errors obtained from linear mixed models are reported for within-group differences. Between-group differences were assessed using the interaction between time x intervention arm. Lower scores indicate cognitive improvement.

© 2023 Montero-Odasso M et al. *JAMA Network Open.*

eTable 11. Change in ADAS-Cog-13 and ADAS-Cog-Plus at 12-month follow-up.

	Within-group difference					Between-group difference							
	Arm 1	Arm 2	Arm 3	Arm 4	Arm 5	Arm 1 vs Arm 5		Arm 2 vs. Arm 5		Arm 3 vs. Arm 5		Arm 4 vs. Arm 5	
	Mean (SE)	Mean (SE)	Mean (SE)	Mean (SE)	Mean (SE)	MD (95% CI)	p	MD (95% CI)	p	MD (95% CI)	p	MD (95% CI)	p
**ADAS-Cog-13**													
Baseline to 6 Month	-2.42 (0.70)	-2.14 (0.73)	-0.39 (0.69)	-1.34 (0.73)	0.24 (0.76)	-2.66 (-4.68, -0.64)	0.01	-2.38 (-4.44, -0.31)	0.02	-0.63 (-2.65, 1.39)	0.54	-1.57 (-3.64, 0.49)	0.13
6 Month to 12 Month	0.52 (0.71)	-0.14 (0.77)	-0.84 (0.72)	0.61 (0.75)	-1.26 (0.80)	1.78 (-0.31, 3.88)	0.10	1.12 (-1.05, 3.30)	0.31	0.43 (-1.68, 2.53)	0.69	1.87 (-0.30, 4.03)	0.09
Baseline to 12 Month	-1.90 (0.70)	-2.28 (0.76)	-1.22 (0.71)	-0.73 (0.75)	-1.02 (0.79)	-0.88 (-2.96, 1.21)	0.41	-1.26 (-3.42, 0.90)	0.25	-0.20 (-2.29, 1.89)	0.85	0.29 (-1.86, 2.44)	0.79
**ADAS-Cog-Plus**													
Baseline to 6 Month	-0.16 (0.08)	-0.09 (0.08)	0.02 (0.08)	0.005 (0.08)	-0.07 (0.08)	-0.09 (-0.31, 0.13)	0.43	-0.02 (-0.24, 0.21)	0.89	0.09 (-0.13, 0.31)	0.41	0.08 (-0.15, 0.30)	0.50
6 Month to 12 Month	0.04 (0.08)	0.02 (0.08)	0.06 (0.08)	0.07 (0.08)	0.03 (0.09)	0.009 (-0.24, 0.21)	0.94	-0.01 (-0.25, 0.22)	0.91	0.03 (-0.20, 0.26)	0.81	0.04 (-0.20, 0.28)	0.74
Baseline to 12 Month	-0.12 (0.08)	-0.07 (0.08)	0.08 (0.08)	0.08 (0.08)	-0.04 (0.09)	-0.08 (-0.31, 0.15)	0.49	-0.03 (-0.27, 0.21)	0.80	0.12 (-0.11, 0.35)	0.31	0.12 (-0.12, 0.35)	0.33

CI, Confidence interval; MD, Mean Difference; p, p-value; SE, Standard error.
Marginal means and standard errors obtained from linear mixed models are reported for within-group differences. Between-group differences were assessed using the interaction between time x intervention arm. Lower scores indicate cognitive improvement.

© 2023 Montero-Odasso M et al. *JAMA Network Open.*

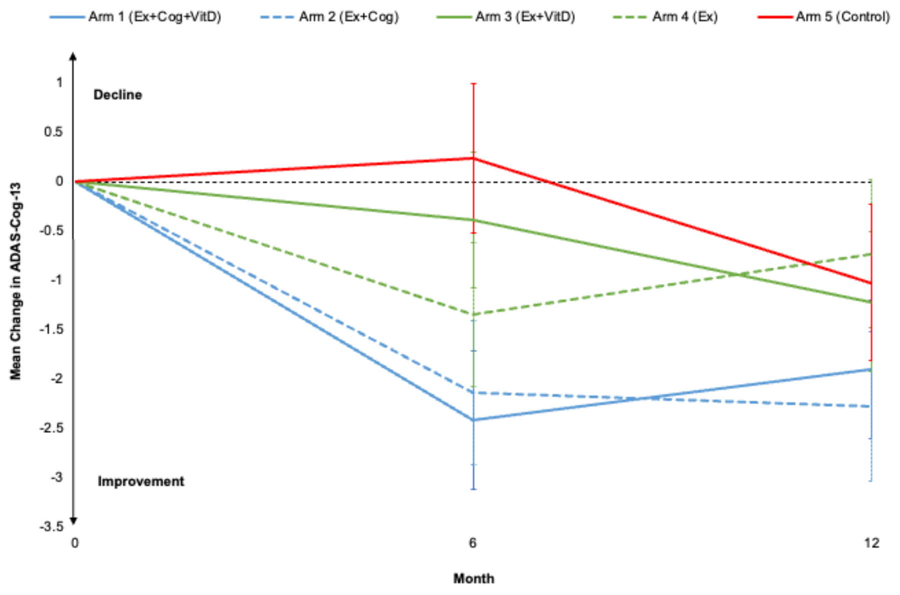

eFigure 1. Change in ADAS-Cog-13 scores from baseline to 12 months across 5 arms.
This figure shows estimated mean change in ADAS-Cog-13 from baseline to month 12 per arm. Lower scores indicate better cognitive performance. Error bars represent standard errors.

© 2023 Montero-Odasso M et al. *JAMA Network Open.*

eTable 12. The effect of exercise, with (arm 1+2) and without (arm 3+4) cognitive intervention at 12-month follow-up, compared to control (arm 5) using "at the margin" analysis.

	Within-group difference			Between-group difference			
	Exercise and Cognitive Intervention (Arm 1+2)	Exercise (Arm 3+4)	Control (Arm 5)	Exercise and Cognitive Intervention (Arm 1+2) vs. Control (Arm 5)		Exercise (Arm 3+4) vs. Control (Arm 5)	
	Mean (SE)	Mean (SE)	Mean (SE)	MD (95% CI)	p-value	MD (95% CI)	p-value
**ADAS-Cog-13**							
Baseline to 6 Month	-2.29 (0.50)	-0.84 (0.50)	0.24 (0.75)	-2.52 (-4.30, -0.74)	0.006	-1.08 (-2.86, 0.71)	0.24
6 Month to 12 Month	0.22 (0.52)	-0.15 (0.52)	-1.26 (0.80)	1.48 (-0.39, 3.35)	0.12	1.11 (-0.76, 2.98)	0.24
Baseline to 12 Month	-2.07 (0.52)	-0.99 (0.52)	-1.02 (0.79)	-1.04 (-2.90, 0.81)	0.27	0.03 (-1.82, 1.89)	0.97
**ADAS-Cog-Plus**							
Baseline to 6 Month	-0.13 (0.05)	0.01 (0.05)	-0.07 (0.08)	-0.05 (-0.25, 0.14)	0.59	0.09 (-0.11, 0.28)	0.39
6 Month to 12 Month	0.03 (0.06)	0.07 (0.06)	0.03 (0.09)	-0.002 (-0.21, 0.20)	0.98	0.03 (-0.17, 0.24)	0.75
Baseline to 12 Month	-0.10 (0.06)	0.08 (0.06)	-0.04 (0.09)	-0.06 (-0.26, 0.15)	0.59	0.12 (-0.08, 0.32)	0.25

CI, Confidence interval; MD, Mean Difference; SE, Standard error.
Marginal means and standard errors obtained from linear mixed models are reported for within-group differences. Between-group differences were assessed using the interaction between time x intervention arm. Lower scores indicate cognitive improvement.

© 2023 Montero-Odasso M et al. *JAMA Network Open.*

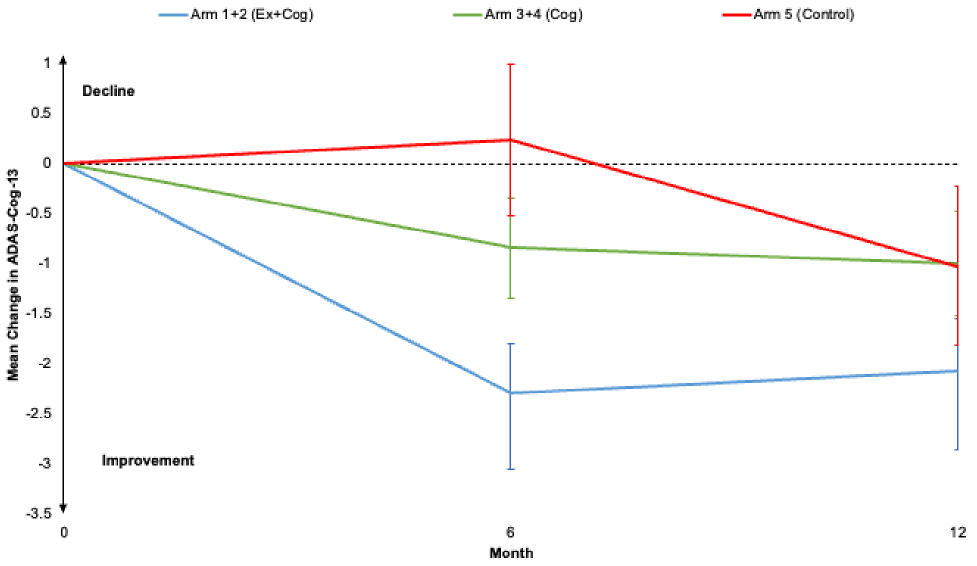

eFigure 2. Change in ADAS-Cog-13 scores from baseline to 12 months

This figure shows estimated mean change in ADAS-Cog-13 from baseline to month 12 in arm 1+2 (exercise and cognitive intervention)), arm 3+4 (exercise only), and arm 5 (control). Lower scores indicate better cognitive performance. Error bars represent standard errors.

© 2023 Montero-Odasso M et al. *JAMA Network Open.*

eTable 13. Exercise and physical activity level reported at Month 12

	Overall (N=175)	Arm 1 (N=34)	Arm 2 (N=35)	Arm 3 (N=37)	Arm 4 (N=35)	Arm 5 (N=34)
Walking outside home*						
Never	10 (5.7%)	4 (11.8%)	1 (2.9%)	1 (2.7%)	2 (5.7%)	2 (5.9%)
Seldom (1-2 days)	13 (7.4%)	1 (2.9%)	2 (5.7%)	2 (5.4%)	4 (11.4%)	4 (11.8%)
Sometimes (3-4 days)	30 (17.1%)	8 (23.5%)	5 (14.3%)	8 (21.6%)	4 (11.4%)	5 (14.7%)
Often (5-7 days)	73 (41.7%)	15 (44.1%)	15 (42.9%)	13 (35.1%)	16 (45.7%)	14 (41.2%)
Light activities†						
Never	111 (63.4%)	23 (67.6%)	21 (60.0%)	20 (54.1%)	24 (68.6%)	23 (67.6%)
Seldom (1-2 days)	5 (2.9%)	1 (2.9%)	0 (0%)	2 (5.4%)	0 (0%)	2 (5.9%)
Sometimes (3-4 days)	7 (4.0%)	2 (5.9%)	1 (2.9%)	2 (5.4%)	2 (5.7%)	0 (0%)
Often (5-7 days)	1 (0.6%)	1 (2.9%)	0 (0%)	0 (0%)	0 (0%)	0 (0%)
Moderate activities‡						
Never	108 (61.7%)	22 (64.7%)	18 (51.4%)	21 (56.8%)	23 (65.7%)	24 (70.6%)
Seldom (1-2 days)	6 (3.4%)	2 (5.9%)	2 (5.7%)	0 (0%)	1 (2.9%)	1 (2.9%)
Sometimes (3-4 days)	7 (4.0%)	2 (5.9%)	1 (2.9%)	2 (5.4%)	2 (5.7%)	0 (0%)
Often (5-7 days)	2 (1.1%)	1 (2.9%)	0 (0%)	1 (2.7%)	0 (0%)	0 (0%)
Strenuous activities§						
Never	103 (58.9%)	24 (70.6%)	19 (54.3%)	15 (40.5%)	22 (62.9%)	23 (67.6%)
Seldom (1-2 days)	10 (5.7%)	2 (5.9%)	1 (2.9%)	4 (10.8%)	3 (8.6%)	0 (0%)
Sometimes (3-4 days)	10 (5.7%)	2 (5.9%)	2 (5.7%)	4 (10.8%)	1 (2.9%)	1 (2.9%)
Often (5-7 days)	3 (1.7%)	0 (0%)	1 (2.9%)	1 (2.7%)	0 (0%)	1 (2.9%)
Muscle strength and endurance exercises‖						
Never	87 (49.7%)	24 (70.6%)	14 (40.0%)	15 (40.5%)	16 (45.7%)	18 (52.9%)
Seldom (1-2 days)	14 (8.0%)	1 (2.9%)	3 (8.6%)	4 (10.8%)	2 (5.7%)	4 (11.8%)
Sometimes (3-4 days)	18 (10.3%)	2 (5.9%)	5 (14.3%)	2 (5.4%)	7 (20.0%)	2 (5.9%)
Often (5-7 days)	7 (4.0%)	1 (2.9%)	1 (2.9%)	3 (8.1%)	1 (2.9%)	1 (2.9%)

Note: Light activities include light exercise, bowling, golf with a cart, shuffleboard, tai chi, and yoga; Moderate activities include moderate exercise, ballroom dancing, golf without a cart, and biking; Strenuous activities include strenuous exercise, jogging, swimming, biking, rowing, and squash; Muscle strength and endurance exercises include lifting weights, and push ups

*Data are missing for 49 participants

†Data are missing for 51 participants

‡Data are missing for 52 participants

§Data are missing for 49 participants

‖Data are missing for 49 participants

© 2023 Montero-Odasso M et al. *JAMA Network Open.*

eTable 14. Self-reported adverse events and health-care follow-up diagnosis during the study.

	Total (n=175)	Arm 1 (n=34)	Arm 2 (n=35)	Arm 3 (n=37)	Arm 4 (n=35)	Arm 5 (n=34)
**Participants with self-reported adverse events during the study**						
All adverse events	52 (30%)	8 (24%)	13 (37%)	10 (27%)	15 (43%)	6 (18%)
Musculoskeletal pain	24 (14%)	4 (12%)	5 (14%)	4 (11%)	8 (23%)	3 (9%)
Fall (tripped or slipped)	6 (3%)	1 (3%)	0	1 (3%)	2 (6%)	2 (6%)
Numbness	4 (2%)	0	2 (6%)	1 (3%)	1 (3%)	0
Dizziness	3 (2%)	2 (6%)	1 (3%)	0	0	0
Others*	11 (6%)	1 (3%)	4 (11%)	3 (8%)	2 (6%)	1 (3%)
**Participants with Health-care follow-up diagnosis during the study†**						
Minor Stroke	2 (1%)	0	1 (3%)	0	1 (3%)	0
Hip fracture	1 (<1%)	0	0	1 (3%)	0	0
Breast cancer	1 (<1%)	0	0	0	1 (3%)	0

*Others include: systolic blood pressure readings too high, knee pain, hip pain, back pain, chest discomfort, pressure in the head, profuse sweating, headache, head injury in garage, pneumonia, shingles, herpes zoster.
†Diagnoses that occurred within the study period. These four adverse events were not intervention-related.

© 2023 Montero-Odasso M et al. *JAMA Network Open.*

eReferences

1. Albert MS, DeKosky ST, Dickson D, et al. The diagnosis of mild cognitive impairment due to Alzheimer's disease: recommendations from the National Institute on Aging-Alzheimer's Association workgroups on diagnostic guidelines for Alzheimer's disease. *Alzheimers Dement.* 2011;7(3):270-279.
2. Bherer L, Kramer AF, Peterson MS, Colcombe S, Erickson K, Becic E. Training effects on dual-task performance: are there age-related differences in plasticity of attentional control? *Psychol Aging.* 2005;20(4):695-709.
3. Bherer L, Kramer AF, Peterson MS, Colcombe S, Erickson K, Becic E. Testing the limits of cognitive plasticity in older adults: application to attentional control. *Acta Psychol (Amst).* 2006;123(3):261-278.
4. Bherer L, Kramer AF, Peterson MS, Colcombe S, Erickson K, Becic E. Transfer effects in task-set cost and dual-task cost after dual-task training in older and younger adults: further evidence for cognitive plasticity in attentional control in late adulthood. *Exp Aging Res.* 2008;34(3):188-219.
5. Li KZ, Roudaia E, Lussier M, Bherer L, Leroux A, McKinley PA. Benefits of cognitive dual-task training on balance performance in healthy older adults. *J Gerontol A Biol Sci Med Sci.* 2010;65(12):1344-1352.
6. Lussier M, Brouillard P, Bherer L. Limited Benefits of Heterogeneous Dual-Task Training on Transfer Effects in Older Adults. *J Gerontol B Psychol Sci Soc Sci.* 2017;72(5):801-812.
7. Stanziano DC, Roos BA, Perry AC, Lai S, Signorile JF. The effects of an active-assisted stretching program on functional performance in elderly persons: a pilot study. *Clin Interv Aging.* 2009;4:115-120.
8. Venning G. Recent developments in vitamin D deficiency and muscle weakness among elderly people. *BMJ.* 2005;330(7490):524-526.
9. Vieth R. The mechanisms of vitamin D toxicity. *Bone Miner.* 1990;11(3):267-272.
10. Vieth R, Chan PC, MacFarlane GD. Efficacy and safety of vitamin D3 intake exceeding the lowest observed adverse effect level. *Am J Clin Nutr.* 2001;73(2):288-294.

© 2023 Montero-Odasso M et al. *JAMA Network Open.*
