# Supplementary material for: Effects of Exercise Alone or Combined With Cognitive Training and Vitamin D Supplementation to Improve Cognition in Adults With Mild Cognitive Impairment: A Randomized Clinical Trial
Source: JAMA Netw Open. 2023 Jul 20;6(7):e2324465. doi: 10.1001/jamanetworkopen.2023.24465 (PMC10359965; doi:10.1001/jamanetworkopen.2023.24465)
Supplement: Supplement 3. — Nonauthor Collaborators [file jamanetwopen-e2324465-s003.pdf]

Supplemental Online Content: Nonauthor Collaborators
*First name, last name, and suffix (if applicable) are required and will appear in PubMed.

*Group Name(s): Canadian Gait and Cognition Network							
***First Name and Middle Initial(s)**	***Last Name**	***Suffix (eg, Jr, III)**	Academic Degrees	Institution	Location (city, state/province, country)	Role or Contribution, eg, chair, principal investigator	Group (if more than 1 Group listed in the byline) and/or Subgroup (eg, Steering Committee)
Bradford J.	McFadyen		PhD	Department of Rehabilitation, Universite Laval	Quebec City, QB, Canada	Investigator and Member	CCNA Team 12
Cindy	Barha		PhD	Aging, Mobility, and Cognitive Health Lab, Faculty of Medicine. University of British Columbia	Vancouver, BC, Canada	Team member	CCNA Team 12
Christopher	McGibbon		PhD	Faculty of Kinesiology and Institute of Biomedical Engineering. University of New Brunswick	Fredericton, NB, Canada	Investigator and Member	CCNA Team 12
