# Supplementary material for: Effects of Exercise Alone or Combined With Cognitive Training and Vitamin D Supplementation to Improve Cognition in Adults With Mild Cognitive Impairment: A Randomized Clinical Trial
Source: JAMA Netw Open. 2023 Jul 20;6(7):e2324465. doi: 10.1001/jamanetworkopen.2023.24465 (PMC10359965; doi:10.1001/jamanetworkopen.2023.24465)
Supplement: Supplement 4. — Data Sharing Statement [file jamanetwopen-e2324465-s004.pdf]

Data Sharing Statement

Montero-Odasso. Effects of Exercise Alone or Combined with Cognitive Training and Vitamin D Supplementation to Improve Cognition in Adults with Mild Cognitive Impairment. *JAMA Netw Open*. Published July 20, 2023. doi:10.1001/jamanetworkopen.2023.24465

Data
Data available: Yes
Data types: Deidentified participant data
How to access data: SYNERGYC Trial data sharing will follow guidelines from the Canadian Consortium on Neurodegeneration in Aging (CCNA; https://cena-cenv.ca/w-content/uploads/2022/07/CCNA-Publications-and-data-access-policy-v-1.4.pdf). After the embargo period, deidentified data could be made available for researchers who submit a proposal to Principal Investigator Dr. Manuel Montero-Odasso (mmontero@uwo.ca).
When available: beginning date: 04-07-2028

Supporting Documents
Document types: None

Additional Information
Who can access the data: Researchers who submit a proposal to Dr. Manuel Montero-Odasso at mmontero@uwo.ca Types of analyses: For specific hypothesis or meta-anlayses.
Types of analyses: After approval of the proposal
Mechanisms of data availability: After approval of proposal
Any additional restrictions: N/A
